# HOG1 Mitogen-Activated Protein Kinase Pathway–Related Autophagy Induced by H_2_O_2_ in *Lentinula edodes* Mycelia

**DOI:** 10.3390/jof9040413

**Published:** 2023-03-28

**Authors:** Dong Yan, Yangyang Fan, Shuang Song, Yuan Guo, Yu Liu, Xiaoling Xu, Fang Liu, Qi Gao, Shouxian Wang

**Affiliations:** 1 Institute of Plant Protection, Beijing Academy of Agriculture and Forestry Sciences, Beijing Engineering Research Center for Edible Mushroom, 9 Shuguang Garden Zhonglu, Haidian District, Beijing 100097, China; 2 College of Agriculture and Food Engineering, Baise University, 21 Zhongshan Second Street, Youjiang District, Baise 533000, China

**Keywords:** *Lentinula edodes*, H_2_O_2_, autophagy, LeATG8, LeHOG1

## Abstract

Mycelial ageing is associated with ROS and autophagy in *Lentinula edodes*. However, the underlying cellular and molecular mechanisms between ROS and autophagy remain obscure. This study induced autophagy in *L. edodes* mycelia through exogenous H_2_O_2_ treatment. Results showed that 100 μM H_2_O_2_ treatment for 24 h significantly inhibited mycelial growth. H_2_O_2_ caused the depolarisation of MMP and accumulation of TUNEL-positive nuclei, which was similar to the ageing phenotype of *L. edodes* mycelia. Transcriptome analysis showed that differentially expressed genes were enriched in the mitophagic, autophagic, and MAPK pathways. *LeAtg8* and *LeHog1* were selected as hub genes. RNA and protein levels of LeATG8 increased in the H_2_O_2_-treated mycelia. Using fluorescent labelling, we observed for the first time the classic ring structure of autophagosomes in a mushroom, while 3D imaging suggested that these autophagosomes surrounded the nuclei to degrade them at specific growth stages. Phospho-LeHOG1 protein can translocate from the cytoplasm to the nucleus to regulate mycelial cells, resisting ROS-induced oxidative stress. Furthermore, LeATG8 expression was suppressed when LeHOG1 phosphorylation was inhibited. These results suggest that the LeATG8-dependent autophagy in *L. edodes* mycelial is closely associated with the activity or even phosphorylation of LeHOG1.

## 1. Introduction

Autophagy is an evolutionarily conserved process in eukaryotes [1]. Due to its significance in maintaining intracellular physiological balance and stress resistance, autophagy has become a research hotspot. In 1997, the first autophagy-related gene *Atg1* was discovered in yeast, and since then, 42 *Atg* genes have been reported in eukaryotes to be responsible for the formation and regulation of autophagosomes [2]. The ubiquitin-like protein encoded by the *Atg8* gene (the homologous protein in mammalian cells is LC3) is a key component of autophagosomes [3]. ATG8 can bind to phosphatidylethanolamine (PE) under the action of ubiquitin activating enzyme E1, ubiquitin-conjugating enzyme E2, and ubiquitin ligase E3, thereby anchoring on the autophagosome membrane and outer membrane to help facilitate the extension and expansion of autophagosomes [1]. Therefore, ATG8 protein is widely used in labelling autophagosomes and evaluating the degree of autophagy [4]. At the late stage of autophagy, autophagosomes fuse with vacuoles to form autophagic lysosomes. The outer-membrane-bound ATG8 is released into the cytoplasm for repeated use, while the inner-membrane-bound ATG8 and its inclusions are degraded by vacuoles.

ATG8 protein plays a key role in the growth, development, cell differentiation, and secondary metabolism of fungi [1]. In *Magnaporthe oryzae*, ATG8-mediated autophagy is necessary for conidial formation and pathogenicity. Deletion of *Atg8* causes a large decrease in conidia and loss of infection function [5]. FgATG8 deletion in *Fusarium graminearum* inhibits mycelial growth and reproductive function [6]. In *Ustilaginoidea virens*, UvATG8 is required for mycelial growth, stress response, conidiation, secondary sporulation, and pathogenicity [7]. In macrofungi, the related research on ATG8 is mostly limited to the expression level. For example, in *Agaricus bisporus*, fluorescence quantitative analysis showed that *Atg8* gene was highly expressed in fruiting bodies and mycelia following ethylene treatment, which suggests the involvement of *Atg8* gene in the maturation of fruiting bodies and senescence of mycelia [8].

Similarly, several autophagy-related cell structures, differentially expressed genes, proteins, and metabolites were found in the study of *Lentinula edodes* ageing and pigmentation of mycelium. *L. edodes* is a popular commercial mushroom in Asia, owing to its unique taste/flavour, high carbohydrate/fibre contents, and low calories [9]. In the production of *L. edodes*, the pigment mycelium will form after long-term culture. The age and pigment of *L. edodes* mycelium affect the quality and yield of its fruiting body. The ultrastructure of aged *L. edodes* mycelia has been previously analysed using transmission electron microscopy, which showed autophagosome-like structures containing cytoplasmic components such as mitochondria or endoplasmic reticulum wrapped by a double-layer membrane in aged cells [10]. At the transcriptome level, there are various differentially expressed genes of autophagy and age-dependent response in *L. edodes* mycelia and fruiting bodies postpigmentation [11,12,13]. At the proteome level, the expression of autophagy key factor vacuolar protein sorting (VPS1, VPS27, and VPS34) vacuolar membrane protein (VAC8) is significantly increased in *L. edodes* pigment mycelium [14,15]. At the metabolomics level, the differential metabolites—asparagine, proline, leucine, valine, glutamine, and palmitic acid—are related to autophagy, apoptosis, and reactive oxygen species (ROS)-mediated oxidative stress [16]. In addition, the expression of genes that resist or respond to ROS during the development of pigment mycelium is consistent with the expression trend of autophagy-related genes [17]. Moreover, the expression of LeATG8 protein significantly increased in long-term culture of *L. edodes* mycelium, and the activity of antioxidant enzymes also increased (Gao et al., 2023). This suggests that both autophagy and oxidative stress are involved in the ageing process of *L. edodes* mycelium. However, the relationship between oxidative stress and LeATG8-mediated autophagy in *L. edodes* mycelium warrants further studies.

Therefore, this study elucidates the relationship between oxidative stress and autophagy in *L. edodes* mycelia. We induced oxidative stress in mycelia through exogenous H_2_O_2_ application and assessed the changes regulated via autophagy. Using transcriptome, Western blot, and immunofluorescence analyses, we studied the expression and subcellular localisation of ROS and autophagy-related proteins and revealed that high-osmolarity glycerol protein (LeHOG1) activation/phosphorylation and autophagy-related protein 8 (LeATG8) expression cooperatively modulate ROS-induced autophagy. Clarifying the relationship between autophagy and oxidative stress can provide a preliminary basis for analysing the ageing and pigmentation mechanism of *L. edodes* strains.

## 2. Materials and Methods

### 2.1. Fungal Strains and Induction Conditions

**Figure 1:**
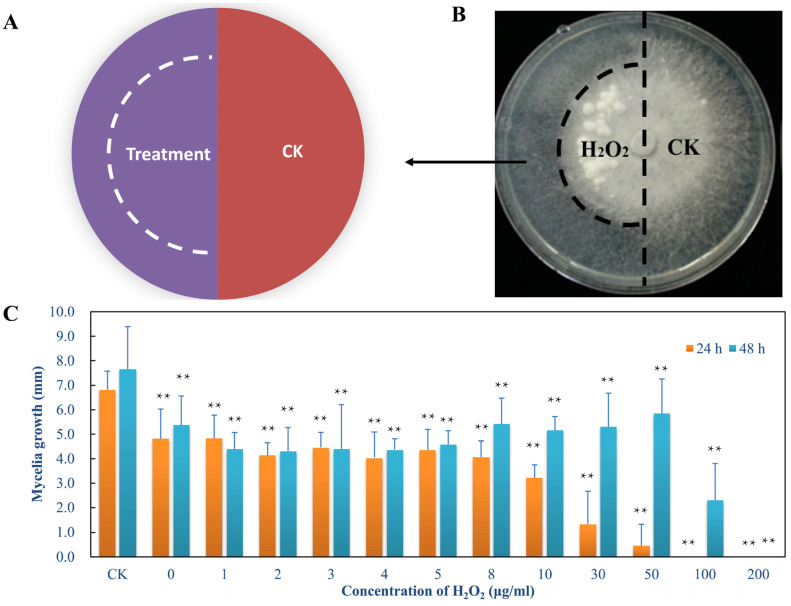
Hydrogen-peroxide-induced mycelial culture. (**A**) Schematic illustration of *L. edodes* mycelia. (**B**) Mycelial morphology after 20 days of treatment. (**C**) Mycelial growth rate after 24 and 48 h of treatment. Mean ± SD, *n* = 8. ** *p* < 0.01 vs. CK.

*L. edodes* strain 0912 (JZB2102217), cultivated in northern China, was used in the present study. Mycelia were inoculated on quantitative potato dextrose agar (PDA, 20 mL per 9 cm plate) overlaid with cellophane and cultured at 22 °C for 5 days. To analyse the association between autophagy and ROS, we used H_2_O_2_ as an inducer. *L. edodes* mycelia were grown in the absence or presence of 100 μL of H_2_O_2_. In the induction test, each plate was divided into two parts, and an inducer was added to the apical cells of one part (Figure 1A). To optimise H_2_O_2_ concentration, apical mycelia cultured as described above were treated with 1, 2, 3, 4, 5, 8, 30, 50, 100, and 200 μM H_2_O_2_. After culturing for 24 and 48 h, the growth rate, mitochondrial membrane potential (MMP), and nuclei phenotype of the mycelia under each treatment were assayed to evaluate treatment effects and mitochondrial function. The fluorochrome JC-1 (Beyotime Biotechnology, Shanghai, China) was used to estimate MMP. *L. edodes* mycelia were treated with 10 μM JC-1 in the dark for 30 min, washed twice with JC-1 buffer, and observed with green and red fluorescence channels under an IX71 inverted fluorescence microscope (Olympus, Tokyo, Japan). DAPI (Sigma Aldrich, St. Louis, MO, USA) and terminal deoxynucleotidyl transferase dUTP nick-end labelling (TUNEL) fluorescence staining (Beyotime Biotechnology, Shanghai, China) were performed to detect total nuclei and DNA fragments [18]. The results of nuclear labelling were observed under the blue fluorescence and green fluorescence channel using a fluorescence microscope, for DAPI and TUNEL, respectively. Each experiment was performed in triplicate.

### 2.2. RNA-Seq and Transcriptome Analysis

Five-day-old cultures of *L. edodes* mycelia were inoculated with 100 μM H_2_O_2_ in PDA-cellophane at 22 °C and sampled at 0, 3, and 24 h intervals. Apical cells were collected, flash frozen using liquid nitrogen, and stored at −80 °C for transcriptome analysis.

The RNA of three mycelia samples (three independent replicates per sample) were extracted using TRIZOL^®^ Reagent (Invitrogen, Waltham, MA, USA). The quality and integrity of the RNA were evaluated using a NanoDrop 2000 spectrophotometer (Thermo Fisher Scientific, Waltham, MA, USA) and an Agilent 2100 bioanalyser (Agilent Technologies, Santa Clara, CA, USA), respectively. The library construction and RNA-seq were conducted by Novogene Bioinformatics Technology Co., Ltd. (Beijing, China). cDNA libraries were constructed using NEBNext^®^ Ultra™ RNA Library Prep Kits for Illumina^®^ (NEB, Ipswich, MA, USA). After the library was qualified, different libraries were pooled according to the effective concentration and the machine data and then sequenced using an Illumina platform with 150 bp paired-end reads.

The quality control of raw data was performed using the FastQC plugin of TBTools v1.09876 [19]. After removing low-quality pair reads and adaptor contamination using the Trimmomatic plugin, we spliced the clean reads and aligned them to the reference *L. edodes* strain SP3 (https://ngdc.cncb.ac.cn/bioproject/browse/PRJCA007678, accessed on 27 December, 2021) using the kallisto plugin. The gene expression level was measured based on fragments per kilobase of transcript per million fragments mapped. Differentially expressed genes (DEGs) were screened using DESeq2 (|log2 (FoldChange)| > 0, *p* < 0.01) and then enriched using gene ontology (GO) terms (*p* < 0.05) and Kyoto Encyclopedia of Genes and Genomes (KEGG) pathways. Real-time qPCR of LeAtg8 and LeHog1 was performed using TB Green^®^ Premix Ex Taq™ II (Tli RNaseH Plus, TaKaRa, Japan) on an ABI 7500 real-time PCR system (Applied Biosystems, USA) [20]. The primers of *LeAtg8* were 5′-ATCGTATTCCTGTAATCTGCG-3′ (forward) and 5′- TAGACGAACTGCCCCACA-3′ (reverse). The primers of *LeHog1* were 5′-CTATCACGACCCCACCGATG-3′ (forward) and 5′- TGGTCCTTCGGTTAGGCCTA-3′ (reverse). β-Tubulin was used as the reference gene [21].

### 2.3. Antioxidant Activity and ROS Content of Induced and Inhibited Mycelia

A p38-MAPK inhibitor of SB203580 (10 μM) was used to pretreat the cellophane and identify the function of LeHOG1 in mycelial ageing. The total protein of *L. edodes* mycelia was extracted using a protein extraction kit (Bestbio, China) for microfungi. Total protein concentration was detected using the BCA protein assay kit (Beyotime Biotechnology, Shanghai, China). Superoxide dismutase (SOD) activity was measured using its corresponding kit (Nanjing Jiancheng Bioengineering Institute, Nanjing, China).

Fresh *L. edodes* mycelia under different treatments were washed with PBS buffer three times and incubated with 10 µM 2,7-dichlorodi-hydrofluorescein diacetate (DCFH-DA, Beyotime Biotechnology, Shanghai, China) for 30 min at 25 °C in the dark. Images were obtained under a fluorescence microscope and analysed using Image J as previously described [22]. 

### 2.4. Antibody Preparation and Western Blotting

To obtain LeATG8-specific rabbit polyclonal antibody, we amplified *LeAtg8* from the cDNA of *L. edodes* mycelia using the primer pair of 5′-GAATTCATGAGGTCAAAGTTCAAGGACGAGC-3′ (forward) and 5′-CTCGAGTCATGCATCCATCGGCAGCT-3′ (reverse). A pET-28a(+)-LeAtg8 recombinant plasmid was constructed and transformed into Escherichia coli BL21 (DE3). After ultrasonication, LeATG8 in the supernatants was eluted with a buffer containing 500 mM imidazole on a Ni-Sepharose column. An anti-LeATG8 antibody was raised in rabbits by SanQ (Beijing, China) Biotechnology Co., Ltd. (Beijing, China). 

The loading amount of each sample was normalised according to the protein concentration of the sample. The loading protein was separated using 12% SDS-PAGE and transferred onto a polyvinylidene fluoride membrane at 120 mA for 1 h. After blocking with 10% milk, the membrane was incubated with the anti-LeATG8 primary antibody and goat anti-rabbit IgG H&L secondary antibody (Abcam, Cambridge, MA, USA) in 1:1000 dilutions overnight at 4 °C. The immunoreactive bands were detected using the Chemidoc imaging system (Bio-Rad, Hercules, CA, USA). 

Phospho-p38 MAPK (Thr180/Tyr182) (D3F9) XP^®^ rabbit antibody (Cell Signaling Technology, Danvers, MA, USA) was detected to analyse the phosphorylation level of LeHOG1, whereas p38 MAPK (D13E1) XP^®^ rabbit antibody (Cell Signaling Technology, Danvers, MA, USA) was used as the control.

### 2.5. Freezing Microtome Section and Immunofluorescence

*L. edodes* mycelia were washed twice with PBS and then fixed with glutaraldehyde (2.5% in PBS) for 20 min at 4 °C. After washing with PBS, the samples were immersed in protectants (5% trehalose and 10% glycerol) under vacuum and then incubated for 1 h. The embedded samples were cut into serial coronal sections (6 μm thick) using a freezing microtome (Leica CM1990, Nussloch, Germany). The sections were postfixed with paraformaldehyde (4% in PBS) for 30 min at 4 °C and then washed three times with PBS containing 0.1% Triton X-100 (PBST). Subsequently, the sections were incubated in immunol staining blocking buffer (Beyotime Biotechnology, Shanghai, China) for 1 h. After the sections were incubated at 4 °C overnight with anti-LeATG8 antibody, anti-P38 antibody, or anti-phospho-P38 antibody (1:1000 dilution), they were washed three times with PBST. The sections were incubated with goat anti-rabbit Alexa Fluor 488 dye (1:500 in PBST) for 2 h, counterstained with 8 µg/mL 4′,6-diamidino-2-phenylindole (DAPI; Sigma Aldrich, St. Louis, MO, USA), and then mounted using Mowiol solution (10% Mowiol 4-88 and 20% glycerol in PBS). The sections were observed with a confocal microscope (LSM 900 with Airyscan2, Zeiss, Germany). Three repeated immunofluorescence stainings for each treated sample were performed. Each section observed 20 fields, and for each field, 8–16 images were taken for splicing. In order to observe all levels of mycelium, 40 images must be taken continuously by Z-stack. Z-stack images were taken in Airyscan mode in multiple fluorescence channel images with brightfield taken in confocal mode. We used the ZEN blue software surface mode of 3D VisArt for 3D imagination. 

### 2.6. Statistical Analysis and Data Availability

The SOD activity and DCFH-DA-labelled ROS contents obtained in this study are presented as means ± standard deviation (SD). The numbers of biological replicates in each experiment are noted in the figure legends. Statistical analysis was performed using ANOVA. The significant differences between means were determined by Duncan’s multiple-range test. Unless otherwise stated, the differences were considered statistically significant at *p* < 0.01.

All data generated or analysed during this study are included in this article and its supplementary information files. The raw sequencing data of RNA-seq are available at the National Genomics Data Center, China National Center for Bioinformation, under BioProject ID PRJCA012101 (https://ngdc.cncb.ac.cn/bioproject/browse/PRJCA012101, accessed on 17 March 2023).

## 3. Results

### 3.1. H_2_O_2_ Treatment Altered Mycelial Morphology and Microstructure

In the long-term cultured ageing mycelium of *L. edodes*, with the increase of autophagy protein LeATG8 expression, PCD phenomena, such as weakened mycelium growth, mycelium agglutination, decreased MMP, and increased TUNEL-positive nuclei, were observed (Gao et al., 2023). The above phenotypic changes were used as screening markers to determine the optimal time and concentration of H_2_O_2_ treatment when establishing an induced autophagy model of *L. edodes*.

First, we used a high concentration of H_2_O_2_ (200 μM) to treat the mycelium and cultured it for 20 days (Figure 1A,B). Mycelial agglutination appeared earlier on the surface of the mycelium after treatment. The results showed that a high concentration of hydrogen peroxide could accelerate the senescence of *L. edodes* mycelium. Treatment with H_2_O_2_ at 1–200 μM inhibited mycelial growth after 24 h. High H_2_O_2_ concentrations of 100 and 200 μM significantly prevented fungal growth (Figure 1C). After 48 h, the growth of mycelia under 100 μM H_2_O_2_ treatment was recovered, whereas that of mycelia under 200 μM H_2_O_2_ treatment was inhibited. 

MMP was determined using JC-1 staining to measure the mitochondrial depolarisation induced by H_2_O_2_ (Figure S1). H_2_O_2_ increased the oxidative stress and induced ageing by initiating mitochondrial dysfunctions. JC-1 exhibits red fluorescence in polarised healthy mitochondria and green fluorescence in depolarised mitochondria. In the control group, JC-1 showed intense red fluorescence and weak green fluorescence. The fluorescence of JC-1 changed from red to green after treatment of the mycelia with a high (100 μM) or low (8 μM) concentration of H_2_O_2_. This result suggests mitochondrial damage and depolarisation caused by H_2_O_2_. Therefore, we selected 100 μM H_2_O_2_ for subsequent experiments. We observed the mycelial cell structure after treatment with 100 μM H_2_O_2_. After 3 h of treatment, MMP fluorescence showed green fluorescence indicating that the mitochondrial membrane potential had decreased (Figure S2A,B). However, the results of TUNEL and DAPI combined labelling showed that the hyphae after 3 h of treatment were mainly DAPI-labelled blue nuclei (Figure S2C). After 24 h of treatment, a large number of TUNEL-positive nuclei appeared in the mycelial cells, indicating that the DNA had been fragmented (Figure S2D).

### 3.2. Transcriptome Changes after H_2_O_2_ Treatment

The global DEGs in *L. edodes* mycelia after 0, 3, and 24 h of 100 μM H_2_O_2_ treatment were determined via high-throughput sequencing. A total of 384 Mb clean reads with Q20 values greater than 98% were obtained, and the average total reads mapped were 89.26% (Tables S1 and S2). The lowest Pearson correlation coefficient between biological replicates was 0.9856, indicating the reliability and validity of the RNA-seq data (Figure S3). Three DEG categories of LeHH3h vs. LeHH0h, LeHH24h vs. LeHH0h, and LeHH24h vs. LeHH3 were compared to understand the mechanism by which H_2_O_2_ affected the mycelia. In total, 2398 DEGs were identified in the three categories, accounting for 23.0% of all transcripts. Compared with LeHH0h, LeHH3h had 351 upregulated genes and 194 downregulated genes (Table S3). LeHH24h had 1032 DEGs (619 upregulated and 413 downregulated) compared with LeHH0h and 821 DEGs (456 upregulated and 365 downregulated) compared with LeHH3h (Tables S4 and S5).

The Venn diagram showed that 83 genes were common among the three categories (Figure S4 and Table S6). The numbers of DEGs uniquely expressed in LeHH3h vs. LeHH0h, LeHH24h vs. LeHH0h, and LeHH24h vs. LeHH3h were 154, 271, and 182, respectively. Cluster analysis was performed to divide the DEGs into three main clusters and six subclusters according to the duration of H_2_O_2_ treatment (Figure S5). The genes in cluster 1 were downregulated after H_2_O_2_ treatment, whereas the main genes in cluster 3 were upregulated after H_2_O_2_ treatment. In addition, the genes in cluster 2 were first downregulated after 3 h and then upregulated after 24 h of treatment. Moreover, the sample of LeHH3h clustered with that of LeHH0h, highlighting the similarity between the two samples. 

The DEGs in LeHH24h vs. LeHH0h were enriched to analyse their functional features. The top 20 enriched GO terms included cell wall, nucleosome, ribosome, chromatin, mitochondrial inner membrane, and mitochondrial envelope, which belonged to 16 cellular component categories (Figure S6 and Table S7). Besides cellular components, the GO terms were also enriched in oxidation–reduction, structural constituent of cell wall, and structural molecule activity, which belonged to biological process and molecular function. Furthermore, the DEGs were mapped to the KEGG pathway to identify the active metabolism pathways involved in H_2_O_2_ response. The top 20 enriched KEGG terms included ribosome, glycolysis/gluconeogenesis, tyrosine metabolism, glyoxylate and dicarboxylate, oxidative phosphorylation, glycerolipid metabolism, fatty acid degradation, and mitophagy (Figure S7 and Table S8). 

### 3.3. Expression Changes of the Mitophagic Pathway Genes in Mycelia under H_2_O_2_ Treatment

**Figure 2:**
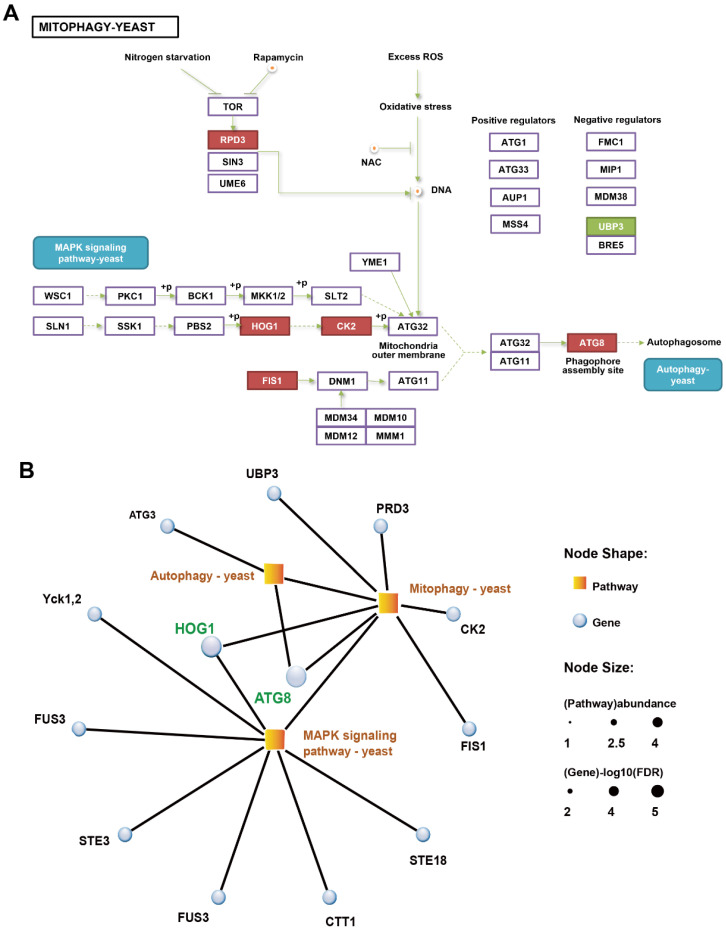
DEGs of LeHH24h vs. LeHH0h enriched in the mitophagic pathway and their interaction networks. (**A**) Mitophagy map of enriched DEGs in LeHH24h vs. LeHH0h; red box indicates upregulated gene. (**B**) Interaction networks of the autophagic, mitophagic, and MAPK signal pathways analysed using the enriched DEGs in LeHH24h vs. LeHH0h.

Among the enriched KEGG pathways, six mitophagy-related genes were noted, five of which *(LeAtg8*, *LeHog1*, *LeRpd3*, *LeCk2*, and *LeFis1*) were upregulated, while one negative regulator gene (*LeUbp3*) was downregulated after H_2_O_2_ treatment (Figure 2A). The expression levels of *LeAtg8* and *LeHog1* verified using qRT-PCR assays were consistent with the RNA-seq results (Figure S8). LeATG8 (Led00777, fold change = 1.95) is a ScATG8 homologue, which is a core autophagic machinery component that promotes phagophore elongation [23]. LeHOG1 (Led03258, fold change = 1.75) encodes a homologue of a high-osmolarity glycerol protein (ScHOG1), which is an upstream regulator of the MAPK signal pathway for eukaryotic cells [24]. HOG1–MAPK (HOG) pathways are required for selective autophagy [25]. Mitochondrial fission protein 1 (LeFIS1, Led09311, fold change = 2) is essential in mitochondrial division/fission and apoptotic and mitophagic pathways [26]. Small nuclear ribonucleoprotein Sm-D3 (LeRPD3, Led05652 fold change = 1.63) controls RNA splicing and plays a role in DNA double-strand break repair [27,28]. Casein kinase 2 (LeCK2, Led08009, fold change = 1.36) is involved in multiple cellular functions, including ATG32 phosphorylation in mitophagy [29]. Deubiquitinase Ubp3 (LeUBP3, Led00043, fold change = 0.57), together with its co-factor Bre5, is a negative regulator of mitophagy [30].

The KEGG pathway enrichment network of autophagic, mitophagic, and MAPK signal pathways was visualised to understand the relationship among these pathways and identify interactive hub genes (Figure 2B). The MAPK signal pathway had the greatest number of genes (*n* = 7), followed by the mitophagy pathway (*n* = 6) and the autophagy pathway (*n* = 2). Hub genes *LeAtg8* and *LeHog1* were involved in two of the three pathways. *LeAtg8* was mapped on the autophagic and mitophagy pathways, whereas *LeHog1* was mapped on the mitophagy and MAPK signal pathways. Considering the possible important roles of these genes in H_2_O_2_-induced autophagy, we focused on them in subsequent analyses.

### 3.4. LeHOG1 Phosphorylation Participated in LeATG8-Dependent Autophagy Induced by H_2_O_2_

**Figure 3:**
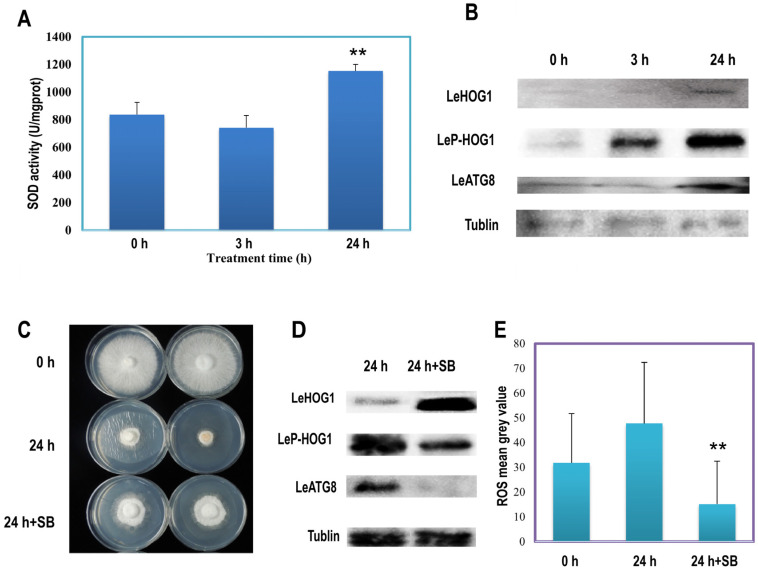
Related protein variation after induction and inhibition of oxidative stress in *Lentinula edodes* mycelia. (**A**) Superoxide dismutase (SOD) activity of mycelia treated with H_2_O_2_. Mean ± SD, *n* = 4. ** *p* < 0.01 vs. 0 h. (**B**) Western blots of LeHOG1 phosphorylation and LeATG8 expression under oxidative stress. (**C**) Mycelial morphology after 24 h treatment with H_2_O_2_ or SB203580 inhibitor and H_2_O_2_. (**D**) Western blots of LeHOG1 phosphorylation and LeATG8 expression after 24 h treatment with H_2_O_2_ or SB203580 inhibitor and H_2_O_2_. (**E**) Variation of ROS content in CK, induction, and inhibition of oxidative stress. Mean ± SD, *n* = 20. ** *p* < 0.01 vs. 24 h.

H_2_O_2_ induces oxidative stress by exerting cytotoxic effects and increasing intracellular superoxide (O_2_·¯) levels [31]. In the present study, treatment with H_2_O_2_ for 24 h significantly increased the antioxidant activity of superoxide dismutase (SOD) activity (Figure 3A). Moreover, DCFH-DA dyeing results showed that the treatment increased the ROS content of the mycelia (Figure S9). The high-osmolarity glycol (HOG) pathway may be associated with oxidative stress. Hog1 is activated by dual phosphorylation on a tripeptide motif (Thr-X-Tyr) via promoting high levels of ROS [32]. Therefore, Western blotting was used to verify the expression levels of LeATG8 and LeHOG1 and the expression level of phospho-LeHOG1 in H_2_O_2_-induced ageing mycelia. The expression of LeATG8 and LeHOG1 showed no change in the mycelia after 3 h of H_2_O_2_ treatment compared with the control (Figure 3B) but was upregulated after 24 h of treatment, indicating that autophagy was induced by H_2_O_2_. Meanwhile, the phosphorylation level of LeHOG1 increased as early as 3 h and continued to increase until 24 h (Figure 3B). This immediate and long-lasting phosphorylation of LeHOG1 suggested that LeHOG1 played a crucial part in response to oxidative stress and the activation of LeATG8-dependent autophagy. Furthermore, we used the HOG1 inhibitor SB203580 to inactivate LeHOG1 selectively. SB203580 (10 μM)-pretreated cellophane and untreated cellophane were used for mycelial culture, which was then treated with 100 μM H_2_O_2_ and incubated for 24 h. SB203580 slightly relieved the growth inhibition caused by exogenous H_2_O_2_ treatment (Figure 3C). At the protein level, SB203580 reduced LeHOG1 phosphorylation and LeATG8 expression (Figure 3D). However, LeHOG1 expression was increased. According to statistical analysis, on adding the SB203580 inhibitor, the intracellular ROS was significantly reduced compared with the H_2_O_2_ treatment group (Figure 3E and Figure S9). Inactivation of LeHOG1 might inhibit the function of LeATG8, demonstrating that the LeATG8-dependent autophagy in *L. edodes* was strongly correlated with LeHOG1 MAPK activity or even phosphorylation. 

### 3.5. Subcellular Localisation of LeATG8, LeHOG1, and Phospho-LeHOG1 during H_2_O_2_ Treatment

**Figure 4:**
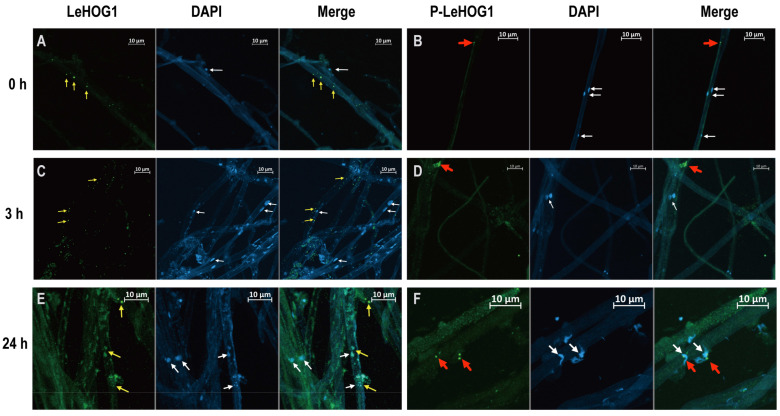
Subcellular localisation of LeHOG1 and phospho-LeHOG1 in mycelia treated with 100 μM H_2_O_2_. (**A**,**C**,**E**) Immunofluorescence of LeHOG1-labelled (green) and DAPI-stained (blue) mycelia after 0, 3, and 24 h of treatment. (**B**,**D**,**F**) Immunofluorescence of phospho-LeHOG1-labelled (green) and DAPI-stained (blue) mycelia after 0, 3, and 24 h of treatment. Yellow arrows indicate LeHOG1-labelled mycelia. Red arrows indicate phospho-LeHOG-labelled mycelia. White arrows indicate nuclei.

**Figure 5:**
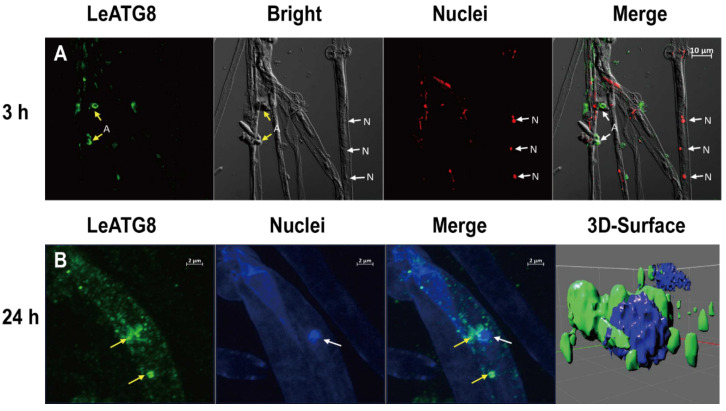
Subcellular localisation of LeATG8 in the mycelia treated with 100 μM H_2_O_2_. (**A**) Immunofluorescence of LeATG8-labelled (green) and DAPI-stained (blue) mycelia after 3 h treatment. (**B**) Immunofluorescence of LeATG8-labelled (green) and DAPI-stained (blue) mycelia after 24 h treatment. Yellow arrows indicate LeATG8-labelled mycelia. White arrows indicate nuclei. A, autophagosome; N, nuclei.

We evaluated the cellular localisations of LeATG8, LeHOG1, and phospho-LeHOG1 using immunofluorescence. We labelled these proteins using the same antibodies used in the Western blot analysis and evaluated the nuclear localisation using DAPI. Without H_2_O_2_ treatment, LeATG8, LeHOG1, and phospho-LeHOG1 localised in the cytoplasm as control (Figure 4A,B and Figure S10A). After a 3 h treatment, they were still located in the cytoplasm, while the phospho-LeHOG1 in the cytoplasm aggregated (Figure 4C,D and Figure S10B). After 24 h of H_2_O_2_ treatment, the positive fluorescence of LeAtg8, LeHOG1, and phospho-LeHOG1 was stronger than that after 3 h H_2_O_2_ and control treatments, which agreed with the Western blot results (Figure 4E,F and Figure S10C). In nearly 90% of visual fields, LeHOG1 and phospho-HOG1 were clearly located in the nucleus. This result suggested that, upon exposure to H_2_O_2_, phospho-LeHOG1 was translocated into the nucleus, where it directly targeted several transcription factors, as observed in yeasts under hyperosmotic stress [33]. Additionally, in nearly 60% of visual fields, we clearly observed a ring-shaped autophagosome structure in *L. edodes* hyphae by labelling the LeATG8 protein (Figure 5A), and a 3D reconstruction of the Z-stack images of immunofluorescence staining was obtained (Figure 5B). The results of 3D imaging clearly showed that LeAtg8-labelled autophagosomes (green) were in the process of enveloping the nucleus (blue). 

## 4. Discussion

ROS are direct triggers of oxidative stress [34] generated by the mitochondria, which are the main inducers of autophagy under oxidative stress [35]. ROS-induced damage of cellular constituents leads to mitochondrial dysfunction, DNA damage, chromatid breaks and mutations, and reduced metabolic efficiency [36]. The accumulation of damaged mitochondria in the cell can cause cellular oxidative stress and eventually lead to cell death [37]. When *L. edodes* mycelia were grown in the presence of H_2_O_2_, ROS blocked mycelial growth due to cellular damage in the early stage. After 20 days of treatment, mycelia agglomerated to form knoblike protuberances, which are similar to mycelial knots in spawn bags (Figure 1). In *L. edodes* cultivation, mycelia transform from vegetative growth to reproductive growth after mycelial maturation. Mycelial knots are the precursor of a primordium or fruiting body initiation, and mycelial knot formation in spawn bags usually takes 60 days. In the present study, H_2_O_2_ treatment caused the premature formation of knoblike protuberances, suggesting that H_2_O_2_ accelerated the ageing of *L. edodes* mycelia. Moreover, ROS accumulation decreases MMP and triggers apoptosis [36,38,39]. In *L. edodes*, the ratio of JC-1 aggregates to JC-1 monomers (red/green) gradually reduced, indicating mitochondrial depolarisation of mycelia treated with H_2_O_2_ (Figure S1). Moreover, the nucleus in the mycelium also became TUNEL positive after 100 μM H_2_O_2_ treatment (Figure S2). Antioxidant enzymes, such as SOD, could metabolise ROS to increase resistance to oxidative stress [40].

This phenomenon was confirmed by the significantly enhanced SOD activity and ROS content in the H_2_O_2_-treated *L. edodes* mycelia (Figure 3). In our previous study, the expression of autophagy key protein LeATG8 was significantly increased in long-term cultured mycelium. The later stage of culture showed that the degree of autophagy was deepened, with similar changes of cell structure and SOD activity to those after 24 h H_2_O_2_ treatment. Our findings indicated that 24 h H_2_O_2_ treatment induced rapid artificial ageing of *L. edodes* mycelia. The expression of LeATG8 protein in the mycelium significantly increased after 24 h treatment, indicating that this model can be used as an induction model for studying autophagy in *L. edodes*.

Damaged cellular constituents and organelles must be selectively removed by autophagy to protect cells from excessive oxidative stress and cell death [37]. A possible relationship between autophagy and mycelial ageing in *L. edodes* has been previously suggested [18]. However, data on the determination of autophagosomes in *L. edodes* remain limited. As Atg8 has been used as a protein marker of the double-layer membrane autophagosome in yeast and mammalian cells, in the present study, using fluorescent labelling, we observed the classic ring structure of autophagosomes in *L. edodes* hyphae for the first time (Figure 5). Moreover, 3D imaging revealed that LeATG8 was enriched on the nuclear periphery (Figure 5), suggesting that autophagosomes surrounded these nuclei to degrade them at specific stages. This result provided strong evidence for the importance of LeATG8 in nuclear degradation. Meanwhile, LeATG3, which encodes a homologue of an E2-conjugating enzyme that generates ATG8–PE, was also identified via the enrichment network of the ageing mycelia (Figure 2). These results implied the formation of autophagosomes and the occurrence of autophagy in the ROS-induced mycelia.

When the selective cargo is the mitochondria, autophagy becomes mitophagy [41,42]. In the present study, exogenous H_2_O_2_ treatment increased ROS production in *L. edodes* mycelia, which resulted in MMP loss, oxidative stress, and ageing (Figure 1, Figure 3, Figures S1 and S8). MMP loss is obvious because ROS-induced mitophagy requires mitochondrial depolarisation [37]. Furthermore, mitophagy-related genes (*LeAtg8*, *LeHog1*, *LeRpd3*, *LeCk2*, *LeFis1,* and *LeUbp3*) were overexpressed in ageing mycelia, and an enrichment network of autophagic, mitophagic, and MAPK signal pathways was constructed (Figure 2). ATG8 is essential in all autophagic pathways, including mitophagy. Therefore, LeATG8 in the present study acted as a hub gene involved in autophagy and mitophagy. In addition, mitophagy is specifically mediated by ATG32, which is anchored in the mitochondrial outer membrane, acting as a receptor for ATG8 to recruit the autophagy machinery to the mitochondrial surface [41]. ATG32 interaction with scaffolding component ATG11 marks mitochondrial degradation [25,43]. When ATG11 engages the mitochondria with ATG8–PE, the Fis1–Mdv1–Dnm1 molecular complex is necessary for mitochondrial fission and fragmentation [25]. The high expression of LeFIS1 in the present study confirmed that the mycelia with ROS-induced ageing underwent mitophagy (Figure 2).

Aside from the autophagy machinery, upstream signalling regulation is also indispensable for mitophagy. Rapamycin complex 1 (TORC1) in yeast is a negative regulator of autophagy and mitophagy [41]. Under mitophagy-inducing conditions, TORC1 is suppressed, and its downstream Ume6–Sin3–Rpd3 complex releases ATG32 transcription repression, resulting in ATG32 expression [43]. LeRPD3, a homologue of Rpd3 of histone deacetylase complex in *L. edodes*, was 1.63-fold overexpressed in the present study (Figure 2), indicating the regulation of mitophagy through protein kinase TORC1. Similarly, it was also previously suggested that LeRPD3 and LeTORC1 are involved in oxidative stress and autophagy of the *L. edodes* brown film formation process through proteomic quantification analysis [15]. Different from TOR signalling, the MAPK signalling pathway regulates mitophagy but not non-selective autophagy [41]. The ATG32/ATG11 interaction is strictly regulated via ATG32 phosphorylation through kinase CK2 [25,43]. During mitophagy, LeCK2 expression showed a 1.36-fold upregulation in the ROS-induced ageing mycelia of *L. edodes*, suggesting the active phosphorylation of LeATG32 by LeCK2 (Figure 2). Activation of CK2 depends on the MAPKs of the HOG pathway [41].

In yeast and other filamentous fungi (e.g., *Aspergillus*), HOG1 acts as a kinase in the MAPK cascade, and high intracellular concentrations of H_2_O_2_ can activate HOG1 [44,45]. Transcriptome, RT-qPCR, and Western blot analyses showed that H_2_O_2_ treatment increased the relative expression level of LeHOG1 and regulated the downstream mitophagic pathway (Figure 2, Figure 3 and Figure S7). Based on antioxidant response, H_2_O_2_ induces major changes in protein phosphorylation [46]. Cytoplasmic HOG1 is phosphorylated by dual specific kinase PBS2 and subsequently translocates to the nucleus and activates the downstream transcription factor, thereby altering gene expression under stress [24]. In the present study, H_2_O_2_ treatment increased the phosphorylation of LeHOG1 as early as 3 h and lasted into 24 h, whereas the protein level of non-phosphorylated LeHOG1 increased until 24 h treatment (Figure 3). Phospho-LeHOG1 agglutinated after 3 h of treatment and then translocated into the nucleus after 24 h, with the time course matching its phosphorylation status (Figure 4). LeHOG1 also translocated to the nucleus following H_2_O_2_ treatment. Similarly, in fungi (e.g., *Saccharomyces cerevisiae, Alternaria,* and *Magnaporthe*), the threonine (Thr) and tyrosine (Tyr) residues on the Hog1 protein are double phosphorylated under the action of ROS. LeHOG1 and phospho-LeHOG1 transfer to the nucleus to regulate gene transcription and increase cell resistance to oxidative stress and autophagy [33,44,46,47,48]. Additionally, the HOG1 inhibitor SB203580 blocked LeATG8 and phospho-LeHOG1, which consequently upregulated LeHOG1 expression (Figure 3). This result suggests a link between the LeATG8-dependent autophagy in *L. edodes* mycelial and LeHOG1 phosphorylation. While inhibiting HOG1 phosphorylation, SB203580 also reduced intracellular ROS content. Similar results that the inhibition of P38 (HOG1 homologous protein in mammals) phosphorylation can inhibit ROS content in human or animal cells have been reported [49,50,51]. However, in addition to the HOG–MAPK pathway, there are many ways to respond to oxidative stress in fungi. In this study, we also found that the transcription of catalase (CTT1) was upregulated (Figure 2B). Therefore, we suggested that inhibition of autophagy and increase in the protein expression of non-phosphorylated LeHOG1 triggered other antioxidant responses. Thus, the ROS content in the hyphal cells was reduced, which increased the survival of the cells under oxidative stress.

## 5. Conclusions

In the present study, we successfully induced autophagy in *L. edodes* mycelia through exogenous H_2_O_2_ application. We comprehensively studied the expression and subcellular localisation of ROS and autophagy-related proteins during H_2_O_2_ treatment of mycelia. Using fluorescent labelling, we observed, to the best of our knowledge, for the first time, the classic ring structure of autophagosomes in *L. edodes* hyphae. Moreover, 3D imaging showed that LeATG8 was enriched on the nuclear periphery, suggesting that autophagosomes surrounded these nuclei to degrade them at specific stages. Our results indicated that both LeATG8 and LeHOG1 are involved in the mycelial autophagy of *L. edodes*. LeATG8 expression could be blocked by inhibiting LeHOG1 phosphorylation, which suggested a relationship between autophagy and oxidative stress in *L. edodes*. Further, in-depth exploration of the interaction between LeHOG1 and LeATG8, as well as the correlation between MAPK and autophagy pathways, will provide deeper insights into ROS-induced autophagy involvement in the ageing or pigmentation of *L. edodes*.

## Data Availability

All data generated or analysed during this study are included in this published article and its supplementary information files. The raw sequencing data of RNA-seq are available at the National Genomics Data Center, China National Center for Bioinformation, under BioProject ID PRJCA012101 (https://ngdc.cncb.ac.cn/bioproject/browse/PRJCA012101, accessed on 17 March 2023). The LeHH0h sample was labelled as LeC1.

## Supplementary Materials

The following supporting information can be downloaded at https://www.mdpi.com/article/10.3390/jof9040413/s1: Figure S1: JC-1 staining of mycelia treated with 0, 8, or 100 μM H_2_O_2_. Figure S2: Subcellular phenotype changes of *Lentinula edodes* mycelia after treatment with 100 μM H_2_O_2_ for 3 h and 24 h. Figure S3: Pearson correlation value between biological replicates. Figure S4: Venn diagram of DEGs in LeHH3h vs. LeHH0h, LeHH24h vs. LeHH0h, and LeHH24h vs. LeHH3. Figure S5: Hierarchical clustering of all DEGs. Figure S6: GO enrichment of DEGs in LeHH24h vs. LeHH0h. Figure S7: KEGG enrichment of DEGs in LeHH24h vs. LeHH0h. Figure S8: Validation of the expression of *LeAtg8* and *LeHog1* in LeHH24h vs. LeHH0h using RT-qPCR. Figure S9: DCFH-DA staining marked the reactive oxygen species (ROS) content of hyphae under control, 24 h H_2_O_2_ treatment, and 24 h H_2_O_2_ inhibition with SB203580. Figure S10: Subcellular localisation of LeATG8 in mycelia treated with 100 μM H_2_O_2._ Table S1: Data summary of RNA-seq. Table S2: Summary of clean reads mapped to reference genome. Table S3: DEG lists of LeHH3h vs. LeHH0h. Table S4: DEG lists of LeHH24h vs. LeHH0h. Table S5: DEG lists of LeHH24h vs. LeHH3h. Table S6: Common DEGs between Venn diagram. Table S7: The top 20 enriched GO terms of DEGs in LeHH24h vs. LeHH0h. Table S8: The top 20 enriched KEGG terms of DEGs in LeHH24h vs. LeHH0h.
