# Supplementary material for: HOG1 Mitogen-Activated Protein Kinase Pathway–Related Autophagy Induced by H2O2 in Lentinula edodes Mycelia
Source: J Fungi (Basel). 2023 Mar 28;9(4):413. doi: 10.3390/jof9040413 (PMC10143471; doi:10.3390/jof9040413)
Supplement: Supplementary file 1 [file jof-09-00413-s001.zip › jof-2214390-supplementary.pdf]

# Supplemental Material

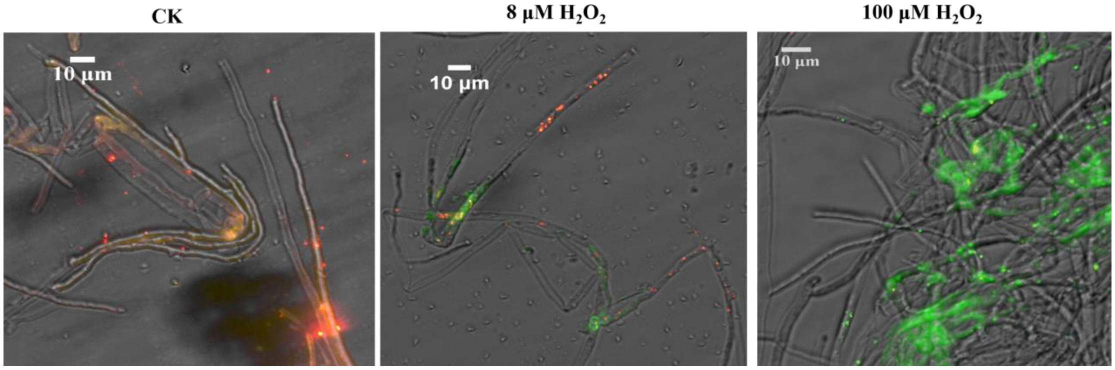


**Figure S1 JC-1 staining of mycelia treated with 0, 8, or 100 μM $H_2O_2$.**

Red fluorescence indicates polarised healthy mitochondria, and green fluorescence indicates depolarised mitochondria.

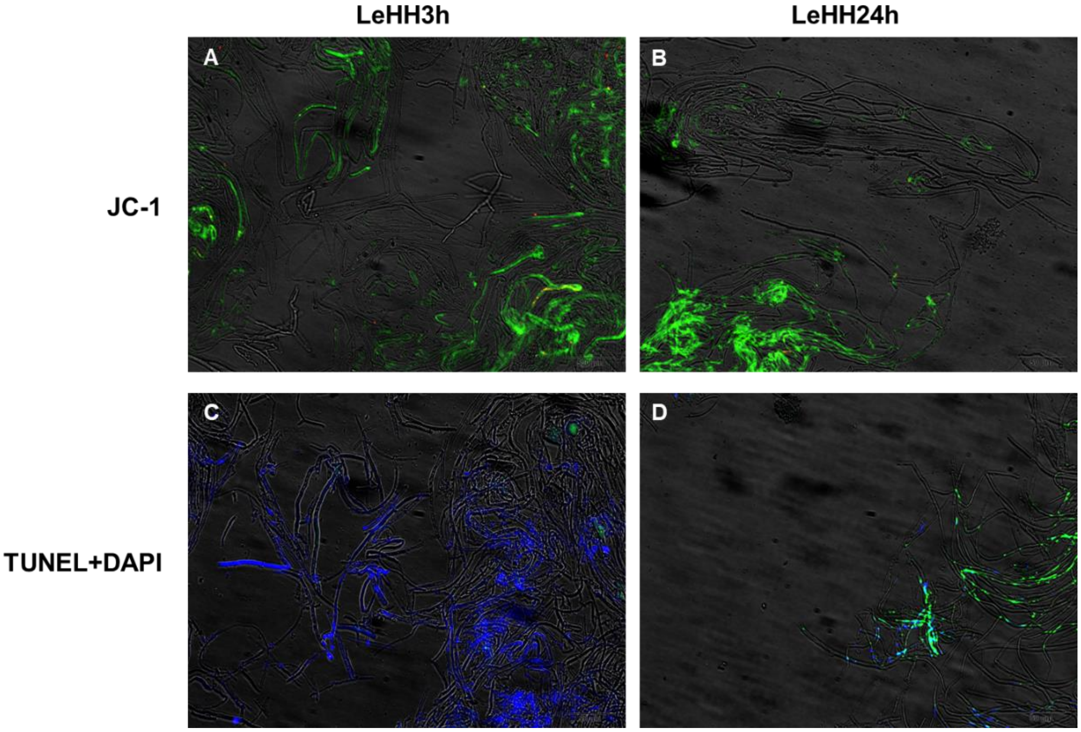


**Figue S2 Subcellular phenotype changes of *Lentinula edode*s mycelia after treated with 100 μM $H_2O_2$ for 3h and 24h.**

A-B: JC-1 labeled mitochondrial membrane potential changes in mycelia. Red fluorescence indicates polarised healthy mitochondria, and green fluorescence indicates depolarised mitochondria. C-D: The total nuclei and DNA fragments were per-formed by DAPI and TUNEL fluorescence staining. Blue fluorescence showed DAPI-labeled total nuclei. Green fluorescence showed TUNEL-labeled DNA fragments. Bar = 50 μm.

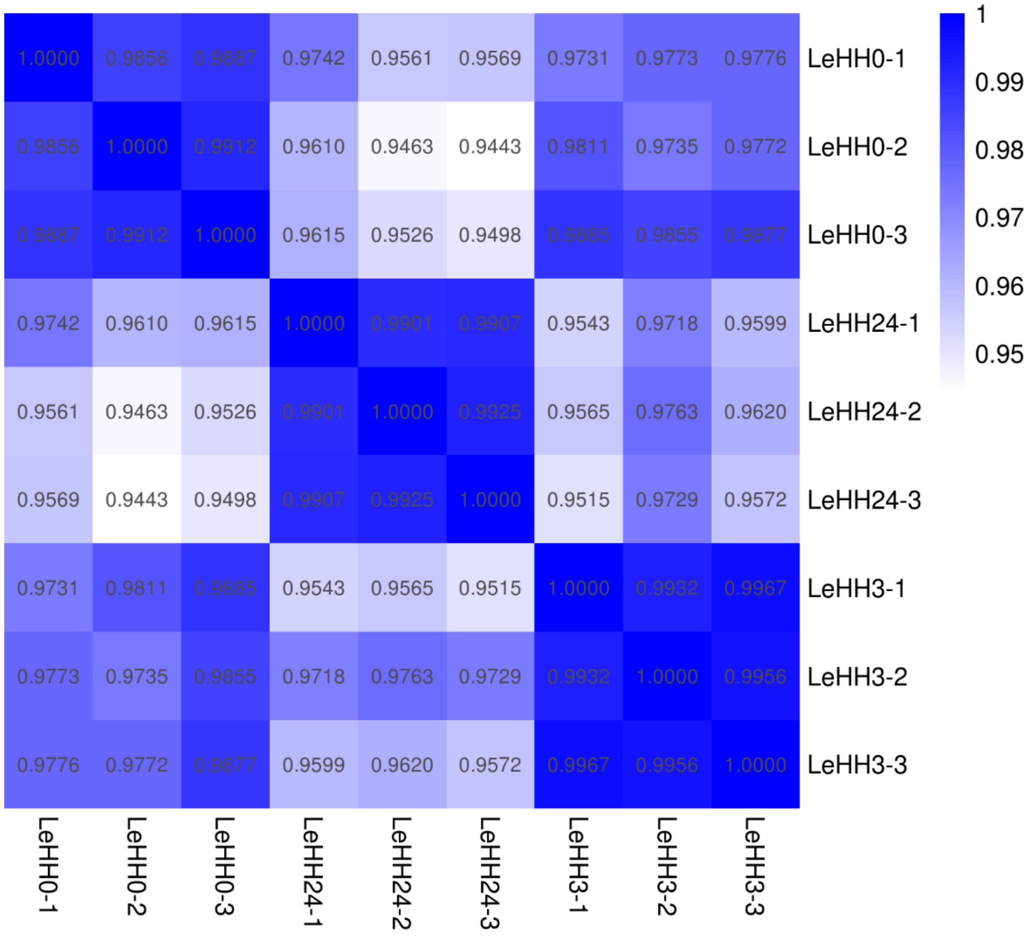


**Figure S3 Pearson correlation value between biological replicates.**

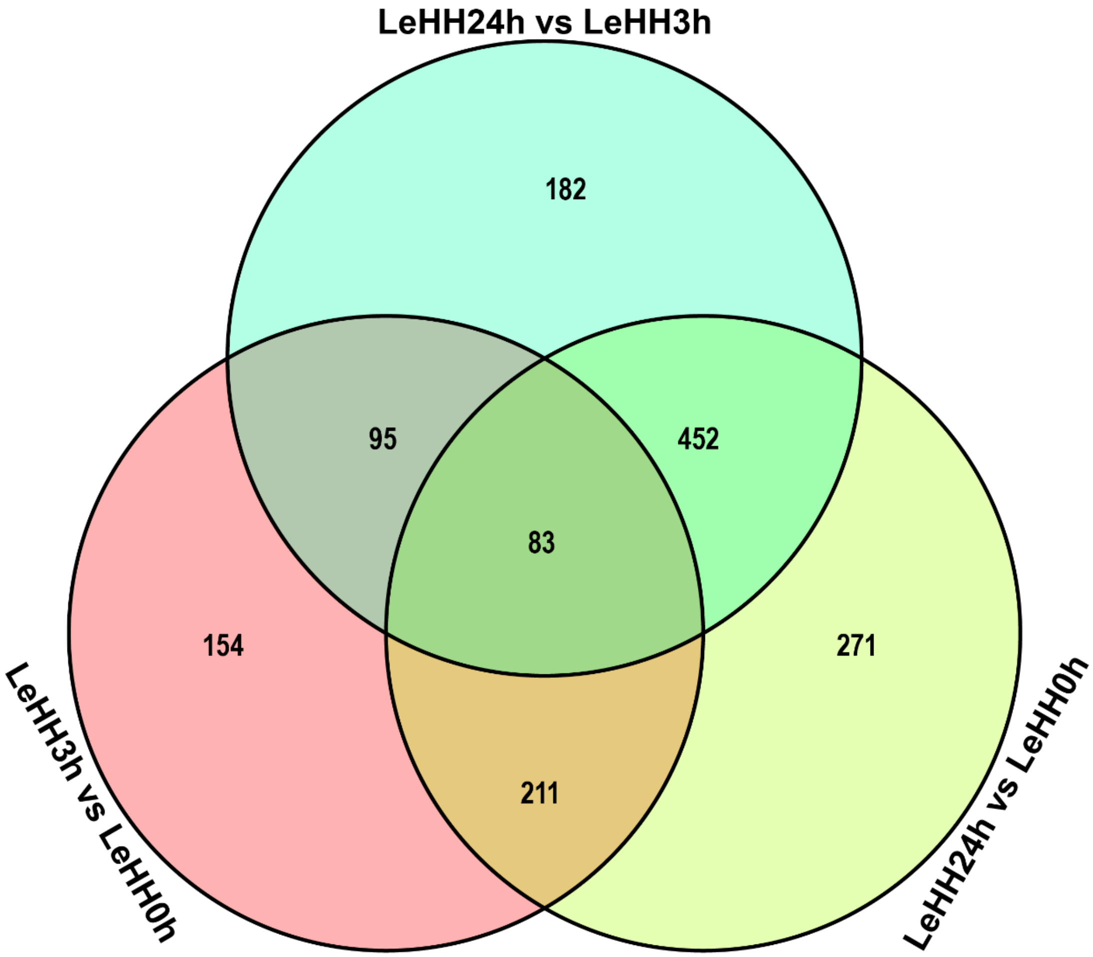


**Figure S4 Venn diagram of DEGs in LeHH3h vs. LeHH0h, LeHH24h vs. LeHH0h, and LeHH24h vs. LeHH3.**

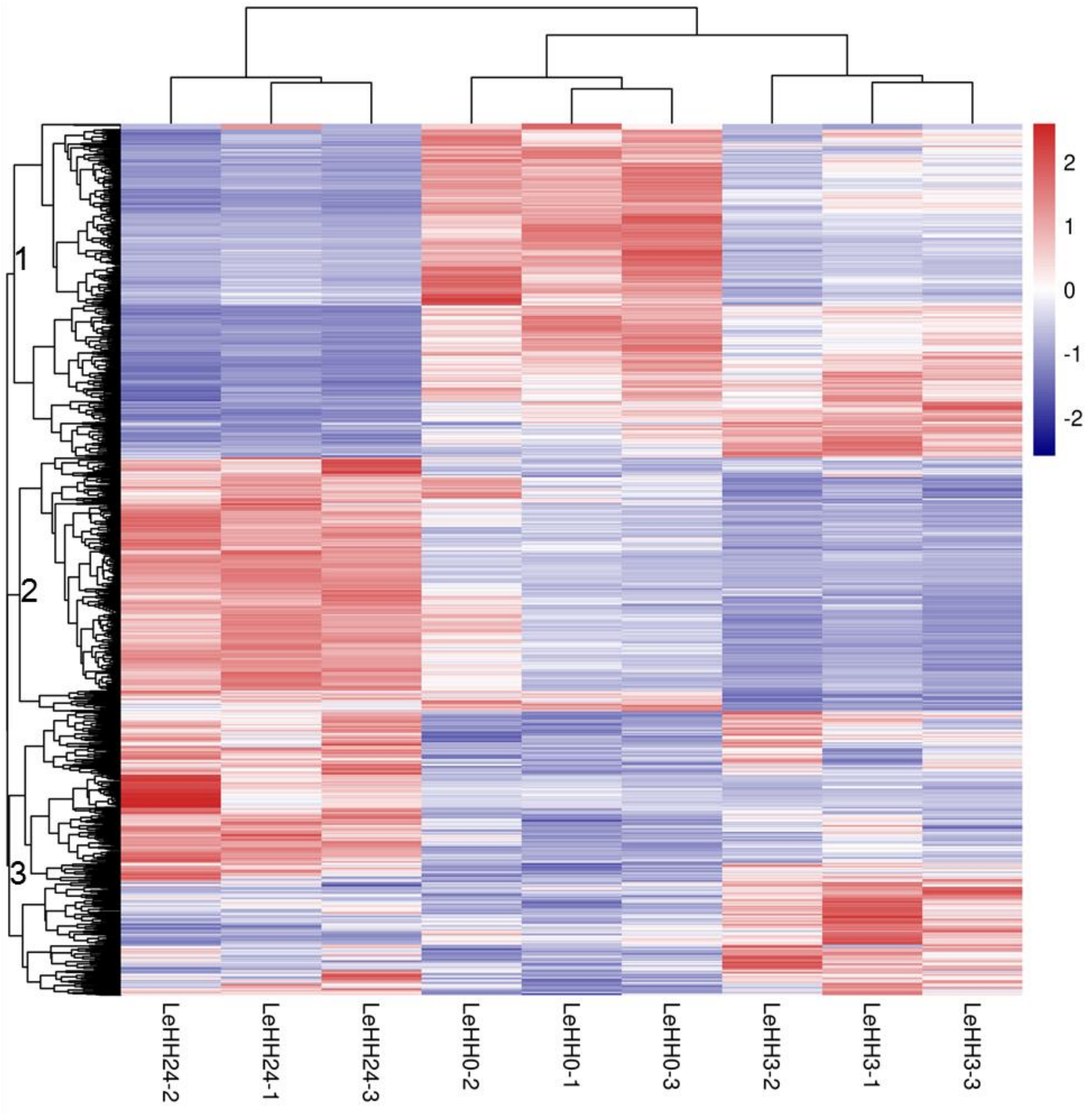


**Figure S5 Hierarchical clustering of all DEGs.** Red indicates genes with up-regulated expression, and blue indicates genes with down-regulated expression.

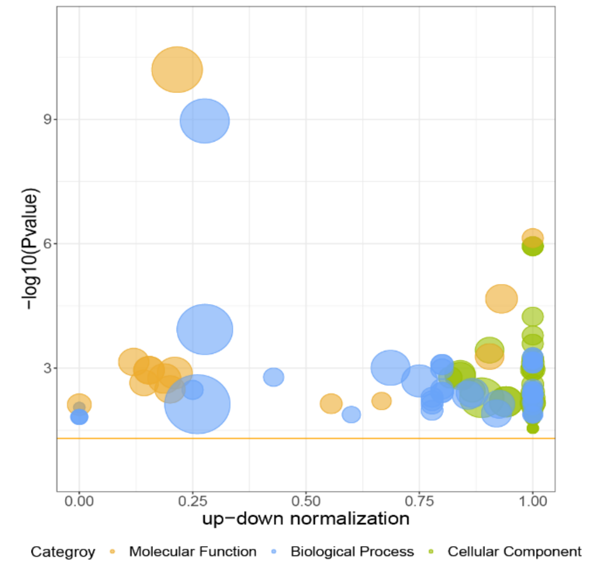


GO term Top 20

| ID | Descrption |
|---|---|
| GO:0055114 | oxidation−reduction process |
| GO:0005618 | cell wall |
| GO:0009277 | fungal−type cell wall |
| GO:0030312 | external encapsulating structure |
| GO:0000786 | nucleosome |
| GO:0032993 | protein−DNA complex |
| GO:0044815 | DNA packaging complex |
| GO:0005840 | ribosome |
| GO:0000785 | chromatin |
| GO:0005743 | mitochondrial inner membrane |
| GO:0019866 | organelle inner membrane |
| GO:0005740 | mitochondrial envelope |
| GO:0098800 | inner mitochondrial membrane protein complex |
| GO:0030529 | intracellular ribonucleoprotein complex |
| GO:1990904 | ribonucleoprotein complex |
| GO:0071944 | cell periphery |
| GO:0098798 | mitochondrial protein complex |
| GO:0016491 | oxidoreductase activity |
| GO:0005199 | structural constituent of cell wall |
| GO:0005198 | structural molecule activity |

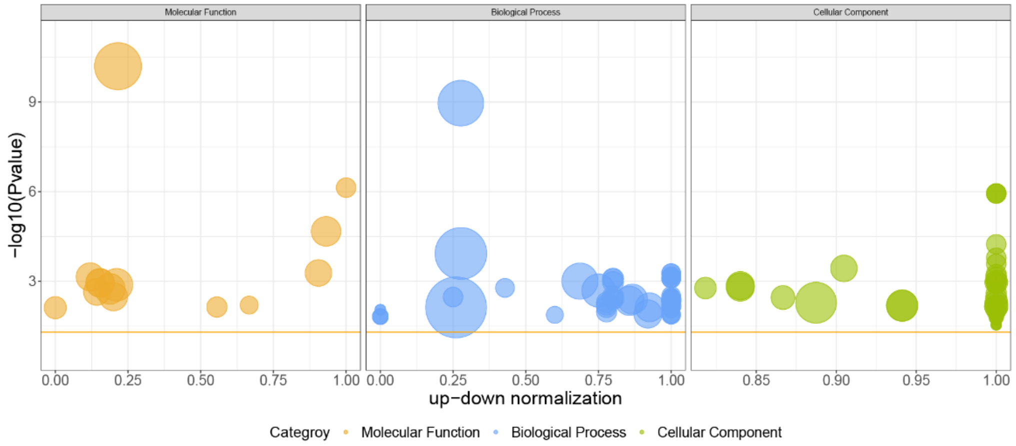


**Figure S6 GO enrichment of DEGs in LeHH24h vs. LeHH0h.**

The above figure uses two forms of bubble charts to show the results of GO enrichment analysis. The ordinate is -log10 (Pvalue). The abscissa is the up-down normalization value (the value of up-regulated gene number minus that of down-regulated gene number ratio to all DEGs). GeneCount is represented by the size of the point; the orange line in the figure represents the threshold of $p = 0.05$. The right side of a is the list of the top 20 GO terms, and different colours represent different ontologies.

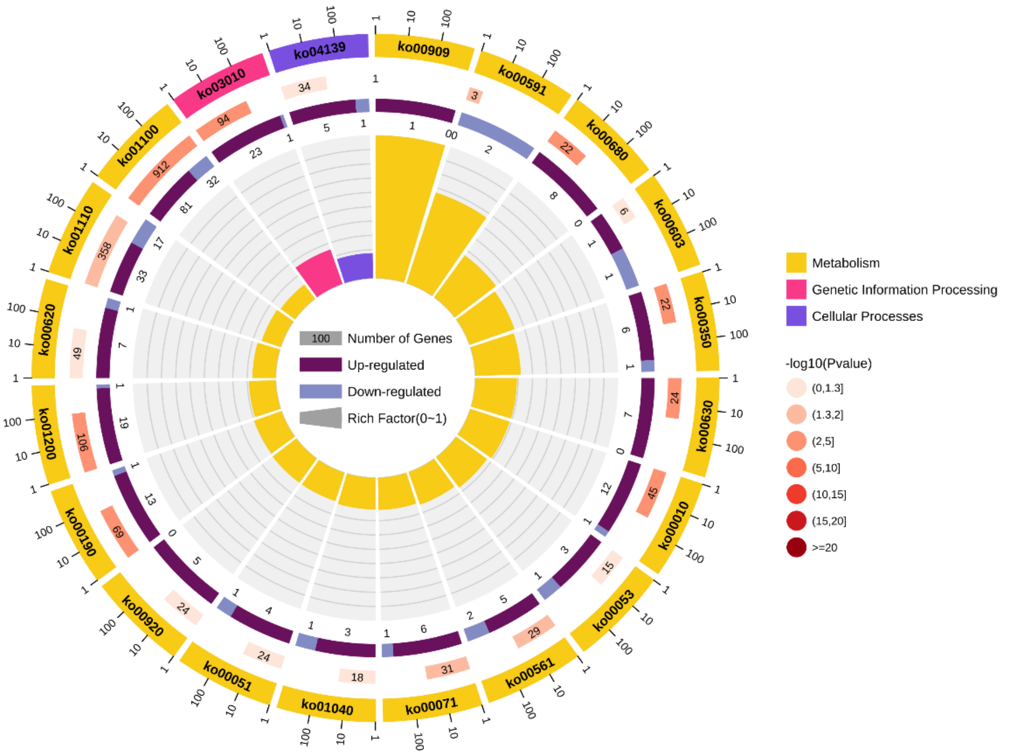


**Figure S7 KEGG enrichment of DEGs in LeHH24h vs. LeHH0h.**

Yellow label indicates metabolism class; magenta label indicates genetic information processing class; purple label indicates cellular process class. The outer ring indicates the top 20 KEGG terms, and the number of genes is indicated on the outer circle. The second ring indicates the number of the genes in the genome background and p-values for the enrichment of synteny genes for the specified biological process. The third ring indicates the number of up-regulated and down-regulated genes. Red represents the number of up-regulated genes, and blue represents the number of down-regulated genes. The fourth circle indicates the enrichment factor of each KEGG term.

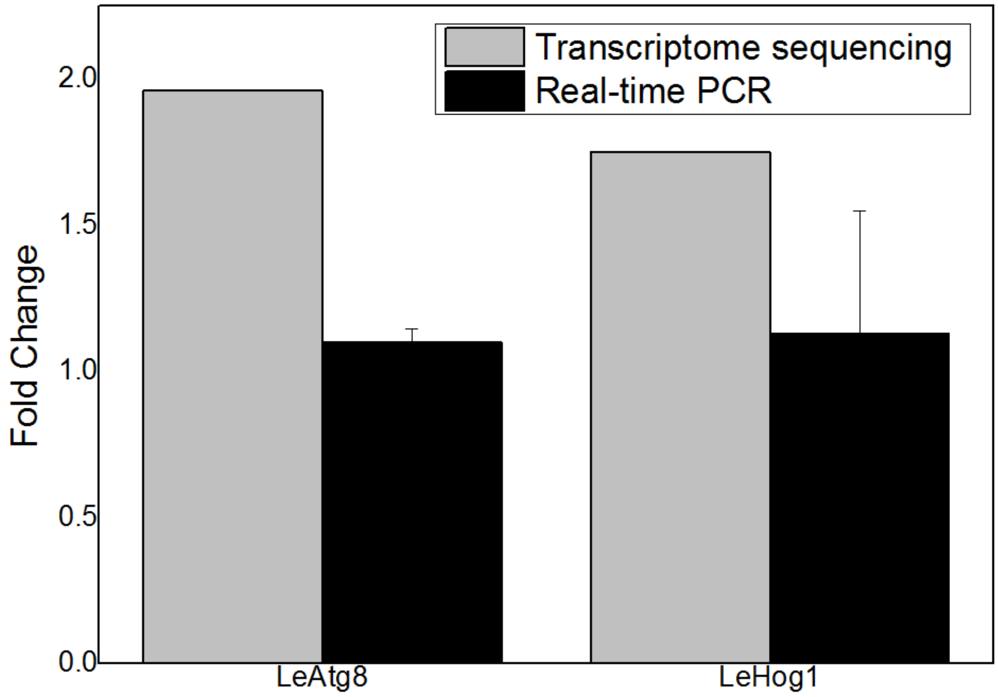


**Figure S8 Validation of the expression of *LeAtg8* and *LeHog1* in LeHH24h vs. LeHH0h using RT-qPCR.**

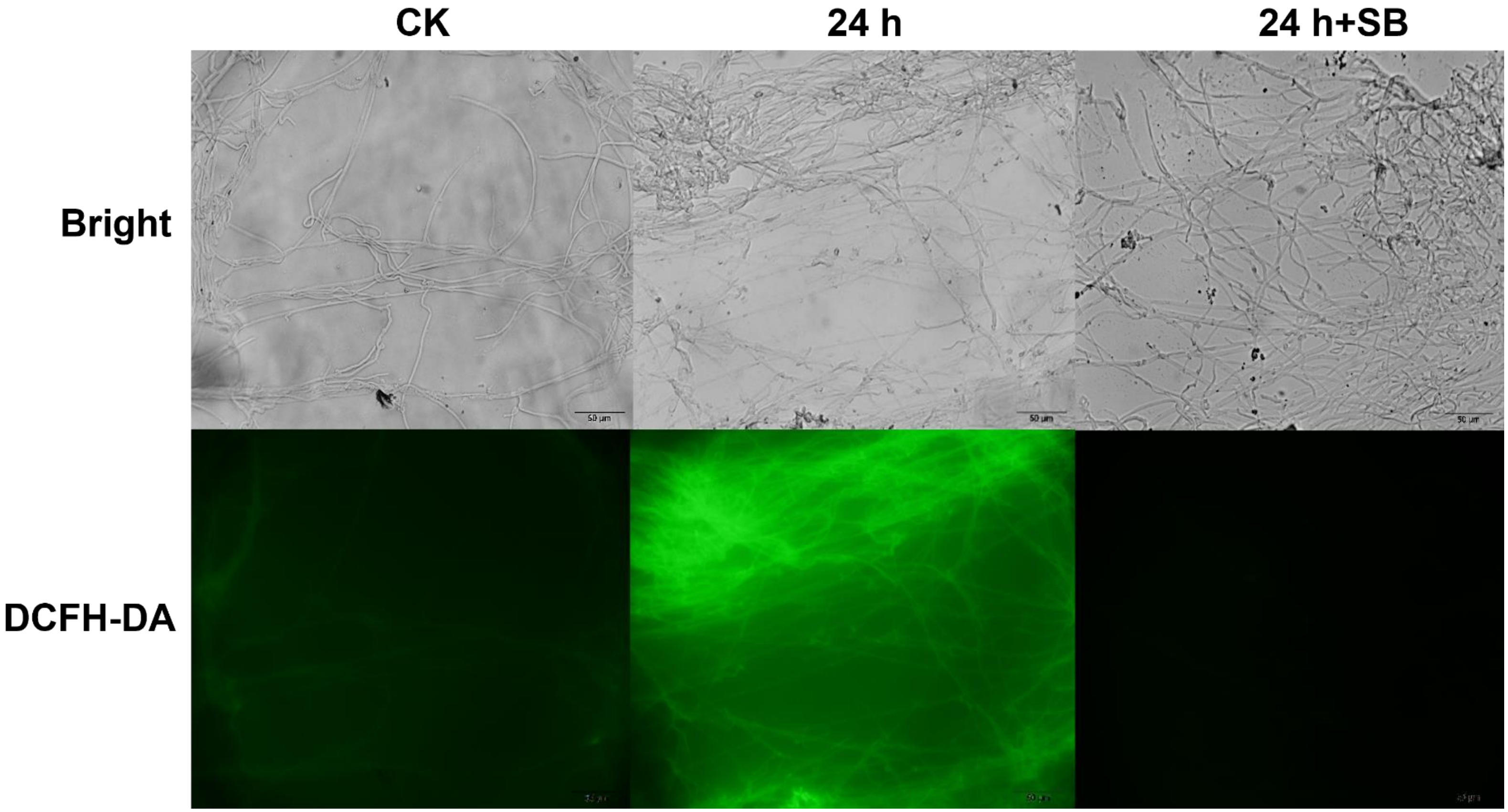


**Figure S9 DCFH-DA staining marked the reactive oxygen species (ROS) content of hyphae under control, 24 h $H_2O_2$ treatment, and 24 h $H_2O_2$ inhibition with SB203580.**

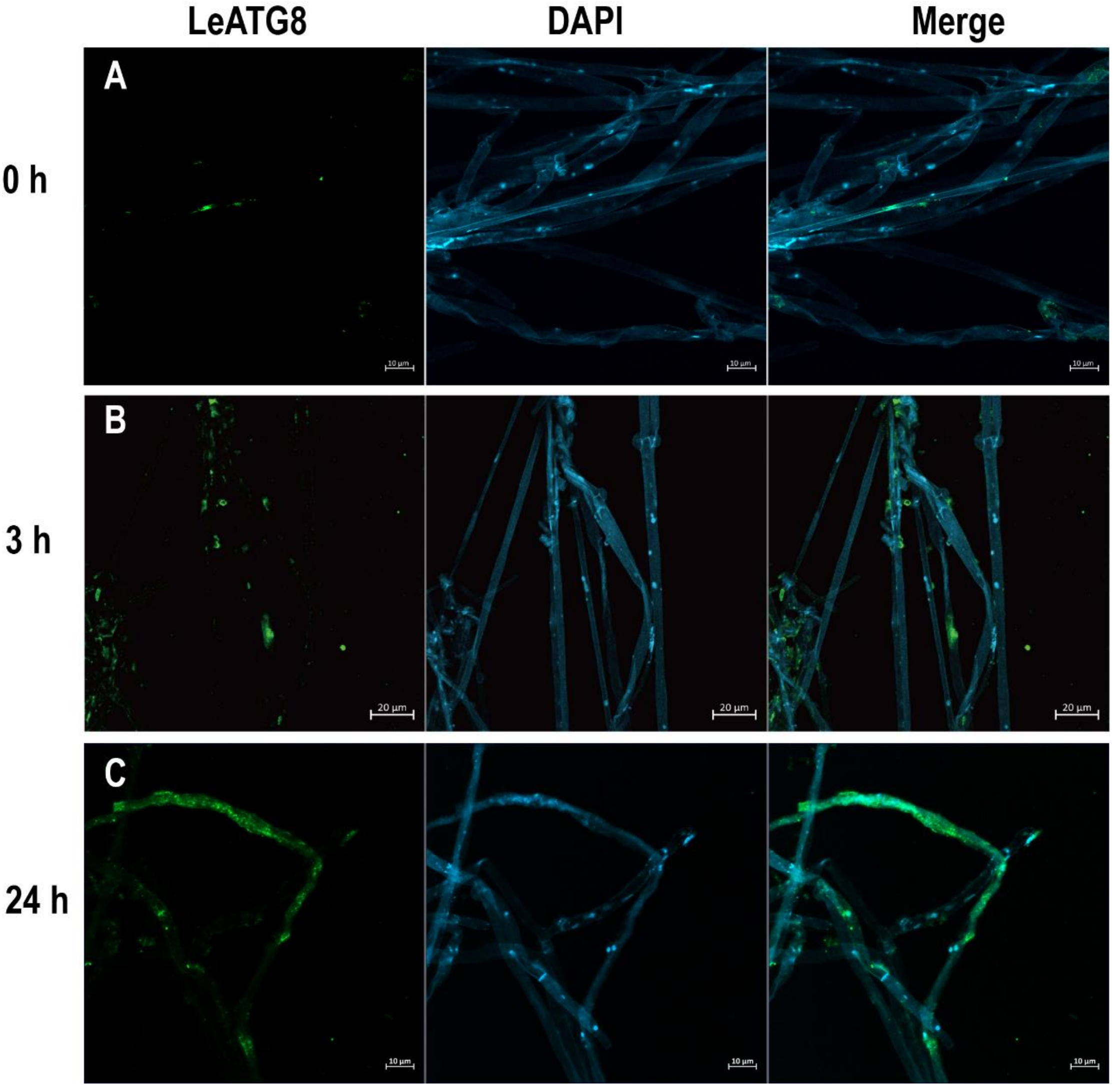


**Figure S10 Subcellular localisation of LeATG8 in mycelia treated with 100 μM $H_2O_2$.**

(A) Immunofluorescence of LeATG8-labelled (green) and DAPI-stained (blue) mycelia after 0 h treatment.

(B) Immunofluorescence of LeATG8-labelled (green) and DAPI-stained (blue) mycelia after 3 h treatment.

(C) Immunofluorescence of LeATG8-labelled (green) and DAPI-stained (blue) mycelia after 24 h treatment.

**Table S1 Data summary of RNA-seq**

| sample | raw_reads | clean_reads | clean_bases | error_rate | Q20(%)[a] | Q30(%)[b] | GC_pct |
|---|---|---|---|---|---|---|---|
| **LeC1_1** | 43100600 | 42305864 | 6.35G | 0.02 | 98.12 | 94.66 | 48.76 |
| **LeC1_2** | 46298694 | 45450024 | 6.82G | 0.02 | 98.24 | 94.92 | 48.5 |
| **LeC1_3** | 45594734 | 44563200 | 6.68G | 0.02 | 98.35 | 95.16 | 48.5 |
| **LeHH3_1** | 40431926 | 39402522 | 5.91G | 0.02 | 98.34 | 95.14 | 48.8 |
| **LeHH3_2** | 41019206 | 39876902 | 5.98G | 0.02 | 98.62 | 95.8 | 48.7 |
| **LeHH3_3** | 42045136 | 41030946 | 6.15G | 0.02 | 98.37 | 95.25 | 48.85 |
| **LeHH24_1** | 46866462 | 45408894 | 6.81G | 0.02 | 98.37 | 95.16 | 48.7 |
| **LeHH24_2** | 42659912 | 41867916 | 6.28G | 0.02 | 98.15 | 94.78 | 48.63 |
| **LeHH24_3** | 45336476 | 44450514 | 6.67G | 0.02 | 98.56 | 95.31 | 48.53 |

**Table S2 Summary of clean reads mapped to reference genome**

| Sample name | Total reads | Total mapped | Multiple mapped | Uniquely mapped | Read-1 | Read-2 |
|---|---|---|---|---|---|---|
| **LeC1_1** | 42305864 | 37620546(88.93%) | 36967948(87.38%) | 652598(1.54%) | 18584919(43.93%) | 18383029(43.45%) |
| **LeC1_2** | 45450024 | 40309563(88.69%) | 39586475(87.1%) | 723088(1.59%) | 19871732(43.72%) | 19714743(43.38%) |
| **LeC1_3** | 44563200 | 39203048(87.97%) | 38445884(86.27%) | 757164(1.7%) | 19276298(43.26%) | 19169586(43.02%) |
| **LeHH3_1** | 42740668 | 38375777(89.79%) | 37646498(88.08%) | 729279(1.71%) | 18886745(44.19%) | 18759753(43.89%) |
| **LeHH3_2** | 42238460 | 37920210(89.78%) | 37278620(88.26%) | 641590(1.52%) | 18694865(44.26%) | 18583755(44.0%) |
| **LeHH3_3** | 45925830 | 41075073(89.44%) | 40110567(87.34%) | 964506(2.1%) | 20140656(43.85%) | 19969911(43.48%) |
| **LeHH24_1** | 39402522 | 35396509(89.83%) | 34863762(88.48%) | 532747(1.35%) | 17494902(44.4%) | 17368860(44.08%) |
| **LeHH24_2** | 39876902 | 35570223(89.2%) | 34985351(87.73%) | 584872(1.47%) | 17499301(43.88%) | 17486050(43.85%) |
| **LeHH24_3** | 41030946 | 36799579(89.69%) | 36156647(88.12%) | 642932(1.57%) | 18127679(44.18%) | 18028968(43.94%) |

**Table S3 DEG lists of LeHH3h vs LeHH0h**

| gene | log2FoldChange | pvalue |
|---|---|---|
| gene-Led00038-sp3 | -0.905992319 | 0.00702816 |
| gene-Led00044-sp3 | 1.224278936 | 0.000293164 |
| gene-Led00080-sp3 | -0.84923915 | 5.40E-05 |
| gene-Led00095-sp3 | 2.885115079 | 2.55E-15 |
| gene-Led00100-sp3 | 0.554838203 | 0.004863809 |
| gene-Led00115-sp3 | -0.777072537 | 0.00044469 |
| gene-Led00127-sp3 | -1.692814351 | 2.44E-12 |
| gene-Led00165-sp3 | 0.66509999 | 0.000962843 |
| gene-Led00180-sp3 | -0.699544536 | 0.001090459 |
| gene-Led00246-sp3 | 0.361687854 | 0.006713128 |
| gene-Led00247-sp3 | -0.353592446 | 0.009184156 |
| gene-Led00250-sp3 | 0.713418516 | 0.001012739 |
| gene-Led00287-sp3 | 0.431836153 | 0.009502698 |
| gene-Led00318-sp3 | 0.908801928 | 2.26E-07 |
| gene-Led00326-sp3 | -1.527498061 | 3.85E-07 |
| gene-Led00345-sp3 | -0.835103771 | 0.000148258 |
| gene-Led00346-sp3 | 0.563827413 | 0.000527416 |
| gene-Led00428-sp3 | 0.415600444 | 0.006052967 |
| gene-Led00433-sp3 | -0.881403263 | 0.004471958 |
| gene-Led00446-sp3 | 0.501326509 | 0.000821901 |
| gene-Led00449-sp3 | 0.597080829 | 0.003630372 |
| gene-Led00458-sp3 | 0.472259709 | 0.006332523 |
| gene-Led00484-sp3 | 0.528061793 | 0.001677093 |
| gene-Led00487-sp3 | 0.569573127 | 0.000676656 |
| gene-Led00488-sp3 | 0.455115982 | 0.005897278 |
| gene-Led00503-sp3 | -0.528103383 | 0.000943759 |
| gene-Led00522-sp3 | 0.602868946 | 0.000215024 |
| gene-Led00534-sp3 | -2.770573217 | 3.48E-15 |
| gene-Led00553-sp3 | -1.964963833 | 2.84E-15 |
| gene-Led00583-sp3 | 0.492586251 | 0.008319159 |
| gene-Led00588-sp3 | -2.128996207 | 2.23E-22 |
| gene-Led00610-sp3 | 0.516385825 | 0.006181292 |
| gene-Led00622-sp3 | 1.928554104 | 0.00027588 |
| gene-Led00647-sp3 | 1.708639016 | 9.03E-12 |
| gene-Led00649-sp3 | 0.840937052 | 0.000154492 |
| gene-Led00655-sp3 | 0.673506883 | 0.001214402 |
| gene-Led00680-sp3 | -2.253411996 | 2.42E-31 |
| gene-Led00683-sp3 | -1.088912357 | 0.00018701 |
| gene-Led00702-sp3 | 0.56578008 | 0.002350954 |
| gene-Led00748-sp3 | 0.417709998 | 0.007816427 |
| gene-Led00751-sp3 | 0.411112676 | 0.008800512 |

| | | |
|---|---|---|
| gene-Led00753-sp3 | 0.579460701 | 0.001120262 |
| gene-Led00763-sp3 | 0.523424806 | 0.001615587 |
| gene-Led00803-sp3 | -0.657748087 | 0.000301205 |
| gene-Led00828-sp3 | -0.772937027 | 0.000302475 |
| gene-Led00838-sp3 | 0.497464449 | 0.007305582 |
| gene-Led00878-sp3 | -1.717705544 | 0.0000107 |
| gene-Led00899-sp3 | 0.455164045 | 0.003261332 |
| gene-Led00910-sp3 | 0.393448692 | 0.004621652 |
| gene-Led00920-sp3 | 0.588697504 | 0.003697597 |
| gene-Led00929-sp3 | -1.568631823 | 1.00E-07 |
| gene-Led00943-sp3 | -0.900984607 | 1.24E-05 |
| gene-Led00981-sp3 | -0.646324231 | 0.003171396 |
| gene-Led01058-sp3 | 1.512164869 | 0.002120197 |
| gene-Led01075-sp3 | 0.592591493 | 0.001132892 |
| gene-Led01098-sp3 | -0.65840667 | 0.000979472 |
| gene-Led01128-sp3 | 0.467760692 | 0.002954987 |
| gene-Led01148-sp3 | -2.077235426 | 0.0000312 |
| gene-Led01207-sp3 | 0.471944502 | 0.004711043 |
| gene-Led01215-sp3 | 0.512411991 | 0.001813607 |
| gene-Led01218-sp3 | 1.706177399 | 1.96E-15 |
| gene-Led01260-sp3 | 0.71147662 | 0.001290371 |
| gene-Led01274-sp3 | -0.545757821 | 0.000830247 |
| gene-Led01295-sp3 | 0.515742065 | 0.008793046 |
| gene-Led01321-sp3 | -0.716905089 | 0.00881039 |
| gene-Led01338-sp3 | 0.412768633 | 0.008375905 |
| gene-Led01343-sp3 | 0.968833069 | 1.91E-06 |
| gene-Led01347-sp3 | -1.158906975 | 0.000146736 |
| gene-Led01372-sp3 | 0.460310923 | 0.005335666 |
| gene-Led01435-sp3 | 0.940626476 | 0.000176622 |
| gene-Led01444-sp3 | 0.376449756 | 0.0066546 |
| gene-Led01468-sp3 | -0.752707886 | 0.005544141 |
| gene-Led01475-sp3 | -0.845748222 | 0.005585884 |
| gene-Led01527-sp3 | 0.593277961 | 0.001379366 |
| gene-Led01530-sp3 | 0.519794123 | 0.00131829 |
| gene-Led01536-sp3 | 1.568096041 | 3.84E-14 |
| gene-Led01538-sp3 | -1.409794555 | 2.78E-05 |
| gene-Led01543-sp3 | 0.532920756 | 0.001351585 |
| gene-Led01556-sp3 | -1.153278961 | 0.002644866 |
| gene-Led01568-sp3 | 0.731097593 | 0.001745897 |
| gene-Led01573-sp3 | 0.363461075 | 0.009138976 |
| gene-Led01609-sp3 | -1.474334129 | 1.03E-09 |
| gene-Led01612-sp3 | -3.36720449 | 2.26E-27 |
| gene-Led01618-sp3 | 0.542661562 | 0.000622 |
| gene-Led01619-sp3 | 0.651761068 | 0.000399337 |

| | | |
|---|---|---|
| gene-Led01636-sp3 | 0.624402142 | 0.002360445 |
| gene-Led01643-sp3 | 0.541079037 | 0.001974068 |
| gene-Led01665-sp3 | 0.663581678 | 1.01E-04 |
| gene-Led01667-sp3 | 0.595214525 | 0.00220211 |
| gene-Led01674-sp3 | 0.863763944 | 1.67E-06 |
| gene-Led01687-sp3 | -1.197971983 | 0.0001264 |
| gene-Led01698-sp3 | -1.296363652 | 3.37E-06 |
| gene-Led01699-sp3 | 0.385330283 | 0.00906599 |
| gene-Led01700-sp3 | 0.64980853 | 0.000139282 |
| gene-Led01707-sp3 | 0.807083149 | 8.68E-07 |
| gene-Led01719-sp3 | 1.341407114 | 0.002757787 |
| gene-Led01720-sp3 | 0.859461087 | 0.000146743 |
| gene-Led01721-sp3 | 0.414837473 | 0.004698725 |
| gene-Led01752-sp3 | 0.555916109 | 0.00210482 |
| gene-Led01757-sp3 | 0.594677289 | 0.002020359 |
| gene-Led01764-sp3 | -2.26707273 | 2.49E-11 |
| gene-Led01765-sp3 | -1.280841403 | 0.004763039 |
| gene-Led01821-sp3 | 1.263361825 | 0.000514036 |
| gene-Led01831-sp3 | -2.174222875 | 2.17E-13 |
| gene-Led01835-sp3 | -0.768440264 | 0.00614192 |
| gene-Led01861-sp3 | 0.511101536 | 0.0055287 |
| gene-Led01906-sp3 | 0.518074593 | 0.002061308 |
| gene-Led01938-sp3 | -1.712275961 | 9.56E-07 |
| gene-Led01951-sp3 | -1.982732825 | 0.0000556 |
| gene-Led01960-sp3 | 1.293693699 | 5.22E-09 |
| gene-Led01961-sp3 | -1.127110125 | 5.50E-06 |
| gene-Led01968-sp3 | -0.733066019 | 0.001496261 |
| gene-Led01978-sp3 | -1.567753224 | 6.03E-07 |
| gene-Led02006-sp3 | 0.575295116 | 0.000255131 |
| gene-Led02046-sp3 | 0.579417946 | 0.000199804 |
| gene-Led02047-sp3 | 0.70243331 | 2.22E-05 |
| gene-Led02053-sp3 | 0.543207722 | 0.003552919 |
| gene-Led02063-sp3 | 0.908967779 | 9.74E-05 |
| gene-Led02086-sp3 | 0.681958011 | 6.16E-05 |
| gene-Led02107-sp3 | 0.494058921 | 0.008539402 |
| gene-Led02123-sp3 | 0.485767989 | 0.00355998 |
| gene-Led02127-sp3 | 0.457795424 | 0.003521464 |
| gene-Led02188-sp3 | -1.744664915 | 0.000243043 |
| gene-Led02200-sp3 | -0.756715132 | 0.004562518 |
| gene-Led02203-sp3 | 0.545331674 | 0.000711931 |
| gene-Led02238-sp3 | 1.219892412 | 1.10E-08 |
| gene-Led02239-sp3 | 0.615896218 | 0.002068042 |
| gene-Led02347-sp3 | 1.211958552 | 0.0000907 |
| gene-Led02368-sp3 | -1.517089991 | 1.88E-09 |

| | | |
|---|---|---|
| gene-Led02374-sp3 | 0.668269905 | 0.000562922 |
| gene-Led02383-sp3 | 0.420208451 | 0.008225588 |
| gene-Led02387-sp3 | 0.47033205 | 0.009326515 |
| gene-Led02397-sp3 | -1.526527406 | 9.74E-07 |
| gene-Led02438-sp3 | -3.349641927 | 9.25E-16 |
| gene-Led02486-sp3 | 0.477393524 | 0.00658877 |
| gene-Led02526-sp3 | 0.596967916 | 0.001711357 |
| gene-Led02550-sp3 | -0.924320036 | 0.000874888 |
| gene-Led02625-sp3 | -2.845216356 | 2.51E-15 |
| gene-Led02639-sp3 | 0.710818403 | 2.62E-05 |
| gene-Led02640-sp3 | 1.366819707 | 0.004151835 |
| gene-Led02648-sp3 | 0.651766439 | 0.000460901 |
| gene-Led02690-sp3 | 2.156387369 | 1.11E-09 |
| gene-Led02705-sp3 | 0.50945261 | 0.001271566 |
| gene-Led02746-sp3 | -1.004523039 | 1.69E-06 |
| gene-Led02753-sp3 | 0.569436985 | 0.002203103 |
| gene-Led02755-sp3 | 0.688137767 | 0.00016451 |
| gene-Led02776-sp3 | 0.572515178 | 0.001902764 |
| gene-Led02777-sp3 | 0.545489516 | 0.000544514 |
| gene-Led02787-sp3 | 0.477353608 | 0.00304617 |
| gene-Led02838-sp3 | 0.595777766 | 0.000665401 |
| gene-Led02883-sp3 | -1.400712251 | 0.0000916 |
| gene-Led02903-sp3 | -0.490564537 | 0.007707069 |
| gene-Led02908-sp3 | 0.534814018 | 0.001151026 |
| gene-Led02922-sp3 | 0.591587722 | 0.009788794 |
| gene-Led02941-sp3 | 0.557803639 | 0.000750812 |
| gene-Led02958-sp3 | 0.58419066 | 0.000644468 |
| gene-Led02981-sp3 | 0.667114948 | 0.000147988 |
| gene-Led02988-sp3 | 0.399180446 | 0.008247062 |
| gene-Led02995-sp3 | 0.414013415 | 0.005863489 |
| gene-Led03000-sp3 | 3.27898275 | 0.000000469 |
| gene-Led03018-sp3 | -0.62339642 | 0.001471208 |
| gene-Led03030-sp3 | 0.935442366 | 0.000186217 |
| gene-Led03038-sp3 | 0.598022679 | 0.000336225 |
| gene-Led03044-sp3 | 0.469306005 | 0.004742735 |
| gene-Led03047-sp3 | 0.473982602 | 0.005532338 |
| gene-Led03048-sp3 | 0.406485221 | 0.007248918 |
| gene-Led03053-sp3 | 0.846170989 | 1.18E-05 |
| gene-Led03053-sp3 | 0.861325419 | 6.62E-05 |
| gene-Led03054-sp3 | 0.706251678 | 1.03E-05 |
| gene-Led03076-sp3 | 1.318526425 | 2.80E-08 |
| gene-Led03111-sp3 | 0.446312671 | 0.003951701 |
| gene-Led03207-sp3 | 0.490103737 | 0.004455711 |
| gene-Led03208-sp3 | 1.285163514 | 2.58E-09 |

| | | |
|---|---|---|
| gene-Led03227-sp3 | 0.568008426 | 0.004224649 |
| gene-Led03250-sp3 | -0.842926009 | 0.003252644 |
| gene-Led03256-sp3 | 0.399616729 | 0.00306749 |
| gene-Led03324-sp3 | 0.337602449 | 0.009160127 |
| gene-Led03328-sp3 | -2.187555986 | 1.34E-09 |
| gene-Led03375-sp3 | 0.412810175 | 0.009681925 |
| gene-Led03423-sp3 | 0.843093034 | 0.001188569 |
| gene-Led03434-sp3 | -1.717407578 | 2.20E-05 |
| gene-Led03440-sp3 | 0.74081686 | 0.00261754 |
| gene-Led03446-sp3 | 0.550041101 | 0.000384089 |
| gene-Led03464-sp3 | 0.859471334 | 0.0000999 |
| gene-Led03466-sp3 | -1.366829164 | 0.0000178 |
| gene-Led03481-sp3 | -0.90457335 | 0.001942902 |
| gene-Led03484-sp3 | -1.471479453 | 0.0000772 |
| gene-Led03566-sp3 | 0.47012557 | 0.005034201 |
| gene-Led03576-sp3 | -0.686283091 | 0.001229902 |
| gene-Led03580-sp3 | 0.77359489 | 5.28E-05 |
| gene-Led03596-sp3 | -0.676460453 | 0.001152268 |
| gene-Led03597-sp3 | 0.627398205 | 0.001124755 |
| gene-Led03620-sp3 | 0.74472172 | 1.01E-04 |
| gene-Led03682-sp3 | 0.539623396 | 0.006641355 |
| gene-Led03763-sp3 | 0.690976498 | 0.001372273 |
| gene-Led03765-sp3 | -0.964090169 | 3.74E-06 |
| gene-Led03786-sp3 | 0.918924444 | 0.003259573 |
| gene-Led03830-sp3 | 0.907649554 | 2.62E-06 |
| gene-Led03842-sp3 | -2.27609781 | 3.57E-10 |
| gene-Led03887-sp3 | -1.027656213 | 1.41E-06 |
| gene-Led03889-sp3 | 0.624267598 | 0.000339744 |
| gene-Led03890-sp3 | 0.568506026 | 0.000578855 |
| gene-Led03896-sp3 | -1.081206054 | 0.000470782 |
| gene-Led03897-sp3 | -1.831417068 | 6.09E-14 |
| gene-Led03898-sp3 | -1.549870075 | 1.23E-18 |
| gene-Led03900-sp3 | 0.46245791 | 0.00789425 |
| gene-Led03907-sp3 | -1.308403686 | 0.001451725 |
| gene-Led03916-sp3 | 1.74554897 | 0.000928318 |
| gene-Led03927-sp3 | -0.627262671 | 0.007075136 |
| gene-Led03966-sp3 | 0.392513527 | 0.00975904 |
| gene-Led03994-sp3 | 3.330407912 | 0.001260875 |
| gene-Led03997-sp3 | 0.637819087 | 0.000375196 |
| gene-Led04039-sp3 | 0.471647508 | 0.006165938 |
| gene-Led04040-sp3 | 0.99289736 | 1.69E-07 |
| gene-Led04060-sp3 | 0.468825059 | 0.003765726 |
| gene-Led04069-sp3 | 0.634617276 | 0.000723521 |
| gene-Led04098-sp3 | 0.549369842 | 0.002666435 |

| | | |
|---|---|---|
| gene-Led04102-sp3 | 1.954369487 | 0.00206219 |
| gene-Led04106-sp3 | 0.407276702 | 0.003502997 |
| gene-Led04107-sp3 | 0.542177756 | 0.000674746 |
| gene-Led04121-sp3 | -0.559496778 | 0.002727986 |
| gene-Led04132-sp3 | 0.44903468 | 0.002962063 |
| gene-Led04181-sp3 | -0.6672486 | 0.008368495 |
| gene-Led04243-sp3 | 0.676269651 | 0.000158754 |
| gene-Led04279-sp3 | -2.19442334 | 8.35E-19 |
| gene-Led04285-sp3 | -0.775245746 | 0.000192096 |
| gene-Led04286-sp3 | 0.526797139 | 0.001497648 |
| gene-Led04298-sp3 | 0.658524836 | 8.53E-05 |
| gene-Led04303-sp3 | 1.117273189 | 1.31E-07 |
| gene-Led04322-sp3 | 0.80006066 | 6.04E-06 |
| gene-Led04323-sp3 | 0.756251269 | 1.32E-06 |
| gene-Led04345-sp3 | -2.105219312 | 1.58E-09 |
| gene-Led04362-sp3 | 0.484198591 | 0.006766338 |
| gene-Led04364-sp3 | 0.442020518 | 0.003649561 |
| gene-Led04373-sp3 | -0.680087983 | 0.002003698 |
| gene-Led04399-sp3 | 0.725228741 | 0.003808327 |
| gene-Led04417-sp3 | -0.464904818 | 0.008517764 |
| gene-Led04429-sp3 | -0.623740588 | 0.000335308 |
| gene-Led04483-sp3 | -3.007247873 | 0.000000111 |
| gene-Led04484-sp3 | -1.674190174 | 0.001215107 |
| gene-Led04499-sp3 | -0.937482603 | 0.000811646 |
| gene-Led04523-sp3 | 0.482654662 | 0.002952892 |
| gene-Led04590-sp3 | -0.785562858 | 0.004115885 |
| gene-Led04617-sp3 | -2.067632404 | 5.05E-10 |
| gene-Led04637-sp3 | 0.508634421 | 0.0020094 |
| gene-Led04649-sp3 | -0.873953334 | 0.001907518 |
| gene-Led04656-sp3 | 0.570711656 | 0.001976843 |
| gene-Led04683-sp3 | -1.450363864 | 1.74E-08 |
| gene-Led04748-sp3 | -0.754671594 | 0.005301498 |
| gene-Led04755-sp3 | 0.443574767 | 0.009763024 |
| gene-Led04763-sp3 | -0.757712326 | 2.04E-05 |
| gene-Led04796-sp3 | -0.928486029 | 0.000885528 |
| gene-Led04806-sp3 | -0.833016563 | 0.001674533 |
| gene-Led04883-sp3 | -0.85069637 | 0.004125352 |
| gene-Led04936-sp3 | 0.557173513 | 0.001627985 |
| gene-Led04949-sp3 | -0.77348574 | 1.51E-05 |
| gene-Led04975-sp3 | -1.071750886 | 4.26E-06 |
| gene-Led04976-sp3 | -1.219412626 | 0.001658525 |
| gene-Led05017-sp3 | -0.767794697 | 0.008612855 |
| gene-Led05019-sp3 | 0.442905541 | 0.007285099 |
| gene-Led05022-sp3 | -1.303125923 | 5.27E-06 |

| | | |
|---|---|---|
| gene-Led05029-sp3 | 0.502179029 | 0.002348421 |
| gene-Led05031-sp3 | 1.478860387 | 4.68E-06 |
| gene-Led05034-sp3 | 1.022112166 | 0.000153941 |
| gene-Led05036-sp3 | -1.307535111 | 8.94E-05 |
| gene-Led05063-sp3 | 0.593889203 | 0.009796794 |
| gene-Led05068-sp3 | -2.641835988 | 6.38E-08 |
| gene-Led05069-sp3 | 0.466630457 | 0.006936527 |
| gene-Led05085-sp3 | 0.545733531 | 0.000475515 |
| gene-Led05089-sp3 | 0.410938354 | 0.00950963 |
| gene-Led05095-sp3 | -0.548479398 | 0.00305703 |
| gene-Led05130-sp3 | -0.693027392 | 0.000948236 |
| gene-Led05133-sp3 | 1.016820886 | 0.000110886 |
| gene-Led05164-sp3 | -0.71481558 | 0.009108252 |
| gene-Led05200-sp3 | 0.624156882 | 0.004252677 |
| gene-Led05219-sp3 | -1.231222709 | 3.84E-06 |
| gene-Led05248-sp3 | -1.182859602 | 1.49E-05 |
| gene-Led05248-sp3 | 0.722975582 | 0.00175655 |
| gene-Led05272-sp3 | 0.519821381 | 0.002173396 |
| gene-Led05273-sp3 | 1.311687737 | 3.08E-08 |
| gene-Led05300-sp3 | 1.80501089 | 7.99E-11 |
| gene-Led05321-sp3 | 0.560707427 | 0.000992326 |
| gene-Led05328-sp3 | -1.023263529 | 4.23E-05 |
| gene-Led05331-sp3 | 0.629539696 | 0.000550893 |
| gene-Led05332-sp3 | -0.894666639 | 0.000879153 |
| gene-Led05462-sp3 | -0.649649844 | 0.000122202 |
| gene-Led05501-sp3 | 0.502252592 | 0.002297045 |
| gene-Led05511-sp3 | 0.557482888 | 0.000849661 |
| gene-Led05539-sp3 | 0.675201996 | 0.009067559 |
| gene-Led05558-sp3 | -0.54160672 | 0.006520138 |
| gene-Led05573-sp3 | 0.573899459 | 0.000790667 |
| gene-Led05597-sp3 | 0.649054682 | 0.000326541 |
| gene-Led05629-sp3 | 0.435999718 | 0.004216441 |
| gene-Led05652-sp3 | 0.495026239 | 0.002429828 |
| gene-Led05686-sp3 | 0.411709737 | 0.007312493 |
| gene-Led05718-sp3 | -1.35960872 | 0.000294444 |
| gene-Led05768-sp3 | 0.508175657 | 0.00144806 |
| gene-Led05778-sp3 | 0.454214173 | 0.004078472 |
| gene-Led05789-sp3 | 0.861770874 | 1.14E-05 |
| gene-Led05795-sp3 | 2.237232525 | 0.00000947 |
| gene-Led05802-sp3 | 0.596880121 | 0.001562188 |
| gene-Led05804-sp3 | 0.618613751 | 0.000487095 |
| gene-Led05807-sp3 | 0.666153571 | 0.000565341 |
| gene-Led05808-sp3 | 1.288114747 | 9.70E-10 |
| gene-Led05940-sp3 | -0.570633452 | 0.003125862 |

| | | |
|---|---|---|
| gene-Led05961-sp3 | 2.470863441 | 0.001110602 |
| gene-Led06015-sp3 | 1.047035051 | 0.002226665 |
| gene-Led06029-sp3 | 1.261381248 | 1.02E-06 |
| gene-Led06034-sp3 | 0.913040084 | 0.0000992 |
| gene-Led06035-sp3 | 0.935077586 | 0.000325321 |
| gene-Led06059-sp3 | 1.774794063 | 8.65E-15 |
| gene-Led06060-sp3 | 0.821905881 | 3.24E-06 |
| gene-Led06083-sp3 | -0.598028706 | 0.004076242 |
| gene-Led06149-sp3 | -2.499983789 | 3.57E-25 |
| gene-Led06150-sp3 | -2.159316103 | 1.99E-18 |
| gene-Led06164-sp3 | 0.591542417 | 0.006062193 |
| gene-Led06253-sp3 | -1.025767576 | 3.80E-10 |
| gene-Led06260-sp3 | -0.686377824 | 0.005321286 |
| gene-Led06278-sp3 | 0.586206967 | 0.001768791 |
| gene-Led06279-sp3 | 0.570198374 | 0.00542917 |
| gene-Led06338-sp3 | 0.821878147 | 0.000506561 |
| gene-Led06351-sp3 | -0.846911264 | 0.002228649 |
| gene-Led06363-sp3 | 0.418075153 | 0.003703474 |
| gene-Led06365-sp3 | 0.717835505 | 0.000406405 |
| gene-Led06391-sp3 | 0.51141922 | 0.003213766 |
| gene-Led06448-sp3 | 0.473396045 | 0.009188109 |
| gene-Led06450-sp3 | 0.561724038 | 0.001381735 |
| gene-Led06456-sp3 | -0.900930713 | 0.000187913 |
| gene-Led06460-sp3 | 0.631311925 | 0.000348456 |
| gene-Led06477-sp3 | 1.002329448 | 0.002066935 |
| gene-Led06490-sp3 | 0.409219435 | 0.00589346 |
| gene-Led06497-sp3 | 0.632285386 | 2.36E-05 |
| gene-Led06499-sp3 | -1.303777371 | 0.004948911 |
| gene-Led06500-sp3 | 0.576096361 | 0.003835086 |
| gene-Led06516-sp3 | 0.716776551 | 0.000450631 |
| gene-Led06524-sp3 | -1.007977616 | 7.50E-06 |
| gene-Led06540-sp3 | 0.465176613 | 0.006656611 |
| gene-Led06546-sp3 | -1.187320366 | 0.002008306 |
| gene-Led06552-sp3 | 0.472869081 | 0.001874604 |
| gene-Led06565-sp3 | 0.785698014 | 0.000837605 |
| gene-Led06582-sp3 | -1.002410276 | 1.08E-05 |
| gene-Led06586-sp3 | 0.866540516 | 0.008609514 |
| gene-Led06587-sp3 | 0.42423792 | 0.004258065 |
| gene-Led06601-sp3 | 0.812203839 | 3.91E-06 |
| gene-Led06611-sp3 | 0.549554743 | 0.000682253 |
| gene-Led06632-sp3 | 0.63723538 | 0.000472363 |
| gene-Led06637-sp3 | 0.523098798 | 0.00179398 |
| gene-Led06646-sp3 | 0.767251826 | 3.20E-05 |
| gene-Led06661-sp3 | 0.563002909 | 0.00070752 |

| | | |
|---|---|---|
| gene-Led06662-sp3 | 0.59259674 | 0.000359521 |
| gene-Led06674-sp3 | 0.602895325 | 0.000448395 |
| gene-Led06679-sp3 | -0.619255698 | 0.00262941 |
| gene-Led06743-sp3 | 0.497582684 | 0.001875283 |
| gene-Led06804-sp3 | 0.43369247 | 0.00954411 |
| gene-Led06807-sp3 | -0.795192974 | 0.004984331 |
| gene-Led06873-sp3 | -0.831728821 | 1.27E-05 |
| gene-Led06888-sp3 | -0.2140026 | 0.004332404 |
| gene-Led06902-sp3 | 0.432931449 | 0.005306961 |
| gene-Led06911-sp3 | 1.354151029 | 0.000350314 |
| gene-Led06950-sp3 | 0.516139561 | 0.003655779 |
| gene-Led06957-sp3 | -0.748562913 | 0.001172595 |
| gene-Led07002-sp3 | 1.561971565 | 1.21E-08 |
| gene-Led07008-sp3 | -1.216344228 | 1.00E-05 |
| gene-Led07046-sp3 | 0.532685555 | 0.003129974 |
| gene-Led07100-sp3 | -1.612497134 | 1.57E-06 |
| gene-Led07102-sp3 | -2.057218154 | 4.47E-20 |
| gene-Led07143-sp3 | 0.754279265 | 0.000376701 |
| gene-Led07148-sp3 | 1.984772254 | 0.000143067 |
| gene-Led07155-sp3 | -0.546250717 | 0.007153299 |
| gene-Led07186-sp3 | 0.894015201 | 2.40E-05 |
| gene-Led07201-sp3 | -0.803866499 | 0.003072457 |
| gene-Led07210-sp3 | 0.585928902 | 0.003872744 |
| gene-Led07217-sp3 | 1.221670716 | 3.66E-06 |
| gene-Led07218-sp3 | 0.747270759 | 0.000946925 |
| gene-Led07222-sp3 | 0.774801295 | 0.000297252 |
| gene-Led07224-sp3 | 0.598475613 | 0.006725162 |
| gene-Led07227-sp3 | -0.691315154 | 0.001472049 |
| gene-Led07305-sp3 | 0.499598739 | 0.003399243 |
| gene-Led07321-sp3 | 0.562106341 | 0.000203065 |
| gene-Led07327-sp3 | -0.58289099 | 0.003817738 |
| gene-Led07328-sp3 | -0.58289099 | 0.003817738 |
| gene-Led07329-sp3 | -0.58289099 | 0.003817738 |
| gene-Led07332-sp3 | -0.58289099 | 0.003817738 |
| gene-Led07334-sp3 | 0.040894385 | 0.000427612 |
| gene-Led07335-sp3 | -0.58289099 | 0.003817738 |
| gene-Led07362-sp3 | 0.728603149 | 6.56E-06 |
| gene-Led07391-sp3 | -1.615738612 | 2.44E-07 |
| gene-Led07419-sp3 | 0.656923074 | 0.002902299 |
| gene-Led07489-sp3 | 0.578707778 | 0.00042075 |
| gene-Led07503-sp3 | 0.999997177 | 2.25E-06 |
| gene-Led07506-sp3 | -1.029178299 | 4.97E-06 |
| gene-Led07513-sp3 | 1.097292515 | 0.000687292 |
| gene-Led07517-sp3 | 0.623786977 | 0.000390743 |

| | | |
|---|---|---|
| gene-Led07518-sp3 | 0.46740763 | 0.0022144 |
| gene-Led07525-sp3 | 0.447920592 | 0.009062248 |
| gene-Led07526-sp3 | -0.95666465 | 0.004155938 |
| gene-Led07537-sp3 | 0.405880779 | 0.006348509 |
| gene-Led07552-sp3 | -0.848338308 | 0.001718968 |
| gene-Led07571-sp3 | 0.448942877 | 0.003521791 |
| gene-Led07581-sp3 | 0.630595146 | 1.92E-05 |
| gene-Led07604-sp3 | 0.862356381 | 1.61E-06 |
| gene-Led07626-sp3 | -0.67382655 | 0.001123639 |
| gene-Led07685-sp3 | 1.284576308 | 1.56E-06 |
| gene-Led07695-sp3 | 0.522826391 | 0.001316986 |
| gene-Led07705-sp3 | 0.418922064 | 0.004200326 |
| gene-Led07712-sp3 | 0.715116539 | 5.88E-05 |
| gene-Led07718-sp3 | 0.435056032 | 0.007747226 |
| gene-Led07720-sp3 | 0.436332811 | 0.003982004 |
| gene-Led07765-sp3 | 0.528168216 | 0.00312546 |
| gene-Led07782-sp3 | -1.037995078 | 0.000435593 |
| gene-Led07793-sp3 | 0.636162569 | 0.000775749 |
| gene-Led07803-sp3 | -0.834602375 | 0.003205262 |
| gene-Led07810-sp3 | 0.569671953 | 0.001493256 |
| gene-Led07827-sp3 | 0.557386732 | 0.00094675 |
| gene-Led07842-sp3 | 0.654001381 | 0.00105046 |
| gene-Led07853-sp3 | 0.577959253 | 0.002725344 |
| gene-Led07862-sp3 | 0.415382558 | 0.004728859 |
| gene-Led07891-sp3 | 0.463239161 | 0.004061885 |
| gene-Led07902-sp3 | 0.446921007 | 0.003679626 |
| gene-Led07918-sp3 | 0.850997055 | 9.55E-06 |
| gene-Led07925-sp3 | 0.55852032 | 0.000870817 |
| gene-Led07958-sp3 | -1.057279717 | 0.000308881 |
| gene-Led07963-sp3 | -0.825533926 | 1.08E-04 |
| gene-Led08017-sp3 | 0.601692055 | 0.00060157 |
| gene-Led08026-sp3 | 1.329828493 | 0.004373417 |
| gene-Led08036-sp3 | 0.76538722 | 0.00028604 |
| gene-Led08044-sp3 | 0.361124653 | 0.008075849 |
| gene-Led08091-sp3 | 0.686028212 | 0.000786386 |
| gene-Led08123-sp3 | 0.496478454 | 0.001175529 |
| gene-Led08127-sp3 | 0.539563395 | 0.000942051 |
| gene-Led08187-sp3 | -1.791238375 | 0.000148017 |
| gene-Led08204-sp3 | 0.574930725 | 0.000479205 |
| gene-Led08218-sp3 | -1.550954596 | 3.17E-08 |
| gene-Led08224-sp3 | 1.890577892 | 0.000186212 |
| gene-Led08257-sp3 | -1.859708878 | 0.00000392 |
| gene-Led08281-sp3 | 0.559618161 | 0.001264592 |
| gene-Led08308-sp3 | 0.417778701 | 0.005073747 |

| | | |
|---|---|---|
| gene-Led08389-sp3 | -0.563473285 | 0.003379629 |
| gene-Led08431-sp3 | 0.512448113 | 0.000565528 |
| gene-Led08460-sp3 | -1.621580645 | 1.69E-07 |
| gene-Led08519-sp3 | 0.471331724 | 0.002006338 |
| gene-Led08615-sp3 | 0.547528665 | 0.000481677 |
| gene-Led08636-sp3 | -0.787724802 | 0.000366889 |
| gene-Led08733-sp3 | -1.063604462 | 4.00E-05 |
| gene-Led08734-sp3 | -1.253986816 | 2.42E-05 |
| gene-Led08757-sp3 | -0.5421189 | 0.004455919 |
| gene-Led08786-sp3 | -0.723394649 | 0.001023856 |
| gene-Led08812-sp3 | 0.462252047 | 0.001506384 |
| gene-Led08827-sp3 | 0.926218577 | 1.77E-08 |
| gene-Led08829-sp3 | 0.917321334 | 1.68E-05 |
| gene-Led08838-sp3 | 1.081098759 | 7.86E-09 |
| gene-Led08840-sp3 | -1.089027748 | 0.003032985 |
| gene-Led08896-sp3 | -1.30180567 | 0.00037332 |
| gene-Led08908-sp3 | 1.103046558 | 1.98E-06 |
| gene-Led09023-sp3 | 1.56522619 | 5.18E-11 |
| gene-Led09024-sp3 | -0.430498988 | 0.008916975 |
| gene-Led09039-sp3 | 0.642059613 | 0.006164279 |
| gene-Led09044-sp3 | 0.597316303 | 0.001294014 |
| gene-Led09054-sp3 | -0.917424261 | 1.18E-06 |
| gene-Led09072-sp3 | 0.397117782 | 0.009220241 |
| gene-Led09096-sp3 | 0.664720116 | 0.000155778 |
| gene-Led09160-sp3 | -0.564299296 | 0.009602631 |
| gene-Led09161-sp3 | -1.466238435 | 0.00000717 |
| gene-Led09163-sp3 | 0.544120378 | 0.001362881 |
| gene-Led09169-sp3 | 0.381812825 | 0.00713185 |
| gene-Led09270-sp3 | -0.655348642 | 0.003592346 |
| gene-Led09272-sp3 | 0.444719073 | 0.005026708 |
| gene-Led09273-sp3 | 0.432258321 | 0.003977023 |
| gene-Led09277-sp3 | -0.754551443 | 0.005413194 |
| gene-Led09321-sp3 | -0.635537933 | 0.004774634 |
| gene-Led09355-sp3 | 0.434322657 | 0.007410461 |
| gene-Led09358-sp3 | -1.038712161 | 0.000201859 |
| gene-Led09364-sp3 | -1.25765717 | 0.000269817 |
| gene-Led09371-sp3 | 0.466011148 | 0.003342058 |
| gene-Led09418-sp3 | -0.802427098 | 0.001070931 |
| gene-Led09424-sp3 | -0.811925738 | 0.002419053 |
| gene-Led09430-sp3 | 0.838148758 | 1.50E-05 |
| gene-Led09431-sp3 | 0.927895732 | 8.72E-05 |
| gene-Led09437-sp3 | 0.841722095 | 5.68E-06 |
| gene-Led09438-sp3 | 0.978841923 | 6.18E-08 |
| gene-Led09450-sp3 | 0.79495005 | 4.06E-05 |

| | | |
|---|---|---|
| gene-Led09462-sp3 | -1.218546756 | 0.002946143 |
| gene-Led09501-sp3 | 0.409443374 | 0.004437762 |
| gene-Led09516-sp3 | 0.860879118 | 3.60E-05 |
| gene-Led09517-sp3 | 0.547016127 | 0.001509426 |
| gene-Led09577-sp3 | -0.733472244 | 0.005279 |
| gene-Led09604-sp3 | 0.498151485 | 0.004059572 |
| gene-Led09616-sp3 | -1.866673632 | 3.23E-08 |
| gene-Led09617-sp3 | -1.058063341 | 7.48E-06 |
| gene-Led09685-sp3 | 1.086157613 | 0.000429548 |
| gene-Led09702-sp3 | -0.844320491 | 0.000313765 |
| gene-Led09712-sp3 | -0.891537628 | 0.004583175 |
| gene-Led09731-sp3 | 0.395517657 | 0.009637382 |
| gene-Led09734-sp3 | -0.229155025 | 0.005969013 |
| gene-Led09825-sp3 | 0.518349448 | 0.002148045 |
| gene-Led09885-sp3 | -1.295937684 | 2.59E-07 |
| gene-Led09984-sp3 | 0.628127803 | 0.000574396 |
| gene-Led09997-sp3 | 1.311139965 | 0.006962941 |
| gene-Led10016-sp3 | 0.613715353 | 0.000277922 |
| gene-Led10040-sp3 | 0.439596104 | 0.003398933 |
| gene-Led10041-sp3 | 0.607786412 | 0.001240845 |
| gene-Led10046-sp3 | -1.029005032 | 1.76E-05 |
| gene-Led10083-sp3 | 0.872338414 | 0.000187026 |
| gene-Led10088-sp3 | 0.613252571 | 0.000496484 |
| gene-Led10102-sp3 | 0.673260651 | 0.000130544 |
| gene-Led10145-sp3 | -0.49598177 | 0.000983408 |
| gene-Led10228-sp3 | -0.938383023 | 0.000175938 |
| gene-Led10235-sp3 | -1.202352003 | 0.001378633 |
| gene-Led10238-sp3 | -1.431439897 | 0.000941878 |
| gene-Led10239-sp3 | 1.238727046 | 5.15E-09 |
| gene-Led10299-sp3 | -0.79549449 | 0.005362152 |
| gene-Led10334-sp3 | -0.803584532 | 0.004155225 |
| gene-Led10376-sp3 | -0.635975853 | 0.003728101 |
| gene-Led10456-sp3 | 0.405000406 | 0.009348758 |
| gene-Led10484-sp3 | -0.841389814 | 4.78E-05 |
| gene-Led10492-sp3 | -0.775576754 | 0.000179867 |
| gene-Led10531-sp3 | -1.924617049 | 1.44E-17 |
| gene-Led10555-sp3 | -1.402820337 | 6.55E-08 |
| gene-Led10562-sp3 | 0.577141125 | 0.000421401 |
| gene-Led10584-sp3 | 0.710455082 | 2.69E-05 |
| gene-Led10587-sp3 | 0.632948637 | 0.000156029 |
| gene-Led10643-sp3 | 0.584088689 | 0.001911626 |
| gene-Led10674-sp3 | 0.496779091 | 0.001181524 |
| gene-Led10686-sp3 | 0.511504433 | 0.003285955 |
| gene-Led10746-sp3 | 0.610597495 | 0.001675596 |

| | | |
|---|---|---|
| gene-Led10757-sp3 | 0.946972275 | 0.000724195 |
| gene-Led10763-sp3 | -0.539499205 | 0.004904371 |
| gene-Led10768-sp3 | -0.537462053 | 0.004285673 |
| gene-Led10813-sp3 | 0.858744023 | 8.29E-06 |
| gene-Led10877-sp3 | 0.454047064 | 0.009199623 |
| gene-Led10888-sp3 | 0.544457477 | 0.002323114 |
| gene-Led10891-sp3 | 0.624397589 | 0.000294024 |
| gene-Led10892-sp3 | 1.706180349 | 0.0000216 |
| gene-Led10927-sp3 | 0.923933228 | 0.0053261 |
| gene-Led10930-sp3 | 0.614388099 | 0.000247512 |
| gene-Led10991-sp3 | 1.708639016 | 9.03E-12 |
| gene-Led10993-sp3 | 0.840937052 | 0.000154492 |
| gene-Led10998-sp3 | 0.76672857 | 0.002100344 |
| gene-Led11008-sp3 | -0.58289099 | 0.003817738 |
| gene-Led11010-sp3 | -0.58289099 | 0.003817738 |
| gene-Led11012-sp3 | -0.58289099 | 0.003817738 |
| gene-Led11122-sp3 | -1.303125923 | 5.27E-06 |
| gene-Led11182-sp3 | -0.541740775 | 0.003264716 |
| gene-Led11264-sp3 | 0.87459717 | 0.00202385 |
| gene-Led11436-sp3 | 0.498151485 | 0.004059572 |

**Table S4 DEG lists of LeHH24h vs LeHH0h**

| gene | log2FoldChange | pvalue |
|---|---|---|
| gene-Led00002-sp3 | 0.884658861 | 0.003860364 |
| gene-Led00032-sp3 | -0.980425368 | 0.0086175 |
| gene-Led00038-sp3 | -1.51862801 | 0.001126656 |
| gene-Led00039-sp3 | -0.961523732 | 0.002084475 |
| gene-Led00043-sp3 | -0.81163756 | 0.008795189 |
| gene-Led00080-sp3 | -1.045766383 | 0.00000303 |
| gene-Led00095-sp3 | 1.430407342 | 0.0000724 |
| gene-Led00100-sp3 | 0.978827461 | 0.00000848 |
| gene-Led00101-sp3 | -0.946623752 | 0.003151243 |
| gene-Led00117-sp3 | 1.114179743 | 0.00000132 |
| gene-Led00122-sp3 | 1.107675847 | 0.000877962 |
| gene-Led00127-sp3 | -4.550205967 | 2.05E-47 |
| gene-Led00130-sp3 | -1.656766831 | 0.000284865 |
| gene-Led00171-sp3 | -0.907061119 | 0.000568868 |
| gene-Led00180-sp3 | -1.674884685 | 9.04E-11 |
| gene-Led00196-sp3 | -1.14558838 | 0.000174151 |
| gene-Led00247-sp3 | -0.4289909 | 0.006675272 |
| gene-Led00250-sp3 | 0.706386425 | 0.001187254 |
| gene-Led00256-sp3 | 1.139814126 | 0.00024372 |
| gene-Led00278-sp3 | -1.167201306 | 0.001318276 |
| gene-Led00280-sp3 | 0.552140811 | 0.000579441 |
| gene-Led00287-sp3 | -0.526203731 | 0.008276073 |
| gene-Led00318-sp3 | 0.774131792 | 0.00000421 |
| gene-Led00326-sp3 | -2.462030874 | 4.02E-12 |
| gene-Led00329-sp3 | -0.818051129 | 0.009360569 |
| gene-Led00336-sp3 | -1.326524658 | 0.000000169 |
| gene-Led00345-sp3 | 1.262559219 | 0.005572555 |
| gene-Led00346-sp3 | 0.573012433 | 0.000124254 |
| gene-Led00395-sp3 | -1.41096077 | 0.0000132 |
| gene-Led00411-sp3 | -0.717670478 | 0.008442208 |
| gene-Led00433-sp3 | -1.91726536 | 0.0000173 |
| gene-Led00433-sp3 | -1.361759388 | 0.002748618 |
| gene-Led00452-sp3 | -0.625716414 | 0.000991527 |
| gene-Led00457-sp3 | 0.660847197 | 0.006844967 |
| gene-Led00473-sp3 | 1.640120934 | 0.000122309 |
| gene-Led00498-sp3 | 0.845743997 | 0.00502626 |
| gene-Led00503-sp3 | -1.093249963 | 5.26E-10 |
| gene-Led00518-sp3 | 0.728901541 | 0.003074949 |
| gene-Led00522-sp3 | 0.540866442 | 0.005020539 |
| gene-Led00534-sp3 | -2.547521365 | 2.18E-14 |
| gene-Led00538-sp3 | -1.566347621 | 0.002815963 |
| gene-Led00547-sp3 | 0.767526728 | 0.001179862 |

| | | |
|---|---|---|
| gene-Led00552-sp3 | -1.738281337 | 1.05E-10 |
| gene-Led00553-sp3 | -2.980125573 | 8.08E-27 |
| gene-Led00559-sp3 | -0.997626772 | 0.000907685 |
| gene-Led00561-sp3 | -0.594831293 | 0.005997328 |
| gene-Led00566-sp3 | 0.747937368 | 0.001019526 |
| gene-Led00576-sp3 | 0.735059443 | 0.002361563 |
| gene-Led00581-sp3 | 0.475370215 | 0.001576342 |
| gene-Led00589-sp3 | 0.395909585 | 0.008611365 |
| gene-Led00590-sp3 | 0.88964205 | 0.002225685 |
| gene-Led00619-sp3 | 1.226999274 | 0.00000186 |
| gene-Led00655-sp3 | 1.144118952 | 0.000000465 |
| gene-Led00661-sp3 | 0.457705332 | 0.000912759 |
| gene-Led00680-sp3 | -0.485073025 | 0.003261329 |
| gene-Led00683-sp3 | -3.708411653 | 4.56E-14 |
| gene-Led00693-sp3 | 0.684180846 | 0.000851814 |
| gene-Led00702-sp3 | 0.525242274 | 0.008034139 |
| gene-Led00709-sp3 | 2.94670599 | 1.07E-08 |
| gene-Led00727-sp3 | 0.757459856 | 0.005222107 |
| gene-Led00734-sp3 | 2.81779726 | 0.0000703 |
| gene-Led00741-sp3 | 2.672526421 | 0.0000129 |
| gene-Led00742-sp3 | 0.985988849 | 0.0000195 |
| gene-Led00748-sp3 | 0.536392849 | 0.005222844 |
| gene-Led00751-sp3 | 0.560110978 | 0.002220912 |
| gene-Led00753-sp3 | 0.516684212 | 0.003239784 |
| gene-Led00754-sp3 | 1.056522578 | 0.00000024 |
| gene-Led00777-sp3 | 0.971843266 | 2.83E-08 |
| gene-Led00809-sp3 | 0.717416798 | 0.004596496 |
| gene-Led00823-sp3 | -4.434161235 | 0.000844891 |
| gene-Led00833-sp3 | 0.793752339 | 0.004256536 |
| gene-Led00864-sp3 | 0.505872752 | 0.007108619 |
| gene-Led00883-sp3 | 1.140859116 | 0.0000742 |
| gene-Led00902-sp3 | -2.603914101 | 4.41E-28 |
| gene-Led00903-sp3 | -1.846444719 | 4.44E-17 |
| gene-Led00904-sp3 | -2.428295293 | 0.00000198 |
| gene-Led00905-sp3 | -3.494505695 | 7.05E-53 |
| gene-Led00906-sp3 | -2.190565021 | 9.6E-25 |
| gene-Led00907-sp3 | -3.61440125 | 9.34E-23 |
| gene-Led00907-sp3 | -2.993189488 | 1.89E-18 |
| gene-Led00908-sp3 | -1.178261644 | 4.76E-08 |
| gene-Led00909-sp3 | -1.859266113 | 9.39E-12 |
| gene-Led00910-sp3 | -1.701601388 | 1.02E-22 |
| gene-Led00911-sp3 | -2.756963103 | 0.000115025 |
| gene-Led00911-sp3 | -1.662322227 | 0.000372909 |
| gene-Led00912-sp3 | -3.289269524 | 0.000000479 |

| | | |
|---|---|---|
| gene-Led00918-sp3 | 1.592371982 | 0.000037 |
| gene-Led00921-sp3 | 0.511535916 | 0.001391457 |
| gene-Led00925-sp3 | -0.629519178 | 0.006207571 |
| gene-Led00926-sp3 | 1.909385877 | 0.00000067 |
| gene-Led00929-sp3 | -3.235093277 | 9.56E-21 |
| gene-Led00933-sp3 | -0.637448272 | 0.002240132 |
| gene-Led00934-sp3 | 0.696668766 | 0.003162645 |
| gene-Led00940-sp3 | 0.606536495 | 0.001172648 |
| gene-Led00943-sp3 | -1.342577782 | 1.34E-10 |
| gene-Led00975-sp3 | -1.394433889 | 0.0000726 |
| gene-Led00983-sp3 | 2.466245183 | 0.0000029 |
| gene-Led01009-sp3 | 1.015954252 | 0.000120327 |
| gene-Led01017-sp3 | 0.564324574 | 0.001972261 |
| gene-Led01028-sp3 | -0.916769985 | 0.0000227 |
| gene-Led01058-sp3 | 1.783605016 | 0.000901574 |
| gene-Led01075-sp3 | 0.779410962 | 0.0000519 |
| gene-Led01085-sp3 | 0.477707295 | 0.006901348 |
| gene-Led01098-sp3 | -1.77718044 | 1.81E-12 |
| gene-Led01116-sp3 | 1.071641069 | 0.00000102 |
| gene-Led01121-sp3 | -0.689899901 | 0.006852849 |
| gene-Led01142-sp3 | 0.817382011 | 0.000109768 |
| gene-Led01148-sp3 | -1.592182711 | 0.000237628 |
| gene-Led01166-sp3 | 2.654258792 | 1.45E-13 |
| gene-Led01180-sp3 | -2.714251574 | 9.24E-13 |
| gene-Led01196-sp3 | 0.948607299 | 0.004967029 |
| gene-Led01198-sp3 | -1.251840961 | 0.000439134 |
| gene-Led01201-sp3 | -2.973942251 | 8.32E-16 |
| gene-Led01215-sp3 | 0.471232954 | 0.008328937 |
| gene-Led01221-sp3 | -0.465189226 | 0.003983595 |
| gene-Led01231-sp3 | -0.888477793 | 0.0000765 |
| gene-Led01245-sp3 | -1.305177269 | 5.29E-10 |
| gene-Led01246-sp3 | 0.694993279 | 0.002624244 |
| gene-Led01247-sp3 | 0.704540514 | 0.001336773 |
| gene-Led01249-sp3 | -0.6704988 | 0.000442592 |
| gene-Led01254-sp3 | 0.590222151 | 0.001399318 |
| gene-Led01265-sp3 | 2.398446095 | 0.000857532 |
| gene-Led01276-sp3 | 1.898420098 | 2.28E-09 |
| gene-Led01279-sp3 | 0.567262241 | 0.005653005 |
| gene-Led01295-sp3 | 0.592385324 | 0.007960964 |
| gene-Led01300-sp3 | -1.625707613 | 0.001850407 |
| gene-Led01302-sp3 | 2.629550872 | 0.000000442 |
| gene-Led01305-sp3 | 0.82598816 | 0.0000847 |
| gene-Led01312-sp3 | 2.067826198 | 2.84E-10 |
| gene-Led01325-sp3 | -5.157532903 | 2.81E-56 |

| | | |
|---|---|---|
| gene-Led01338-sp3 | -0.545481648 | 0.003348085 |
| gene-Led01343-sp3 | 0.653873896 | 0.002474115 |
| gene-Led01345-sp3 | 1.243216171 | 0.000563745 |
| gene-Led01346-sp3 | -0.804394331 | 0.00000494 |
| gene-Led01347-sp3 | 1.067990052 | 0.0000464 |
| gene-Led01348-sp3 | -2.082411901 | 0.00036553 |
| gene-Led01359-sp3 | 1.53575967 | 0.000000642 |
| gene-Led01372-sp3 | 0.515043451 | 0.005478752 |
| gene-Led01379-sp3 | 0.725173553 | 0.005240704 |
| gene-Led01400-sp3 | 0.696543535 | 0.000153616 |
| gene-Led01407-sp3 | -1.874939818 | 0.000000147 |
| gene-Led01431-sp3 | 0.737166723 | 0.002826398 |
| gene-Led01443-sp3 | -1.360022995 | 0.000101215 |
| gene-Led01458-sp3 | 2.081239168 | 5.91E-13 |
| gene-Led01466-sp3 | -0.642670606 | 0.006192378 |
| gene-Led01472-sp3 | 1.659325713 | 2.47E-11 |
| gene-Led01475-sp3 | -1.502829529 | 0.000291067 |
| gene-Led01483-sp3 | -1.990325558 | 0.000522009 |
| gene-Led01492-sp3 | -1.065086174 | 0.001077992 |
| gene-Led01498-sp3 | 1.237582139 | 0.000000507 |
| gene-Led01530-sp3 | 0.462595721 | 0.004277678 |
| gene-Led01536-sp3 | -0.628324626 | 0.000201507 |
| gene-Led01537-sp3 | 2.201348565 | 0.00000914 |
| gene-Led01538-sp3 | 3.284571587 | 3.34E-33 |
| gene-Led01539-sp3 | 1.924743146 | 1.03E-11 |
| gene-Led01548-sp3 | 2.131050194 | 0.000982984 |
| gene-Led01550-sp3 | 1.597688791 | 0.00000054 |
| gene-Led01556-sp3 | -1.55758671 | 0.000585008 |
| gene-Led01557-sp3 | 2.438666048 | 0.0000259 |
| gene-Led01557-sp3 | 4.129170864 | 0.0000703 |
| gene-Led01567-sp3 | 3.049616694 | 7.47E-23 |
| gene-Led01568-sp3 | 1.68048791 | 5.32E-10 |
| gene-Led01578-sp3 | -1.644824183 | 0.00000077 |
| gene-Led01603-sp3 | -0.763307334 | 0.005166362 |
| gene-Led01608-sp3 | -0.45326999 | 0.004361348 |
| gene-Led01609-sp3 | -1.759783534 | 5.85E-13 |
| gene-Led01612-sp3 | -3.725642977 | 2.04E-30 |
| gene-Led01618-sp3 | 0.649969241 | 0.000137153 |
| gene-Led01630-sp3 | 1.602550971 | 0.001719029 |
| gene-Led01632-sp3 | 0.604446108 | 0.001878223 |
| gene-Led01634-sp3 | -0.991925435 | 0.0000688 |
| gene-Led01670-sp3 | 0.732407196 | 0.0000918 |
| gene-Led01673-sp3 | -1.521867648 | 4.12E-10 |
| gene-Led01674-sp3 | 0.518059721 | 0.004872619 |

| | | |
|---|---|---|
| gene-Led01680-sp3 | -1.056379126 | 0.001354748 |
| gene-Led01684-sp3 | -0.985088328 | 0.003729355 |
| gene-Led01687-sp3 | -2.388186548 | 5.08E-09 |
| gene-Led01693-sp3 | 1.113020952 | 0.00657145 |
| gene-Led01698-sp3 | -2.088491516 | 7.98E-11 |
| gene-Led01699-sp3 | 0.380604564 | 0.007700354 |
| gene-Led01700-sp3 | 0.585038923 | 0.000358106 |
| gene-Led01706-sp3 | 0.849580383 | 0.00000822 |
| gene-Led01707-sp3 | 0.847985854 | 0.000000412 |
| gene-Led01718-sp3 | -2.492676969 | 5.57E-16 |
| gene-Led01727-sp3 | 0.820058247 | 0.003727486 |
| gene-Led01736-sp3 | 0.483520393 | 0.002974127 |
| gene-Led01763-sp3 | 1.418210154 | 0.000037 |
| gene-Led01764-sp3 | 2.34563601 | 1.15E-12 |
| gene-Led01765-sp3 | 4.392257766 | 4.38E-38 |
| gene-Led01767-sp3 | 0.683804509 | 0.000732998 |
| gene-Led01769-sp3 | -0.56802338 | 0.001250905 |
| gene-Led01797-sp3 | 0.637693274 | 0.001826363 |
| gene-Led01806-sp3 | 0.964590734 | 0.0000682 |
| gene-Led01821-sp3 | 1.160187313 | 0.000762347 |
| gene-Led01821-sp3 | 1.429486584 | 0.001677067 |
| gene-Led01831-sp3 | -2.70277376 | 7.41E-15 |
| gene-Led01832-sp3 | 0.89670551 | 0.001360998 |
| gene-Led01835-sp3 | -1.251624739 | 0.000600308 |
| gene-Led01837-sp3 | 1.458909254 | 0.006724166 |
| gene-Led01838-sp3 | 1.866858594 | 0.000285999 |
| gene-Led01843-sp3 | 1.104837177 | 0.002566197 |
| gene-Led01859-sp3 | 2.526593313 | 1.25E-10 |
| gene-Led01902-sp3 | -0.496821214 | 0.006735554 |
| gene-Led01916-sp3 | -0.625912232 | 0.000216302 |
| gene-Led01936-sp3 | -1.130065282 | 0.007675403 |
| gene-Led01938-sp3 | -1.888768928 | 9.55E-08 |
| gene-Led01939-sp3 | -1.087329729 | 0.000685976 |
| gene-Led01940-sp3 | 0.598337594 | 0.003732111 |
| gene-Led01946-sp3 | -0.664366075 | 0.009701425 |
| gene-Led01950-sp3 | 1.117086009 | 0.001208523 |
| gene-Led01951-sp3 | -4.374233239 | 0.000000795 |
| gene-Led01961-sp3 | -1.064591632 | 0.0000242 |
| gene-Led01968-sp3 | -1.313806457 | 0.000000417 |
| gene-Led01973-sp3 | 0.909048425 | 0.002290296 |
| gene-Led01978-sp3 | -5.438044822 | 5.37E-15 |
| gene-Led02016-sp3 | 2.122771706 | 2.9E-14 |
| gene-Led02021-sp3 | 1.139496316 | 0.001103004 |
| gene-Led02054-sp3 | 0.864389311 | 0.001815486 |

| | | |
|---|---|---|
| gene-Led02063-sp3 | 1.682324464 | 1.12E-12 |
| gene-Led02064-sp3 | 0.796665549 | 0.00021758 |
| gene-Led02111-sp3 | 1.209886862 | 0.00000448 |
| gene-Led02154-sp3 | -2.081752985 | 0.0000944 |
| gene-Led02157-sp3 | 1.206679325 | 4.56E-09 |
| gene-Led02168-sp3 | 0.478671908 | 0.007578517 |
| gene-Led02181-sp3 | 0.653143629 | 0.006662686 |
| gene-Led02188-sp3 | -2.335605068 | 0.0000233 |
| gene-Led02212-sp3 | -0.878678013 | 0.001339995 |
| gene-Led02241-sp3 | 1.313383764 | 0.00031884 |
| gene-Led02251-sp3 | 1.462700913 | 0.000162679 |
| gene-Led02252-sp3 | 1.034770578 | 0.003371084 |
| gene-Led02273-sp3 | 1.883005411 | 1.1E-19 |
| gene-Led02291-sp3 | 1.57319343 | 0.000485072 |
| gene-Led02293-sp3 | 0.58275462 | 0.001738499 |
| gene-Led02300-sp3 | 0.809688481 | 0.000850924 |
| gene-Led02325-sp3 | 0.79896677 | 0.0000918 |
| gene-Led02347-sp3 | 1.048311516 | 0.000644341 |
| gene-Led02351-sp3 | 0.618868168 | 0.000525902 |
| gene-Led02374-sp3 | 0.73322228 | 0.000372049 |
| gene-Led02376-sp3 | -1.295069292 | 0.000102514 |
| gene-Led02377-sp3 | -1.662820622 | 0.00000214 |
| gene-Led02381-sp3 | 0.782213401 | 0.002301298 |
| gene-Led02383-sp3 | 0.496633828 | 0.000499922 |
| gene-Led02384-sp3 | 3.061432775 | 0.000519205 |
| gene-Led02397-sp3 | -0.885080883 | 0.002054972 |
| gene-Led02397-sp3 | 1.02076567 | 0.006156477 |
| gene-Led02417-sp3 | 0.749327316 | 0.0000137 |
| gene-Led02419-sp3 | 0.501744701 | 0.006804232 |
| gene-Led02438-sp3 | -5.136953843 | 1.29E-16 |
| gene-Led02440-sp3 | -2.127708772 | 7.43E-08 |
| gene-Led02452-sp3 | 0.558447919 | 0.001609939 |
| gene-Led02458-sp3 | -1.02392194 | 0.001032634 |
| gene-Led02478-sp3 | 0.857145176 | 0.001299274 |
| gene-Led02486-sp3 | 0.544929379 | 0.002328009 |
| gene-Led02494-sp3 | 3.342413469 | 1.28E-08 |
| gene-Led02500-sp3 | 3.087038209 | 0.0000573 |
| gene-Led02501-sp3 | 0.835507573 | 0.00000575 |
| gene-Led02516-sp3 | -1.311879384 | 0.000792413 |
| gene-Led02521-sp3 | 1.131696274 | 0.000000699 |
| gene-Led02526-sp3 | 0.6435428 | 0.003955121 |
| gene-Led02550-sp3 | -1.460883366 | 0.00000982 |
| gene-Led02558-sp3 | -1.104646042 | 0.000315533 |
| gene-Led02605-sp3 | 0.598158043 | 0.003131298 |

| | | |
|---|---|---|
| gene-Led02611-sp3 | 0.564362698 | 0.004300748 |
| gene-Led02625-sp3 | -2.512737119 | 2.29E-13 |
| gene-Led02640-sp3 | 2.250277666 | 0.0000893 |
| gene-Led02640-sp3 | -0.857194879 | 0.008550893 |
| gene-Led02666-sp3 | 0.40646783 | 0.008672399 |
| gene-Led02673-sp3 | 0.500938771 | 0.002096267 |
| gene-Led02690-sp3 | 1.095978129 | 0.001445712 |
| gene-Led02699-sp3 | 4.018346578 | 0.000014 |
| gene-Led02707-sp3 | 0.612264955 | 0.000461997 |
| gene-Led02726-sp3 | 0.674598022 | 0.003874191 |
| gene-Led02736-sp3 | 2.439960696 | 3.41E-20 |
| gene-Led02745-sp3 | 0.89475851 | 0.003914312 |
| gene-Led02747-sp3 | 1.050046588 | 0.000529099 |
| gene-Led02755-sp3 | -0.507143499 | 0.007155825 |
| gene-Led02757-sp3 | 1.646560122 | 1.26E-11 |
| gene-Led02766-sp3 | -0.911384352 | 0.006507655 |
| gene-Led02769-sp3 | 0.52245835 | 0.00402662 |
| gene-Led02776-sp3 | 1.617424105 | 3.14E-14 |
| gene-Led02804-sp3 | -0.407694068 | 0.002831496 |
| gene-Led02823-sp3 | 0.474832598 | 0.006483259 |
| gene-Led02850-sp3 | -1.058621342 | 0.000407626 |
| gene-Led02851-sp3 | 0.835153729 | 0.0000128 |
| gene-Led02859-sp3 | -1.028487782 | 1.47E-08 |
| gene-Led02883-sp3 | -2.575234165 | 0.000000025 |
| gene-Led02884-sp3 | 0.43037552 | 0.005372341 |
| gene-Led02893-sp3 | 0.77032127 | 0.00504444 |
| gene-Led02898-sp3 | -1.17653032 | 0.004310522 |
| gene-Led02900-sp3 | 0.945133516 | 0.00000135 |
| gene-Led02910-sp3 | -2.336809405 | 1.69E-17 |
| gene-Led02922-sp3 | 0.930858184 | 0.000620874 |
| gene-Led02941-sp3 | 1.067682263 | 0.000000022 |
| gene-Led02958-sp3 | 1.061393039 | 8.15E-10 |
| gene-Led02986-sp3 | -0.961555998 | 0.004180527 |
| gene-Led02992-sp3 | 0.95491769 | 0.006492992 |
| gene-Led03000-sp3 | 2.63547854 | 0.0000614 |
| gene-Led03028-sp3 | 0.53969679 | 0.003651501 |
| gene-Led03032-sp3 | 0.647022435 | 0.007697726 |
| gene-Led03038-sp3 | 0.699873051 | 0.000147451 |
| gene-Led03045-sp3 | 2.23430974 | 0.0000152 |
| gene-Led03047-sp3 | 0.461369525 | 0.003874711 |
| gene-Led03053-sp3 | 0.658442091 | 0.000612767 |
| gene-Led03055-sp3 | 0.893403352 | 0.00237094 |
| gene-Led03066-sp3 | 0.632403255 | 0.000132356 |
| gene-Led03076-sp3 | 0.769116584 | 0.000999384 |

| | | |
|---|---|---|
| gene-Led03084-sp3 | -0.855322839 | 0.000539926 |
| gene-Led03097-sp3 | 0.923217863 | 0.000000699 |
| gene-Led03128-sp3 | 0.548189356 | 0.002817855 |
| gene-Led03152-sp3 | 0.453925271 | 0.001928724 |
| gene-Led03154-sp3 | -0.992943115 | 0.0000253 |
| gene-Led03155-sp3 | -1.356380414 | 0.00000344 |
| gene-Led03165-sp3 | 0.742337687 | 0.002099215 |
| gene-Led03168-sp3 | 0.993697003 | 0.00000411 |
| gene-Led03172-sp3 | -2.734467842 | 0.00000124 |
| gene-Led03200-sp3 | 1.31054333 | 0.0000256 |
| gene-Led03201-sp3 | 2.329520425 | 2.52E-08 |
| gene-Led03203-sp3 | -1.470981107 | 1.32E-09 |
| gene-Led03208-sp3 | 0.87777888 | 0.00000578 |
| gene-Led03210-sp3 | 0.830441062 | 0.009592624 |
| gene-Led03217-sp3 | -1.545046613 | 0.003379033 |
| gene-Led03224-sp3 | 0.824279978 | 0.001714848 |
| gene-Led03227-sp3 | 0.580240478 | 0.005839117 |
| gene-Led03229-sp3 | 1.423099866 | 0.000323313 |
| gene-Led03234-sp3 | 0.541307712 | 0.004288991 |
| gene-Led03248-sp3 | -1.463701504 | 0.0000999 |
| gene-Led03250-sp3 | 0.939648652 | 0.00023312 |
| gene-Led03258-sp3 | 0.797787739 | 0.006289596 |
| gene-Led03270-sp3 | 1.438548605 | 0.000126523 |
| gene-Led03287-sp3 | 2.805171565 | 6.18E-20 |
| gene-Led03303-sp3 | 0.858844357 | 0.001695982 |
| gene-Led03323-sp3 | 0.907503935 | 0.00000546 |
| gene-Led03328-sp3 | -2.126005296 | 2.8E-09 |
| gene-Led03332-sp3 | -0.752801929 | 0.003347376 |
| gene-Led03343-sp3 | -0.926595747 | 0.006994418 |
| gene-Led03375-sp3 | 0.701125642 | 0.000116897 |
| gene-Led03404-sp3 | -1.016939675 | 0.009356242 |
| gene-Led03418-sp3 | -0.946825036 | 0.000428379 |
| gene-Led03423-sp3 | 0.86999493 | 0.001811935 |
| gene-Led03426-sp3 | 1.293377324 | 6.98E-09 |
| gene-Led03433-sp3 | 0.507390853 | 0.004419293 |
| gene-Led03434-sp3 | 1.818031736 | 0.000000157 |
| gene-Led03440-sp3 | 1.199483491 | 0.0000192 |
| gene-Led03441-sp3 | 1.227497206 | 0.0000121 |
| gene-Led03464-sp3 | 0.954200363 | 0.0000925 |
| gene-Led03466-sp3 | -1.32975829 | 0.0000223 |
| gene-Led03474-sp3 | 0.685359113 | 0.002954705 |
| gene-Led03486-sp3 | 0.742497073 | 0.001911697 |
| gene-Led03487-sp3 | 1.826429963 | 0.000128994 |
| gene-Led03490-sp3 | -0.59134758 | 0.005626059 |

| | | |
|---|---|---|
| gene-Led03515-sp3 | 0.533665053 | 0.001289918 |
| gene-Led03534-sp3 | -1.012955855 | 0.005443472 |
| gene-Led03538-sp3 | -1.043009035 | 0.000120072 |
| gene-Led03547-sp3 | 1.20202213 | 0.002408302 |
| gene-Led03557-sp3 | -2.079595362 | 2.39E-11 |
| gene-Led03570-sp3 | -1.799976378 | 0.000000266 |
| gene-Led03576-sp3 | 1.59900016 | 4.45E-11 |
| gene-Led03596-sp3 | -0.639540057 | 0.002008722 |
| gene-Led03597-sp3 | 0.849758061 | 0.000194297 |
| gene-Led03599-sp3 | 0.654572844 | 0.005972806 |
| gene-Led03620-sp3 | 0.585558498 | 0.001717144 |
| gene-Led03623-sp3 | 1.127562511 | 0.000119382 |
| gene-Led03628-sp3 | 1.266164414 | 0.00000211 |
| gene-Led03656-sp3 | 0.671022652 | 0.006557028 |
| gene-Led03670-sp3 | -1.016678288 | 0.00613838 |
| gene-Led03682-sp3 | -0.684192772 | 0.007336368 |
| gene-Led03684-sp3 | 0.892479337 | 0.0080061 |
| gene-Led03684-sp3 | 2.239919485 | 0.007762783 |
| gene-Led03699-sp3 | -1.899123125 | 0.001362675 |
| gene-Led03702-sp3 | 1.291913893 | 0.000000298 |
| gene-Led03708-sp3 | 1.02645596 | 0.007284315 |
| gene-Led03712-sp3 | 0.952675954 | 0.00000606 |
| gene-Led03736-sp3 | 1.680005705 | 0.000000124 |
| gene-Led03745-sp3 | -0.965465925 | 0.000100805 |
| gene-Led03748-sp3 | 1.734551018 | 0.000271422 |
| gene-Led03760-sp3 | -2.193064264 | 0.000372525 |
| gene-Led03765-sp3 | -0.8598829 | 0.00000788 |
| gene-Led03789-sp3 | -2.451654798 | 0.000000297 |
| gene-Led03811-sp3 | 0.695285277 | 0.000267845 |
| gene-Led03820-sp3 | 1.129019601 | 0.0000435 |
| gene-Led03839-sp3 | 1.458088343 | 0.0000928 |
| gene-Led03840-sp3 | 0.881236676 | 0.00476174 |
| gene-Led03841-sp3 | -0.984918819 | 0.0000275 |
| gene-Led03842-sp3 | -2.972462341 | 4.3E-13 |
| gene-Led03850-sp3 | -0.707584617 | 0.000228298 |
| gene-Led03859-sp3 | 0.70340981 | 0.000787307 |
| gene-Led03871-sp3 | 0.673491197 | 0.000202368 |
| gene-Led03878-sp3 | 0.427232563 | 0.006971124 |
| gene-Led03886-sp3 | 1.461102463 | 1.69E-12 |
| gene-Led03887-sp3 | 1.118354464 | 1.88E-08 |
| gene-Led03888-sp3 | 1.452898227 | 7.13E-14 |
| gene-Led03889-sp3 | 0.478960119 | 0.00181928 |
| gene-Led03890-sp3 | 0.468245993 | 0.001255794 |
| gene-Led03896-sp3 | 1.722691664 | 7.31E-12 |

| | | |
|---|---|---|
| gene-Led03897-sp3 | -1.58376794 | 7.11E-11 |
| gene-Led03898-sp3 | -1.00077261 | 3.39E-10 |
| gene-Led03899-sp3 | 0.655747252 | 0.004833205 |
| gene-Led03907-sp3 | 1.289909543 | 0.000151112 |
| gene-Led03908-sp3 | 2.215181614 | 0.002062558 |
| gene-Led03916-sp3 | 1.186762684 | 0.009093284 |
| gene-Led03922-sp3 | 1.229527696 | 0.000492429 |
| gene-Led03929-sp3 | -1.512779978 | 0.005331892 |
| gene-Led03932-sp3 | -2.566750245 | 3.9E-13 |
| gene-Led03934-sp3 | 0.490256805 | 0.005926559 |
| gene-Led03965-sp3 | 2.357949588 | 0.000280775 |
| gene-Led03968-sp3 | -0.927469045 | 0.004358891 |
| gene-Led03985-sp3 | -0.520200512 | 0.001474266 |
| gene-Led03989-sp3 | 0.975511981 | 0.000449382 |
| gene-Led04039-sp3 | 0.587855139 | 0.000207048 |
| gene-Led04040-sp3 | 0.642815608 | 0.000410883 |
| gene-Led04046-sp3 | -0.600040438 | 0.005183418 |
| gene-Led04057-sp3 | 0.608382296 | 0.004871305 |
| gene-Led04058-sp3 | 0.824922822 | 0.000871113 |
| gene-Led04062-sp3 | -0.830962538 | 0.000874807 |
| gene-Led04117-sp3 | 1.358158209 | 0.002293969 |
| gene-Led04121-sp3 | -1.890743279 | 4.99E-14 |
| gene-Led04123-sp3 | -1.113752272 | 0.000234982 |
| gene-Led04128-sp3 | 1.172076668 | 0.00000133 |
| gene-Led04131-sp3 | -2.369017177 | 3.39E-08 |
| gene-Led04139-sp3 | -0.770466962 | 0.003214681 |
| gene-Led04147-sp3 | 0.759131238 | 0.005647993 |
| gene-Led04157-sp3 | 1.287226321 | 0.0000673 |
| gene-Led04164-sp3 | 0.827354096 | 0.002679177 |
| gene-Led04170-sp3 | -1.084463284 | 0.004890638 |
| gene-Led04171-sp3 | -0.839539289 | 0.001827921 |
| gene-Led04173-sp3 | 0.673486955 | 0.003412538 |
| gene-Led04193-sp3 | 0.758002315 | 0.001879363 |
| gene-Led04203-sp3 | 1.442121234 | 2.29E-10 |
| gene-Led04215-sp3 | -0.757112735 | 0.006702296 |
| gene-Led04227-sp3 | 0.892867229 | 0.000165811 |
| gene-Led04228-sp3 | 0.929173627 | 0.00233066 |
| gene-Led04260-sp3 | -0.996040834 | 0.00257951 |
| gene-Led04261-sp3 | -1.394582037 | 0.00000687 |
| gene-Led04270-sp3 | 1.976556609 | 0.000402325 |
| gene-Led04270-sp3 | 1.077564726 | 0.003819793 |
| gene-Led04279-sp3 | -3.99216576 | 1.34E-39 |
| gene-Led04282-sp3 | 2.144270547 | 1.32E-09 |
| gene-Led04285-sp3 | -0.674803413 | 0.001551767 |

| | | |
|---|---|---|
| gene-Led04286-sp3 | 0.523240824 | 0.002180186 |
| gene-Led04295-sp3 | 0.601869484 | 0.006758934 |
| gene-Led04310-sp3 | -2.393788595 | 0.000041 |
| gene-Led04322-sp3 | 0.744387527 | 0.000000206 |
| gene-Led04323-sp3 | 0.614240723 | 0.00000715 |
| gene-Led04329-sp3 | 0.869064998 | 0.006630708 |
| gene-Led04336-sp3 | -3.195800667 | 5.23E-10 |
| gene-Led04345-sp3 | -4.596093261 | 2.78E-15 |
| gene-Led04348-sp3 | 1.441594657 | 0.000338317 |
| gene-Led04351-sp3 | -1.483706724 | 0.000535753 |
| gene-Led04358-sp3 | -0.777115125 | 0.00617359 |
| gene-Led04362-sp3 | 0.724386811 | 0.000139706 |
| gene-Led04363-sp3 | 0.767452468 | 0.000576638 |
| gene-Led04364-sp3 | 0.529495787 | 0.001895405 |
| gene-Led04370-sp3 | -3.161569103 | 0.000216845 |
| gene-Led04391-sp3 | 1.955009104 | 3.02E-13 |
| gene-Led04402-sp3 | 0.676448298 | 0.0000777 |
| gene-Led04429-sp3 | -1.516790926 | 2.77E-13 |
| gene-Led04444-sp3 | -0.865086369 | 0.00056083 |
| gene-Led04450-sp3 | -0.976879186 | 0.009186028 |
| gene-Led04463-sp3 | 0.913831891 | 0.000120562 |
| gene-Led04471-sp3 | -0.456849959 | 0.004426228 |
| gene-Led04483-sp3 | -3.200781462 | 3.88E-08 |
| gene-Led04484-sp3 | -3.467088136 | 0.0000572 |
| gene-Led04499-sp3 | 2.460229229 | 1.09E-20 |
| gene-Led04512-sp3 | 0.793236263 | 0.006357213 |
| gene-Led04519-sp3 | -1.415582826 | 0.00000197 |
| gene-Led04523-sp3 | 0.464246012 | 0.000863449 |
| gene-Led04557-sp3 | 0.733976923 | 0.004297177 |
| gene-Led04558-sp3 | 1.488731413 | 0.00000101 |
| gene-Led04559-sp3 | 0.573067001 | 0.008924978 |
| gene-Led04560-sp3 | 0.782125204 | 0.0000173 |
| gene-Led04562-sp3 | -1.006342857 | 0.000289771 |
| gene-Led04568-sp3 | -1.209342458 | 0.0000119 |
| gene-Led04569-sp3 | 0.639860371 | 0.0000106 |
| gene-Led04592-sp3 | -1.02537851 | 0.000954688 |
| gene-Led04602-sp3 | 0.66294267 | 0.004351122 |
| gene-Led04604-sp3 | 1.682900728 | 0.0000635 |
| gene-Led04617-sp3 | -3.674471279 | 3.28E-18 |
| gene-Led04642-sp3 | -0.97222246 | 0.000104271 |
| gene-Led04649-sp3 | -1.969256384 | 0.000000602 |
| gene-Led04656-sp3 | 0.51091539 | 0.006291579 |
| gene-Led04663-sp3 | 1.006664771 | 0.0000459 |
| gene-Led04680-sp3 | 0.821290976 | 0.009199789 |

| | | |
|---|---|---|
| gene-Led04683-sp3 | -5.982927662 | 1.32E-21 |
| gene-Led04704-sp3 | -0.63547437 | 0.008922933 |
| gene-Led04755-sp3 | 1.032236914 | 3.03E-09 |
| gene-Led04763-sp3 | 0.955174418 | 0.000117837 |
| gene-Led04764-sp3 | -0.680103832 | 0.000672889 |
| gene-Led04771-sp3 | 1.294518875 | 0.00000718 |
| gene-Led04796-sp3 | -2.333665332 | 1.06E-08 |
| gene-Led04806-sp3 | -2.601382337 | 3.43E-10 |
| gene-Led04816-sp3 | -1.763378613 | 8.48E-08 |
| gene-Led04817-sp3 | -1.082779394 | 0.000000867 |
| gene-Led04817-sp3 | -0.809490447 | 0.0000588 |
| gene-Led04882-sp3 | -0.678402457 | 0.001138184 |
| gene-Led04883-sp3 | -0.915106157 | 0.004004534 |
| gene-Led04911-sp3 | -1.438602984 | 0.000918898 |
| gene-Led04921-sp3 | 0.564930092 | 0.001346555 |
| gene-Led04942-sp3 | -0.548131423 | 0.009351982 |
| gene-Led04945-sp3 | -1.812424288 | 2.49E-17 |
| gene-Led04946-sp3 | -1.532034828 | 0.0000132 |
| gene-Led04948-sp3 | 0.997711897 | 0.003628956 |
| gene-Led04949-sp3 | -1.285108146 | 2.36E-10 |
| gene-Led04950-sp3 | -0.867665036 | 0.00000813 |
| gene-Led04955-sp3 | 1.223909335 | 8.1E-09 |
| gene-Led04958-sp3 | 2.859878983 | 5.23E-22 |
| gene-Led04959-sp3 | 1.48442019 | 0.000266292 |
| gene-Led04960-sp3 | 1.424636467 | 0.000000796 |
| gene-Led04968-sp3 | 0.466585652 | 0.008657436 |
| gene-Led04975-sp3 | -1.041478311 | 0.000000565 |
| gene-Led04976-sp3 | -1.661733732 | 0.000262847 |
| gene-Led04982-sp3 | 0.547392805 | 0.001289219 |
| gene-Led05015-sp3 | -2.086354238 | 0.0048883 |
| gene-Led05017-sp3 | 1.613176714 | 0.000000594 |
| gene-Led05022-sp3 | -4.466924298 | 4.95E-21 |
| gene-Led05031-sp3 | -0.996441325 | 0.002767612 |
| gene-Led05036-sp3 | -1.601487472 | 0.00000482 |
| gene-Led05039-sp3 | -1.865526841 | 0.000000356 |
| gene-Led05049-sp3 | 0.891728313 | 0.000523434 |
| gene-Led05054-sp3 | 2.418138246 | 0.003605228 |
| gene-Led05056-sp3 | 1.401931148 | 0.00000701 |
| gene-Led05062-sp3 | -1.888457517 | 0.0000069 |
| gene-Led05063-sp3 | -2.514087088 | 3.07E-08 |
| gene-Led05068-sp3 | 1.624337249 | 9.48E-09 |
| gene-Led05069-sp3 | 0.55652376 | 0.004951833 |
| gene-Led05082-sp3 | 0.847387794 | 0.004242001 |
| gene-Led05085-sp3 | 0.455846772 | 0.003954807 |

| | | |
|---|---|---|
| gene-Led05087-sp3 | 2.194060195 | 0.00000631 |
| gene-Led05094-sp3 | 0.894669981 | 0.001684767 |
| gene-Led05095-sp3 | -1.630659298 | 1.73E-11 |
| gene-Led05098-sp3 | 0.474746129 | 0.002849504 |
| gene-Led05099-sp3 | 0.617071819 | 0.00295085 |
| gene-Led05105-sp3 | -1.426905577 | 0.0000122 |
| gene-Led05116-sp3 | 0.494253925 | 0.00424867 |
| gene-Led05123-sp3 | 1.945526725 | 3.09E-08 |
| gene-Led05126-sp3 | -2.227370315 | 8.96E-08 |
| gene-Led05130-sp3 | -2.715465641 | 6.73E-26 |
| gene-Led05133-sp3 | -3.956665623 | 2.03E-12 |
| gene-Led05139-sp3 | 1.404106716 | 0.000535701 |
| gene-Led05144-sp3 | 0.932542509 | 0.000000204 |
| gene-Led05169-sp3 | 0.864317651 | 0.006288025 |
| gene-Led05176-sp3 | 1.172202398 | 0.001623494 |
| gene-Led05203-sp3 | -1.114057625 | 6.44E-10 |
| gene-Led05207-sp3 | -2.898823759 | 4.14E-12 |
| gene-Led05223-sp3 | 0.667199777 | 0.002571661 |
| gene-Led05224-sp3 | -1.158927921 | 0.000318752 |
| gene-Led05230-sp3 | -0.77698188 | 0.00000821 |
| gene-Led05248-sp3 | 0.635646629 | 0.00098636 |
| gene-Led05265-sp3 | -0.686722214 | 0.000477782 |
| gene-Led05273-sp3 | 0.879376737 | 0.000129387 |
| gene-Led05285-sp3 | 1.300072701 | 0.000422039 |
| gene-Led05300-sp3 | 1.088170104 | 0.00000444 |
| gene-Led05321-sp3 | 0.554666742 | 0.000323102 |
| gene-Led05329-sp3 | -0.821370618 | 0.0000216 |
| gene-Led05331-sp3 | 0.598220351 | 0.001584572 |
| gene-Led05340-sp3 | 1.097897215 | 1.1E-14 |
| gene-Led05388-sp3 | 0.409113392 | 0.008890476 |
| gene-Led05402-sp3 | 0.608570459 | 0.001318521 |
| gene-Led05458-sp3 | 0.773095418 | 0.000023 |
| gene-Led05462-sp3 | -0.629670213 | 0.001069384 |
| gene-Led05539-sp3 | 1.45544501 | 0.000022 |
| gene-Led05540-sp3 | 0.814647441 | 0.000148788 |
| gene-Led05558-sp3 | -0.802765348 | 0.000688884 |
| gene-Led05561-sp3 | -1.126559985 | 0.000704178 |
| gene-Led05570-sp3 | 2.704449644 | 9.39E-14 |
| gene-Led05578-sp3 | 1.179002146 | 0.000476917 |
| gene-Led05587-sp3 | -0.840431117 | 0.004213177 |
| gene-Led05589-sp3 | -0.687071721 | 0.001461136 |
| gene-Led05591-sp3 | 0.734574212 | 0.000885502 |
| gene-Led05601-sp3 | 0.383722865 | 0.007158071 |
| gene-Led05631-sp3 | 1.282152639 | 0.00012881 |

| | | |
|---|---|---|
| gene-Led05652-sp3 | 0.709593396 | 0.0000145 |
| gene-Led05658-sp3 | -1.470457959 | 0.000000999 |
| gene-Led05669-sp3 | 0.442815306 | 0.00137781 |
| gene-Led05686-sp3 | 0.556351796 | 0.001650508 |
| gene-Led05693-sp3 | -1.456127933 | 6.09E-09 |
| gene-Led05698-sp3 | -2.033052994 | 0.00000931 |
| gene-Led05718-sp3 | -1.75172034 | 0.0000256 |
| gene-Led05719-sp3 | 0.772275416 | 0.0000923 |
| gene-Led05728-sp3 | 1.933369396 | 1.01E-12 |
| gene-Led05740-sp3 | -0.658036635 | 0.004628869 |
| gene-Led05743-sp3 | 0.74519546 | 0.005809117 |
| gene-Led05747-sp3 | -0.891149763 | 0.000598257 |
| gene-Led05770-sp3 | -1.651551911 | 2.94E-09 |
| gene-Led05778-sp3 | 0.455445401 | 0.002109068 |
| gene-Led05789-sp3 | 1.049702052 | 0.000000116 |
| gene-Led05795-sp3 | 1.434121475 | 0.002074984 |
| gene-Led05802-sp3 | 0.595771808 | 0.000105026 |
| gene-Led05807-sp3 | 0.684662426 | 0.000590346 |
| gene-Led05837-sp3 | 0.558040233 | 0.0004319 |
| gene-Led05862-sp3 | -0.748270362 | 0.000213294 |
| gene-Led05864-sp3 | 0.777008309 | 2.19E-08 |
| gene-Led05881-sp3 | 1.292327788 | 0.000000143 |
| gene-Led05883-sp3 | -0.496346298 | 0.007580422 |
| gene-Led05887-sp3 | -0.680493927 | 0.008706897 |
| gene-Led05902-sp3 | 1.19803569 | 0.000151696 |
| gene-Led05916-sp3 | -0.681784437 | 0.000440792 |
| gene-Led05919-sp3 | 0.734337187 | 0.004299181 |
| gene-Led05934-sp3 | 0.869511602 | 0.000361969 |
| gene-Led05940-sp3 | -1.077742044 | 0.000000023 |
| gene-Led05961-sp3 | 3.220563648 | 0.0000755 |
| gene-Led05983-sp3 | 2.792105397 | 0.00098603 |
| gene-Led05986-sp3 | 1.066081212 | 0.000056 |
| gene-Led06007-sp3 | 0.843475918 | 0.004031366 |
| gene-Led06015-sp3 | 1.218585517 | 0.006462665 |
| gene-Led06029-sp3 | 1.563870501 | 0.000000179 |
| gene-Led06031-sp3 | -0.902259222 | 0.0000771 |
| gene-Led06046-sp3 | 4.022083172 | 0.000581687 |
| gene-Led06051-sp3 | 0.747591384 | 0.0016977 |
| gene-Led06053-sp3 | 1.720815668 | 0.008824236 |
| gene-Led06055-sp3 | 0.962695007 | 0.000374534 |
| gene-Led06075-sp3 | 1.783943428 | 0.0000118 |
| gene-Led06084-sp3 | 0.696806289 | 0.003919103 |
| gene-Led06118-sp3 | 0.848787878 | 0.003285872 |
| gene-Led06124-sp3 | -1.574809382 | 4.33E-13 |

| | | |
|---|---|---|
| gene-Led06138-sp3 | -1.016707804 | 0.000000172 |
| gene-Led06142-sp3 | -0.60869923 | 0.001925157 |
| gene-Led06148-sp3 | -2.173115303 | 0.00401513 |
| gene-Led06149-sp3 | -2.868235991 | 1.17E-28 |
| gene-Led06150-sp3 | -2.631309485 | 1.34E-24 |
| gene-Led06162-sp3 | -3.450613155 | 4.84E-08 |
| gene-Led06165-sp3 | 1.327094296 | 2.27E-10 |
| gene-Led06217-sp3 | 2.056331501 | 0.000387045 |
| gene-Led06227-sp3 | 1.591223261 | 0.0000502 |
| gene-Led06254-sp3 | 0.458584735 | 0.003159346 |
| gene-Led06261-sp3 | -1.286712258 | 0.0000914 |
| gene-Led06265-sp3 | -1.430851636 | 0.00000177 |
| gene-Led06315-sp3 | -1.122450237 | 0.000339705 |
| gene-Led06338-sp3 | 3.431992586 | 1.01E-41 |
| gene-Led06347-sp3 | 1.858600181 | 3.9E-15 |
| gene-Led06349-sp3 | 1.135899608 | 0.001087584 |
| gene-Led06356-sp3 | 1.315007354 | 0.003288051 |
| gene-Led06363-sp3 | 0.67930194 | 0.000116149 |
| gene-Led06365-sp3 | 0.606565427 | 0.003713694 |
| gene-Led06367-sp3 | -3.222968219 | 8.04E-08 |
| gene-Led06391-sp3 | -4.945684399 | 2.6E-72 |
| gene-Led06391-sp3 | -4.773953532 | 2.03E-39 |
| gene-Led06391-sp3 | -4.17205534 | 0.0000357 |
| gene-Led06420-sp3 | 1.069383808 | 0.008599547 |
| gene-Led06448-sp3 | 0.515553461 | 0.009331302 |
| gene-Led06456-sp3 | -1.022157985 | 0.0000288 |
| gene-Led06464-sp3 | 1.244272026 | 0.0000276 |
| gene-Led06477-sp3 | -1.100386635 | 0.000827678 |
| gene-Led06479-sp3 | -1.072223464 | 0.004727081 |
| gene-Led06483-sp3 | -0.892548211 | 0.00000382 |
| gene-Led06492-sp3 | -0.859369991 | 0.000262391 |
| gene-Led06514-sp3 | -1.469575417 | 0.00000834 |
| gene-Led06518-sp3 | 0.539628567 | 0.002235852 |
| gene-Led06524-sp3 | 0.933119576 | 0.00000214 |
| gene-Led06526-sp3 | 0.853561246 | 0.000103752 |
| gene-Led06530-sp3 | 2.424252364 | 1.69E-14 |
| gene-Led06546-sp3 | 4.023459759 | 2.43E-40 |
| gene-Led06547-sp3 | -1.675402492 | 0.0000459 |
| gene-Led06561-sp3 | -0.462806282 | 0.009111346 |
| gene-Led06582-sp3 | -2.372080926 | 2.71E-21 |
| gene-Led06598-sp3 | 0.94078762 | 0.00000145 |
| gene-Led06602-sp3 | 0.758809851 | 0.000124765 |
| gene-Led06603-sp3 | -1.692773393 | 0.000000825 |
| gene-Led06637-sp3 | 0.644255282 | 0.00018522 |

| | | |
|---|---|---|
| gene-Led06642-sp3 | 1.683181773 | 0.000000154 |
| gene-Led06646-sp3 | 0.748680605 | 0.000003 |
| gene-Led06661-sp3 | 0.528333511 | 0.003226647 |
| gene-Led06674-sp3 | 0.691316992 | 0.000372868 |
| gene-Led06679-sp3 | -0.947865143 | 0.0000963 |
| gene-Led06732-sp3 | 2.385085397 | 0.0000275 |
| gene-Led06754-sp3 | -0.807079629 | 0.002804069 |
| gene-Led06772-sp3 | 1.510247575 | 0.00000165 |
| gene-Led06782-sp3 | 1.391141785 | 0.00000896 |
| gene-Led06798-sp3 | -1.115896302 | 0.000757316 |
| gene-Led06808-sp3 | 0.764158539 | 0.000293002 |
| gene-Led06827-sp3 | 1.750280038 | 0.000091 |
| gene-Led06842-sp3 | 0.610300866 | 0.003066184 |
| gene-Led06846-sp3 | 1.012724766 | 0.0000022 |
| gene-Led06848-sp3 | 2.758587885 | 0.000003 |
| gene-Led06850-sp3 | 3.664787297 | 8.95E-16 |
| gene-Led06873-sp3 | -1.170959154 | 2.47E-09 |
| gene-Led06888-sp3 | 3.005360365 | 0.000000733 |
| gene-Led06911-sp3 | 1.096616341 | 0.002751702 |
| gene-Led06929-sp3 | -0.646413494 | 0.000526346 |
| gene-Led06931-sp3 | 1.188828358 | 0.000102499 |
| gene-Led06950-sp3 | 0.640321562 | 0.001872095 |
| gene-Led06957-sp3 | 1.396852481 | 1.01E-08 |
| gene-Led06994-sp3 | 0.83106801 | 0.003024078 |
| gene-Led06998-sp3 | -1.272445604 | 0.000000371 |
| gene-Led07008-sp3 | -1.441916232 | 0.000000618 |
| gene-Led07019-sp3 | 1.153848334 | 1.95E-08 |
| gene-Led07041-sp3 | -1.133348363 | 0.0000392 |
| gene-Led07043-sp3 | 1.88836735 | 0.00000373 |
| gene-Led07089-sp3 | 1.048617488 | 0.000000685 |
| gene-Led07092-sp3 | 1.344869941 | 0.000107334 |
| gene-Led07100-sp3 | -2.491813055 | 4.4E-10 |
| gene-Led07102-sp3 | -3.642714145 | 2.59E-40 |
| gene-Led07104-sp3 | -0.770907789 | 0.000149597 |
| gene-Led07136-sp3 | -0.941320214 | 0.003405433 |
| gene-Led07143-sp3 | 0.870648362 | 0.000197954 |
| gene-Led07148-sp3 | 3.338820656 | 3.33E-11 |
| gene-Led07155-sp3 | -1.606228939 | 0.000000045 |
| gene-Led07161-sp3 | -1.632109357 | 0.00000429 |
| gene-Led07180-sp3 | 2.096717498 | 1.47E-08 |
| gene-Led07183-sp3 | 6.242175355 | 5.41E-08 |
| gene-Led07201-sp3 | -1.57330193 | 0.000013 |
| gene-Led07204-sp3 | 2.181911974 | 7.26E-13 |
| gene-Led07205-sp3 | 0.728660597 | 0.008307348 |

| | | |
|---|---|---|
| gene-Led07210-sp3 | 1.215557569 | 0.000000362 |
| gene-Led07217-sp3 | 1.642073143 | 1.59E-08 |
| gene-Led07218-sp3 | 1.27886016 | 0.000000443 |
| gene-Led07222-sp3 | 1.109690244 | 0.0000152 |
| gene-Led07224-sp3 | 1.794544176 | 2.31E-11 |
| gene-Led07234-sp3 | -0.58274189 | 0.003169862 |
| gene-Led07275-sp3 | 0.838970103 | 8.86E-08 |
| gene-Led07300-sp3 | 1.487864397 | 2.56E-12 |
| gene-Led07306-sp3 | 1.680078701 | 0.00502088 |
| gene-Led07313-sp3 | -0.774346664 | 0.007054276 |
| gene-Led07334-sp3 | 0.128657559 | 0.000215389 |
| gene-Led07334-sp3 | -0.523186855 | 0.003545859 |
| gene-Led07345-sp3 | 2.318045092 | 3.48E-14 |
| gene-Led07360-sp3 | 1.996931081 | 0.0000154 |
| gene-Led07362-sp3 | 0.493941828 | 0.00437805 |
| gene-Led07388-sp3 | 0.665042084 | 0.002410772 |
| gene-Led07391-sp3 | -1.538331787 | 0.000000555 |
| gene-Led07397-sp3 | 0.669624467 | 0.00296859 |
| gene-Led07404-sp3 | -2.739799168 | 1.63E-09 |
| gene-Led07405-sp3 | 0.488794225 | 0.005985058 |
| gene-Led07419-sp3 | 0.840837628 | 0.000407095 |
| gene-Led07430-sp3 | 1.359903614 | 0.004995175 |
| gene-Led07465-sp3 | 0.678235526 | 0.000304844 |
| gene-Led07476-sp3 | 0.510421473 | 0.004515098 |
| gene-Led07486-sp3 | -0.452908454 | 0.005122716 |
| gene-Led07489-sp3 | 0.612634573 | 0.000405576 |
| gene-Led07495-sp3 | -0.632959361 | 0.000177117 |
| gene-Led07502-sp3 | 0.740018816 | 0.00004 |
| gene-Led07506-sp3 | -0.512730546 | 0.009567711 |
| gene-Led07517-sp3 | -0.509042969 | 0.008777401 |
| gene-Led07525-sp3 | -0.869807563 | 0.0000128 |
| gene-Led07526-sp3 | 2.994588086 | 0.00000145 |
| gene-Led07546-sp3 | 0.569270361 | 0.000972056 |
| gene-Led07568-sp3 | 0.474333271 | 0.006168021 |
| gene-Led07587-sp3 | -0.778944866 | 0.001483827 |
| gene-Led07592-sp3 | 2.469887673 | 2.43E-18 |
| gene-Led07595-sp3 | -4.614646538 | 7.79E-46 |
| gene-Led07596-sp3 | -0.991345016 | 0.001909197 |
| gene-Led07626-sp3 | 1.00587288 | 0.000000875 |
| gene-Led07637-sp3 | 0.524274854 | 0.003689528 |
| gene-Led07646-sp3 | -0.72268659 | 0.0000742 |
| gene-Led07695-sp3 | 0.783757475 | 0.00000414 |
| gene-Led07697-sp3 | 2.72157644 | 6.99E-11 |
| gene-Led07706-sp3 | 1.070513476 | 0.000907998 |

| | | |
|---|---|---|
| gene-Led07713-sp3 | -1.111116028 | 0.000215808 |
| gene-Led07718-sp3 | 0.509287459 | 0.007154559 |
| gene-Led07720-sp3 | 0.459338728 | 0.005092498 |
| gene-Led07738-sp3 | 0.675549738 | 0.000715637 |
| gene-Led07742-sp3 | 0.842675243 | 0.007680125 |
| gene-Led07744-sp3 | -2.113263991 | 0.0000178 |
| gene-Led07758-sp3 | 1.867503057 | 0.000167873 |
| gene-Led07765-sp3 | 0.50594749 | 0.005620196 |
| gene-Led07782-sp3 | -1.619623829 | 0.00000382 |
| gene-Led07788-sp3 | 0.796290365 | 0.0000689 |
| gene-Led07836-sp3 | 0.578015293 | 0.0012998 |
| gene-Led07845-sp3 | 1.034728172 | 0.000000929 |
| gene-Led07856-sp3 | 0.910623195 | 0.002349751 |
| gene-Led07861-sp3 | -0.617834978 | 0.004362134 |
| gene-Led07862-sp3 | 0.790267062 | 0.000000501 |
| gene-Led07864-sp3 | -0.715409245 | 0.001605802 |
| gene-Led07866-sp3 | 0.586877546 | 0.000668408 |
| gene-Led07898-sp3 | 0.768266732 | 0.00546115 |
| gene-Led07904-sp3 | -0.629914659 | 0.000864371 |
| gene-Led07908-sp3 | 1.243503896 | 0.00000686 |
| gene-Led07916-sp3 | 0.732743654 | 0.000103412 |
| gene-Led07917-sp3 | 1.519164244 | 0.000000789 |
| gene-Led07958-sp3 | -1.225315028 | 0.0000653 |
| gene-Led07979-sp3 | 0.692438668 | 0.000073 |
| gene-Led08009-sp3 | 0.443395154 | 0.005172368 |
| gene-Led08015-sp3 | 0.622782346 | 0.006435999 |
| gene-Led08026-sp3 | -1.706241564 | 0.0000592 |
| gene-Led08036-sp3 | 0.663124822 | 0.002337513 |
| gene-Led08044-sp3 | 0.386227522 | 0.006245175 |
| gene-Led08055-sp3 | 0.513333152 | 0.007810177 |
| gene-Led08072-sp3 | -1.142667823 | 0.001266076 |
| gene-Led08110-sp3 | 0.934672805 | 0.00000257 |
| gene-Led08112-sp3 | 0.524448826 | 0.005948932 |
| gene-Led08165-sp3 | 1.921514074 | 0.000297167 |
| gene-Led08168-sp3 | 1.937678585 | 0.000000305 |
| gene-Led08169-sp3 | 0.817662297 | 0.000116572 |
| gene-Led08177-sp3 | 1.028742074 | 0.007903234 |
| gene-Led08181-sp3 | 1.281513416 | 0.00033334 |
| gene-Led08184-sp3 | 2.053807066 | 5.61E-19 |
| gene-Led08187-sp3 | 2.120769366 | 5.32E-08 |
| gene-Led08195-sp3 | 1.653394542 | 0.002023439 |
| gene-Led08200-sp3 | 1.354151878 | 0.0000113 |
| gene-Led08203-sp3 | -0.521164408 | 0.00183929 |
| gene-Led08210-sp3 | 1.280328486 | 0.000000023 |

| | | |
|---|---|---|
| gene-Led08217-sp3 | -3.355995021 | 3.86E-20 |
| gene-Led08218-sp3 | -2.552982442 | 1.01E-16 |
| gene-Led08224-sp3 | 2.279154129 | 0.00000595 |
| gene-Led08238-sp3 | 1.710411227 | 0.000000173 |
| gene-Led08257-sp3 | -1.710447182 | 0.000013 |
| gene-Led08281-sp3 | 0.603022267 | 0.000407852 |
| gene-Led08389-sp3 | -0.915412961 | 0.0000507 |
| gene-Led08422-sp3 | -0.653204678 | 0.009898437 |
| gene-Led08431-sp3 | 0.843638578 | 0.00000124 |
| gene-Led08432-sp3 | 0.74076322 | 0.000202712 |
| gene-Led08448-sp3 | 1.773336691 | 8.83E-14 |
| gene-Led08460-sp3 | -3.487333834 | 6.28E-15 |
| gene-Led08464-sp3 | -0.868957262 | 0.009594897 |
| gene-Led08465-sp3 | -0.819437782 | 0.001731718 |
| gene-Led08483-sp3 | 2.416970745 | 0.000317048 |
| gene-Led08489-sp3 | 0.874103051 | 0.000195772 |
| gene-Led08508-sp3 | 0.555836667 | 0.000803582 |
| gene-Led08516-sp3 | 0.964048846 | 0.00000949 |
| gene-Led08569-sp3 | -1.548090347 | 0.000000129 |
| gene-Led08597-sp3 | -0.603388148 | 0.002932794 |
| gene-Led08608-sp3 | -2.844143693 | 0.0000269 |
| gene-Led08610-sp3 | 0.871039507 | 0.000589553 |
| gene-Led08622-sp3 | -3.814135825 | 6.02E-32 |
| gene-Led08624-sp3 | -5.330798719 | 1.25E-14 |
| gene-Led08625-sp3 | -2.507051345 | 6.57E-13 |
| gene-Led08636-sp3 | -1.832683793 | 2.2E-11 |
| gene-Led08645-sp3 | -1.59187156 | 0.00000405 |
| gene-Led08657-sp3 | 1.730032454 | 0.001840169 |
| gene-Led08658-sp3 | 1.990078029 | 0.00000353 |
| gene-Led08658-sp3 | 1.609766993 | 0.00300507 |
| gene-Led08659-sp3 | 1.084324857 | 0.000430766 |
| gene-Led08669-sp3 | 0.733786935 | 0.001108212 |
| gene-Led08682-sp3 | 1.558897969 | 0.000000254 |
| gene-Led08683-sp3 | 0.733496053 | 0.000181735 |
| gene-Led08690-sp3 | -1.584544563 | 0.000529951 |
| gene-Led08695-sp3 | -0.817654285 | 0.007386204 |
| gene-Led08706-sp3 | -1.824012242 | 8.52E-08 |
| gene-Led08715-sp3 | -0.725313269 | 0.001880887 |
| gene-Led08733-sp3 | -0.90064897 | 0.000251502 |
| gene-Led08734-sp3 | -3.737501512 | 5.61E-17 |
| gene-Led08742-sp3 | -1.360063994 | 0.001922743 |
| gene-Led08744-sp3 | 1.245249578 | 0.00066553 |
| gene-Led08745-sp3 | -0.496075956 | 0.002694418 |
| gene-Led08757-sp3 | -1.383802018 | 1.36E-08 |

| | | |
|---|---|---|
| gene-Led08786-sp3 | -0.603385675 | 0.005545343 |
| gene-Led08827-sp3 | -0.919359298 | 0.00000026 |
| gene-Led08839-sp3 | -0.967640346 | 0.004680694 |
| gene-Led08840-sp3 | -1.161902218 | 0.002718315 |
| gene-Led08870-sp3 | 0.806498257 | 0.007904412 |
| gene-Led08877-sp3 | -1.081518213 | 3.12E-09 |
| gene-Led08878-sp3 | 0.73197062 | 0.00000581 |
| gene-Led08885-sp3 | -0.749735713 | 0.000920683 |
| gene-Led08887-sp3 | 0.490775469 | 0.000285214 |
| gene-Led08896-sp3 | -1.40195914 | 0.000207663 |
| gene-Led08924-sp3 | 1.495757833 | 0.00000895 |
| gene-Led08945-sp3 | -1.073319547 | 0.004065415 |
| gene-Led08999-sp3 | -1.337951859 | 8.07E-10 |
| gene-Led09023-sp3 | 0.814654447 | 0.000650674 |
| gene-Led09039-sp3 | 0.760096214 | 0.003999075 |
| gene-Led09044-sp3 | 0.621256096 | 0.000979941 |
| gene-Led09054-sp3 | -0.823001894 | 0.00000345 |
| gene-Led09059-sp3 | -1.045520738 | 0.00000151 |
| gene-Led09068-sp3 | 0.935063182 | 0.000166464 |
| gene-Led09096-sp3 | 0.993164611 | 5.52E-08 |
| gene-Led09126-sp3 | -1.072071279 | 0.0000111 |
| gene-Led09145-sp3 | 0.745676062 | 0.005654548 |
| gene-Led09160-sp3 | -0.711999026 | 0.006030245 |
| gene-Led09161-sp3 | -1.676816424 | 0.000000995 |
| gene-Led09163-sp3 | 0.55660616 | 0.001302742 |
| gene-Led09175-sp3 | 0.922090082 | 0.0000111 |
| gene-Led09177-sp3 | 1.712560791 | 0.001941961 |
| gene-Led09201-sp3 | 0.878169967 | 0.0000902 |
| gene-Led09207-sp3 | 0.557577492 | 0.001014437 |
| gene-Led09216-sp3 | 0.542650894 | 0.003422605 |
| gene-Led09248-sp3 | 0.818305533 | 0.0000622 |
| gene-Led09252-sp3 | 0.696485457 | 0.001037738 |
| gene-Led09255-sp3 | 0.731777497 | 0.004017787 |
| gene-Led09259-sp3 | 0.918553533 | 0.00153992 |
| gene-Led09264-sp3 | 0.518476695 | 0.002419869 |
| gene-Led09270-sp3 | -1.310725203 | 0.00000215 |
| gene-Led09275-sp3 | 0.586283407 | 0.000603 |
| gene-Led09277-sp3 | -0.989398628 | 0.002647685 |
| gene-Led09291-sp3 | 0.510267001 | 0.003324884 |
| gene-Led09311-sp3 | 1.009432242 | 0.004931147 |
| gene-Led09321-sp3 | 0.628582576 | 0.009480953 |
| gene-Led09364-sp3 | -0.994858995 | 0.001499443 |
| gene-Led09371-sp3 | 0.705443616 | 0.0000105 |
| gene-Led09374-sp3 | 1.56212788 | 0.003477128 |

| | | |
|---|---|---|
| gene-Led09378-sp3 | 2.178915299 | 0.0000463 |
| gene-Led09386-sp3 | -1.747355696 | 0.001410528 |
| gene-Led09411-sp3 | -0.720406537 | 0.0078772 |
| gene-Led09418-sp3 | 1.489727659 | 7.53E-09 |
| gene-Led09420-sp3 | -1.046495899 | 0.000894303 |
| gene-Led09424-sp3 | 0.949400583 | 0.000326835 |
| gene-Led09430-sp3 | 1.016733314 | 8.74E-11 |
| gene-Led09431-sp3 | 1.051372756 | 0.000000642 |
| gene-Led09437-sp3 | 0.929519439 | 4.46E-08 |
| gene-Led09438-sp3 | 0.867158461 | 4.73E-10 |
| gene-Led09446-sp3 | 0.736590424 | 0.001590482 |
| gene-Led09450-sp3 | -0.79040274 | 0.000474932 |
| gene-Led09462-sp3 | -1.459485228 | 0.001583837 |
| gene-Led09479-sp3 | -1.027165312 | 0.000142917 |
| gene-Led09499-sp3 | 0.818219897 | 0.001337484 |
| gene-Led09516-sp3 | 0.68948712 | 0.000802499 |
| gene-Led09519-sp3 | -1.187218291 | 0.000275717 |
| gene-Led09543-sp3 | 0.846679842 | 0.004514698 |
| gene-Led09557-sp3 | -1.743569143 | 4.74E-08 |
| gene-Led09577-sp3 | -1.904626272 | 0.00000191 |
| gene-Led09598-sp3 | -0.579268867 | 0.00672637 |
| gene-Led09604-sp3 | 0.769204748 | 0.0000521 |
| gene-Led09616-sp3 | -1.62888998 | 0.00000172 |
| gene-Led09617-sp3 | -1.442619109 | 2.55E-09 |
| gene-Led09635-sp3 | 1.930178167 | 0.002697809 |
| gene-Led09680-sp3 | 0.847464558 | 0.000437673 |
| gene-Led09685-sp3 | 1.138089794 | 0.000740811 |
| gene-Led09696-sp3 | -0.987763444 | 1.36E-09 |
| gene-Led09711-sp3 | 0.74546439 | 0.006733237 |
| gene-Led09714-sp3 | 0.720417198 | 0.00674793 |
| gene-Led09763-sp3 | 0.691473974 | 0.00000954 |
| gene-Led09806-sp3 | 1.930821767 | 0.001788275 |
| gene-Led09812-sp3 | -1.108262202 | 0.000522916 |
| gene-Led09813-sp3 | -1.092733325 | 0.000245312 |
| gene-Led09825-sp3 | 0.690341381 | 0.000162536 |
| gene-Led09836-sp3 | 1.375267198 | 0.0000317 |
| gene-Led09839-sp3 | -0.448483221 | 0.005032073 |
| gene-Led09846-sp3 | -0.611059961 | 0.000774882 |
| gene-Led09866-sp3 | 0.531145498 | 0.003838267 |
| gene-Led09880-sp3 | -0.679028298 | 0.00711465 |
| gene-Led09885-sp3 | -1.070847551 | 0.0000102 |
| gene-Led09897-sp3 | 0.992474917 | 0.005676677 |
| gene-Led09997-sp3 | 1.997267878 | 0.002210869 |
| gene-Led10005-sp3 | 1.797943734 | 0.000107839 |

| | | |
|---|---|---|
| gene-Led10006-sp3 | 2.245954928 | 3.76E-09 |
| gene-Led10016-sp3 | 0.539590817 | 0.004264417 |
| gene-Led10034-sp3 | -0.660377158 | 0.000531656 |
| gene-Led10046-sp3 | -1.119399951 | 0.00000514 |
| gene-Led10050-sp3 | 0.972868077 | 0.0000134 |
| gene-Led10065-sp3 | 1.08180845 | 0.0000128 |
| gene-Led10088-sp3 | 0.568664605 | 0.00616023 |
| gene-Led10099-sp3 | -1.386516351 | 0.0000743 |
| gene-Led10102-sp3 | 0.490171919 | 0.002828294 |
| gene-Led10108-sp3 | 2.128231983 | 0.002663661 |
| gene-Led10111-sp3 | 0.61824521 | 0.003894154 |
| gene-Led10121-sp3 | 0.88255838 | 0.001156467 |
| gene-Led10122-sp3 | 0.573536023 | 0.003747512 |
| gene-Led10123-sp3 | 0.94807715 | 5.62E-08 |
| gene-Led10145-sp3 | -0.543435016 | 0.000722332 |
| gene-Led10158-sp3 | -0.502124982 | 0.00620474 |
| gene-Led10163-sp3 | 0.948224492 | 0.002190991 |
| gene-Led10175-sp3 | -1.537823967 | 0.001534419 |
| gene-Led10175-sp3 | -5.580098194 | 0.0000471 |
| gene-Led10176-sp3 | 0.650122364 | 0.001065526 |
| gene-Led10184-sp3 | 1.845958219 | 2.99E-10 |
| gene-Led10205-sp3 | 0.534240356 | 0.00126666 |
| gene-Led10225-sp3 | 0.817101405 | 0.0000784 |
| gene-Led10228-sp3 | -1.077001623 | 0.0000374 |
| gene-Led10235-sp3 | -3.813696675 | 0.000000721 |
| gene-Led10238-sp3 | -1.71812405 | 0.000294952 |
| gene-Led10246-sp3 | 1.667812723 | 1.32E-21 |
| gene-Led10299-sp3 | -0.915582021 | 0.004298376 |
| gene-Led10300-sp3 | -1.269870761 | 0.000269646 |
| gene-Led10303-sp3 | -1.212482566 | 0.000748245 |
| gene-Led10312-sp3 | 2.077856978 | 1.17E-08 |
| gene-Led10316-sp3 | 2.054330269 | 2.19E-09 |
| gene-Led10321-sp3 | -1.565266231 | 0.000305829 |
| gene-Led10322-sp3 | 2.137182068 | 0.0000136 |
| gene-Led10323-sp3 | -1.77792436 | 0.000000718 |
| gene-Led10329-sp3 | 0.84437723 | 0.001916538 |
| gene-Led10330-sp3 | 0.718048471 | 0.00304364 |
| gene-Led10340-sp3 | 1.443778908 | 0.0000225 |
| gene-Led10344-sp3 | -0.963329186 | 0.001566625 |
| gene-Led10352-sp3 | -1.517839263 | 0.001658192 |
| gene-Led10354-sp3 | 0.890641312 | 0.001704489 |
| gene-Led10375-sp3 | -0.818966374 | 0.0000481 |
| gene-Led10376-sp3 | -1.219896012 | 0.00000759 |
| gene-Led10469-sp3 | 0.420210753 | 0.007031605 |

| | | |
|---|---|---|
| gene-Led10477-sp3 | 1.405193441 | 0.000132932 |
| gene-Led10479-sp3 | 2.442586074 | 5.32E-15 |
| gene-Led10482-sp3 | -1.180574007 | 0.000867451 |
| gene-Led10483-sp3 | -3.103696063 | 5.42E-17 |
| gene-Led10484-sp3 | -2.80759502 | 1.85E-26 |
| gene-Led10490-sp3 | -1.601503273 | 0.000000307 |
| gene-Led10492-sp3 | -1.260024801 | 2.73E-08 |
| gene-Led10526-sp3 | -1.931456989 | 5.58E-14 |
| gene-Led10530-sp3 | 0.841469358 | 0.000482478 |
| gene-Led10531-sp3 | -1.998705187 | 7.36E-19 |
| gene-Led10551-sp3 | 1.276397662 | 0.005180956 |
| gene-Led10555-sp3 | -2.965314897 | 1.47E-23 |
| gene-Led10567-sp3 | 0.835139993 | 0.000370752 |
| gene-Led10581-sp3 | 2.789020595 | 0.0000852 |
| gene-Led10588-sp3 | 0.86948554 | 0.000000662 |
| gene-Led10596-sp3 | -0.945078276 | 0.006594021 |
| gene-Led10615-sp3 | -0.37750264 | 0.009932571 |
| gene-Led10621-sp3 | 2.449999751 | 0.0000148 |
| gene-Led10627-sp3 | -0.715301562 | 0.001296543 |
| gene-Led10631-sp3 | 0.566899955 | 0.003096593 |
| gene-Led10639-sp3 | 0.93694814 | 0.000772787 |
| gene-Led10643-sp3 | 0.775801923 | 0.000087 |
| gene-Led10683-sp3 | -0.706799225 | 0.005199946 |
| gene-Led10690-sp3 | -1.269800064 | 4.13E-08 |
| gene-Led10693-sp3 | 0.965337187 | 0.001975686 |
| gene-Led10728-sp3 | -0.531004941 | 0.001569526 |
| gene-Led10733-sp3 | 0.584706006 | 0.004264479 |
| gene-Led10735-sp3 | 1.108950642 | 1.34E-08 |
| gene-Led10745-sp3 | 0.448573589 | 0.009507922 |
| gene-Led10763-sp3 | -0.877674758 | 0.0000965 |
| gene-Led10765-sp3 | -0.805240852 | 0.00000711 |
| gene-Led10768-sp3 | 0.750790663 | 0.000369029 |
| gene-Led10771-sp3 | -1.026964344 | 0.0000117 |
| gene-Led10800-sp3 | -1.794501518 | 0.003013945 |
| gene-Led10808-sp3 | -2.010170851 | 0.0000398 |
| gene-Led10875-sp3 | 1.212402527 | 5.92E-08 |
| gene-Led10879-sp3 | 0.728034418 | 0.0000699 |
| gene-Led10897-sp3 | 1.487026698 | 0.000000381 |
| gene-Led10913-sp3 | 2.653673574 | 0.0000402 |
| gene-Led10925-sp3 | 1.082347099 | 0.0000101 |
| gene-Led10927-sp3 | 2.247854762 | 2.58E-08 |
| gene-Led10936-sp3 | 0.600265851 | 0.004104367 |
| gene-Led10998-sp3 | 0.96203441 | 0.0000659 |
| gene-Led11014-sp3 | 1.067653057 | 1.06E-08 |

| | | |
|---|---|---|
| gene-Led11016-sp3 | -0.92899682 | 0.000298742 |
| gene-Led11023-sp3 | 1.766167341 | 0.000123704 |
| gene-Led11048-sp3 | -1.673306805 | 0.002352296 |
| gene-Led11071-sp3 | -2.193064264 | 0.000372525 |
| gene-Led11086-sp3 | 0.840951646 | 0.00796871 |
| gene-Led11091-sp3 | 1.227972556 | 0.0000793 |
| gene-Led11122-sp3 | -4.466924298 | 4.95E-21 |
| gene-Led11139-sp3 | 1.107791806 | 0.000046 |
| gene-Led11182-sp3 | -0.8172673 | 0.0000117 |
| gene-Led11190-sp3 | 0.533848916 | 0.001541775 |
| gene-Led11194-sp3 | 1.901426183 | 0.000368429 |
| gene-Led11203-sp3 | -2.566750245 | 3.9E-13 |
| gene-Led11211-sp3 | -1.206727334 | 0.00000244 |
| gene-Led11212-sp3 | 1.069383808 | 0.008599547 |
| gene-Led11234-sp3 | 3.049616694 | 7.47E-23 |
| gene-Led11235-sp3 | 1.108462569 | 0.000563881 |
| gene-Led11264-sp3 | 1.105317448 | 0.000452148 |
| gene-Led11282-sp3 | 2.805171565 | 6.18E-20 |
| gene-Led11319-sp3 | 1.015954252 | 0.000120327 |
| gene-Led11377-sp3 | 0.619572084 | 0.004926146 |
| gene-Led11436-sp3 | 0.769204748 | 0.0000521 |
| gene-Led11455-sp3 | -0.973883168 | 0.003755948 |

**Table S5 DEG lists of LeHH24h vs LeHH3h**

| gene | log2FoldChange | pvalue |
|---|---|---|
| gene-Led00032-sp3 | -1.716464558 | 0.000106358 |
| gene-Led00043-sp3 | -0.997967164 | 0.002817814 |
| gene-Led00044-sp3 | -1.063860636 | 0.001480089 |
| gene-Led00074-sp3 | 1.355450924 | 0.003968985 |
| gene-Led00092-sp3 | 0.614969736 | 0.002869433 |
| gene-Led00095-sp3 | -1.203303645 | 7.05234E-06 |
| gene-Led00117-sp3 | 0.590025564 | 0.008381992 |
| gene-Led00122-sp3 | 1.021275877 | 0.003341465 |
| gene-Led00127-sp3 | -2.755919908 | 3.73204E-23 |
| gene-Led00129-sp3 | -0.562019629 | 0.000615504 |
| gene-Led00130-sp3 | -2.341640867 | 1.25128E-06 |
| gene-Led00165-sp3 | -0.861095482 | 5.38988E-05 |
| gene-Led00180-sp3 | -0.719376102 | 0.003023219 |
| gene-Led00196-sp3 | -0.871278247 | 0.003732149 |
| gene-Led00211-sp3 | 1.021686677 | 0.000178454 |
| gene-Led00241-sp3 | -0.491194818 | 0.004347572 |
| gene-Led00256-sp3 | 1.137215765 | 0.000350336 |
| gene-Led00287-sp3 | -1.099243708 | 6.81418E-07 |
| gene-Led00312-sp3 | 1.616370121 | 7.60418E-05 |
| gene-Led00336-sp3 | -0.775081374 | 0.001745396 |
| gene-Led00345-sp3 | 2.549400749 | 6.60188E-06 |
| gene-Led00395-sp3 | -1.255702567 | 9.64569E-05 |
| gene-Led00446-sp3 | -0.792286403 | 4.64172E-07 |
| gene-Led00452-sp3 | -0.63593129 | 0.001574232 |
| gene-Led00457-sp3 | 0.625600054 | 0.009395371 |
| gene-Led00473-sp3 | 1.014348318 | 0.004620633 |
| gene-Led00481-sp3 | -0.607752819 | 0.002793567 |
| gene-Led00484-sp3 | -0.505167619 | 0.002935841 |
| gene-Led00487-sp3 | -0.492924462 | 0.003703391 |
| gene-Led00488-sp3 | -0.634621425 | 0.000643326 |
| gene-Led00498-sp3 | 1.851583741 | 1.44195E-05 |
| gene-Led00518-sp3 | 0.804009042 | 0.001886901 |
| gene-Led00546-sp3 | -0.49095476 | 0.00842954 |
| gene-Led00547-sp3 | 0.662195798 | 0.004334512 |
| gene-Led00552-sp3 | -1.789534614 | 1.46269E-12 |
| gene-Led00553-sp3 | -0.816789836 | 0.001663816 |
| gene-Led00561-sp3 | -0.79618126 | 0.000662476 |
| gene-Led00566-sp3 | 1.003708608 | 3.90755E-05 |
| gene-Led00576-sp3 | 1.103143698 | 1.79782E-05 |
| gene-Led00588-sp3 | 2.160904831 | 1.46472E-22 |
| gene-Led00590-sp3 | 1.440001518 | 4.56987E-05 |
| gene-Led00619-sp3 | 1.134153234 | 3.59428E-07 |

| | | |
|---|---|---|
| gene-Led00647-sp3 | -2.084032588 | 1.95085E-20 |
| gene-Led00649-sp3 | -0.727146548 | 0.001158677 |
| gene-Led00674-sp3 | -0.58203015 | 0.001871654 |
| gene-Led00680-sp3 | 1.703287782 | 5.01126E-16 |
| gene-Led00683-sp3 | -2.190896476 | 6.09954E-05 |
| gene-Led00709-sp3 | 3.698918697 | 2.63552E-08 |
| gene-Led00718-sp3 | -0.704514063 | 0.000782845 |
| gene-Led00727-sp3 | 0.887330869 | 0.002913291 |
| gene-Led00734-sp3 | 3.61267099 | 4.3916E-06 |
| gene-Led00741-sp3 | 2.90566424 | 2.93506E-05 |
| gene-Led00742-sp3 | 0.711313884 | 0.005254305 |
| gene-Led00754-sp3 | 1.302716041 | 2.57833E-08 |
| gene-Led00777-sp3 | 0.779114679 | 1.72424E-05 |
| gene-Led00801-sp3 | 1.368241351 | 7.67347E-06 |
| gene-Led00803-sp3 | 0.466451691 | 0.00836403 |
| gene-Led00823-sp3 | -5.882022805 | 1.97287E-05 |
| gene-Led00848-sp3 | -0.928002297 | 0.001081272 |
| gene-Led00865-sp3 | -0.594207518 | 0.000382155 |
| gene-Led00874-sp3 | -0.660337366 | 0.003999704 |
| gene-Led00883-sp3 | 1.689699223 | 8.14861E-07 |
| gene-Led00902-sp3 | -2.796710554 | 1.21378E-42 |
| gene-Led00903-sp3 | -1.52424143 | 2.63819E-11 |
| gene-Led00903-sp3 | -1.549090484 | 7.02398E-07 |
| gene-Led00904-sp3 | -3.391054925 | 3.17283E-11 |
| gene-Led00905-sp3 | -3.281519208 | 1.39479E-50 |
| gene-Led00906-sp3 | -2.014416397 | 1.77216E-20 |
| gene-Led00907-sp3 | -3.884326819 | 9.74899E-30 |
| gene-Led00907-sp3 | -2.254661949 | 7.75573E-10 |
| gene-Led00908-sp3 | -1.315011368 | 1.50856E-09 |
| gene-Led00909-sp3 | -1.424355373 | 5.25946E-07 |
| gene-Led00910-sp3 | -2.164865842 | 3.8439E-34 |
| gene-Led00911-sp3 | -1.505521708 | 0.001224366 |
| gene-Led00912-sp3 | -3.681680696 | 1.78493E-08 |
| gene-Led00918-sp3 | 2.26237869 | 2.33048E-06 |
| gene-Led00926-sp3 | 2.459129287 | 9.85121E-08 |
| gene-Led00929-sp3 | -1.431837373 | 1.76685E-05 |
| gene-Led00934-sp3 | 0.70639732 | 0.003805375 |
| gene-Led00953-sp3 | 0.883803944 | 0.002351785 |
| gene-Led00958-sp3 | 0.706045191 | 0.004404376 |
| gene-Led00964-sp3 | 0.590531868 | 0.006962922 |
| gene-Led00965-sp3 | -0.487790343 | 0.009991135 |
| gene-Led00983-sp3 | 0.978142104 | 0.005768076 |
| gene-Led01009-sp3 | 0.807690905 | 0.001792685 |
| gene-Led01037-sp3 | 1.540036982 | 0.00120251 |

| | | |
|---|---|---|
| gene-Led01085-sp3 | 0.51651761 | 0.003073956 |
| gene-Led01098-sp3 | -0.909720314 | 0.00012739 |
| gene-Led01114-sp3 | 0.483696816 | 0.001337716 |
| gene-Led01116-sp3 | 1.244172639 | 9.15608E-08 |
| gene-Led01127-sp3 | -0.471713873 | 0.005867332 |
| gene-Led01128-sp3 | -0.736318619 | 5.5696E-06 |
| gene-Led01141-sp3 | -0.698763728 | 0.006844689 |
| gene-Led01142-sp3 | 0.780564648 | 0.000308583 |
| gene-Led01166-sp3 | 2.468407498 | 5.55362E-12 |
| gene-Led01172-sp3 | -0.670953136 | 0.006027351 |
| gene-Led01180-sp3 | -1.823317634 | 2.02353E-05 |
| gene-Led01180-sp3 | -1.537770875 | 4.25003E-05 |
| gene-Led01188-sp3 | 0.931882131 | 0.006057234 |
| gene-Led01196-sp3 | 1.854230563 | 1.10817E-05 |
| gene-Led01201-sp3 | -2.353524978 | 4.15209E-14 |
| gene-Led01207-sp3 | -0.56359016 | 0.002350304 |
| gene-Led01218-sp3 | -1.179773271 | 2.29747E-09 |
| gene-Led01221-sp3 | -0.697587695 | 2.71954E-05 |
| gene-Led01245-sp3 | -0.972796366 | 1.37591E-06 |
| gene-Led01248-sp3 | 1.03039838 | 0.002251794 |
| gene-Led01254-sp3 | 0.649971581 | 0.000412472 |
| gene-Led01265-sp3 | 3.204783988 | 0.000546408 |
| gene-Led01267-sp3 | -1.17118334 | 0.000589457 |
| gene-Led01274-sp3 | 0.633157351 | 0.000139593 |
| gene-Led01276-sp3 | 2.191635978 | 7.59458E-34 |
| gene-Led01279-sp3 | 0.593844269 | 0.004192933 |
| gene-Led01302-sp3 | 3.164139345 | 6.67081E-07 |
| gene-Led01305-sp3 | 0.518740707 | 0.006905094 |
| gene-Led01312-sp3 | 1.509506085 | 1.48109E-06 |
| gene-Led01325-sp3 | -4.838011107 | 1.23629E-65 |
| gene-Led01330-sp3 | -0.634112334 | 0.001278767 |
| gene-Led01338-sp3 | -1.073241965 | 1.02299E-07 |
| gene-Led01347-sp3 | 2.485376335 | 9.36212E-14 |
| gene-Led01354-sp3 | 0.991549154 | 0.006869015 |
| gene-Led01359-sp3 | 2.409658554 | 5.24921E-11 |
| gene-Led01379-sp3 | 1.220963231 | 0.000122668 |
| gene-Led01383-sp3 | -0.514830457 | 0.006369405 |
| gene-Led01400-sp3 | 0.464559546 | 0.009198308 |
| gene-Led01407-sp3 | -1.400482993 | 0.000102593 |
| gene-Led01431-sp3 | 1.090871164 | 9.87619E-05 |
| gene-Led01439-sp3 | 1.058092316 | 0.002066928 |
| gene-Led01443-sp3 | -1.175464654 | 0.001218148 |
| gene-Led01458-sp3 | 1.599987621 | 8.15737E-09 |
| gene-Led01472-sp3 | 1.230094078 | 2.42569E-07 |

| | | |
|---|---|---|
| gene-Led01474-sp3 | 0.919721509 | 0.003002733 |
| gene-Led01483-sp3 | -2.056082174 | 0.00037901 |
| gene-Led01492-sp3 | -1.367764623 | 3.07299E-05 |
| gene-Led01498-sp3 | 1.148761356 | 5.77318E-06 |
| gene-Led01536-sp3 | -2.252985311 | 2.92875E-26 |
| gene-Led01537-sp3 | 2.283737485 | 2.6864E-05 |
| gene-Led01538-sp3 | 4.927701713 | 1.3464E-79 |
| gene-Led01539-sp3 | 1.940358964 | 4.11021E-12 |
| gene-Led01543-sp3 | -0.547421475 | 0.006030843 |
| gene-Led01550-sp3 | 1.274132734 | 5.1584E-07 |
| gene-Led01557-sp3 | 3.73181655 | 1.97225E-05 |
| gene-Led01557-sp3 | 3.766812803 | 0.000243581 |
| gene-Led01567-sp3 | 2.734298119 | 3.76071E-24 |
| gene-Led01568-sp3 | 0.774271973 | 4.983E-05 |
| gene-Led01571-sp3 | -0.914248844 | 0.006783755 |
| gene-Led01573-sp3 | -0.640124547 | 2.09911E-05 |
| gene-Led01578-sp3 | -2.272079296 | 5.61155E-12 |
| gene-Led01608-sp3 | -0.66222948 | 2.63012E-05 |
| gene-Led01619-sp3 | -0.743374806 | 0.000117117 |
| gene-Led01630-sp3 | 3.182377301 | 4.2688E-43 |
| gene-Led01634-sp3 | -1.283728534 | 1.25304E-06 |
| gene-Led01659-sp3 | -1.983142425 | 8.99297E-06 |
| gene-Led01665-sp3 | -0.678227348 | 4.72488E-05 |
| gene-Led01667-sp3 | -0.757938068 | 0.000634751 |
| gene-Led01670-sp3 | 0.612032317 | 0.001444125 |
| gene-Led01673-sp3 | -1.378098853 | 5.22302E-08 |
| gene-Led01693-sp3 | 1.617623474 | 0.001370396 |
| gene-Led01718-sp3 | -2.40729298 | 2.94644E-17 |
| gene-Led01721-sp3 | -0.569288784 | 0.000169453 |
| gene-Led01763-sp3 | 1.958037565 | 1.79148E-06 |
| gene-Led01764-sp3 | 4.802537618 | 2.90531E-56 |
| gene-Led01765-sp3 | 6.387544245 | 2.21301E-40 |
| gene-Led01766-sp3 | 0.924927112 | 0.00777099 |
| gene-Led01767-sp3 | 1.099234336 | 6.31365E-07 |
| gene-Led01769-sp3 | -0.508130908 | 0.008433023 |
| gene-Led01793-sp3 | 0.770370408 | 0.005856614 |
| gene-Led01797-sp3 | 1.085544063 | 2.61794E-06 |
| gene-Led01806-sp3 | 1.463950531 | 4.89525E-08 |
| gene-Led01832-sp3 | 0.898881852 | 0.000894154 |
| gene-Led01859-sp3 | 2.573581942 | 7.06207E-11 |
| gene-Led01902-sp3 | -0.663025209 | 0.000342826 |
| gene-Led01916-sp3 | -1.069130228 | 5.16304E-09 |
| gene-Led01926-sp3 | -0.645943521 | 0.000951947 |
| gene-Led01935-sp3 | -0.783968614 | 7.31694E-05 |

| | | |
|---|---|---|
| gene-Led01940-sp3 | 0.809076141 | 0.000225404 |
| gene-Led01960-sp3 | -0.78992715 | 5.00298E-05 |
| gene-Led01973-sp3 | 0.878789305 | 0.00378887 |
| gene-Led01978-sp3 | -3.548779333 | 1.27813E-06 |
| gene-Led02016-sp3 | 2.119305397 | 4.09072E-15 |
| gene-Led02021-sp3 | 1.862868093 | 4.15891E-05 |
| gene-Led02047-sp3 | -0.800360735 | 2.3235E-05 |
| gene-Led02054-sp3 | 0.960974216 | 0.001799291 |
| gene-Led02063-sp3 | 0.615984437 | 0.002229426 |
| gene-Led02111-sp3 | 1.578178926 | 8.957E-09 |
| gene-Led02118-sp3 | -0.40715274 | 0.006010087 |
| gene-Led02123-sp3 | -0.699978223 | 0.000172722 |
| gene-Led02127-sp3 | -0.550614571 | 0.001428114 |
| gene-Led02135-sp3 | -0.865364397 | 0.000516078 |
| gene-Led02157-sp3 | 0.679613812 | 0.001340323 |
| gene-Led02158-sp3 | -0.439376012 | 0.004463994 |
| gene-Led02190-sp3 | 0.856717437 | 0.000141953 |
| gene-Led02200-sp3 | 1.524798475 | 3.15664E-06 |
| gene-Led02223-sp3 | -0.469282635 | 0.004586789 |
| gene-Led02238-sp3 | -0.919668467 | 7.21461E-06 |
| gene-Led02241-sp3 | 1.599102495 | 1.79816E-05 |
| gene-Led02251-sp3 | 2.090772018 | 1.49269E-05 |
| gene-Led02252-sp3 | 1.112252542 | 0.002689528 |
| gene-Led02273-sp3 | 1.420644464 | 3.65732E-12 |
| gene-Led02291-sp3 | 1.781949574 | 0.000387949 |
| gene-Led02325-sp3 | 0.82736047 | 2.3485E-05 |
| gene-Led02354-sp3 | -0.914448025 | 7.6949E-06 |
| gene-Led02368-sp3 | 0.894266244 | 0.000141548 |
| gene-Led02376-sp3 | -0.817668789 | 0.007984495 |
| gene-Led02377-sp3 | -1.402157175 | 7.56242E-05 |
| gene-Led02381-sp3 | 0.66105974 | 0.008425202 |
| gene-Led02397-sp3 | 0.967329532 | 0.001169744 |
| gene-Led02399-sp3 | 0.953133945 | 0.001485845 |
| gene-Led02415-sp3 | -0.522004111 | 0.009454702 |
| gene-Led02417-sp3 | 0.702032739 | 2.2974E-05 |
| gene-Led02440-sp3 | -1.994144095 | 1.83516E-07 |
| gene-Led02453-sp3 | 0.884010965 | 0.005582535 |
| gene-Led02458-sp3 | -0.811904174 | 0.006804898 |
| gene-Led02459-sp3 | -0.735152472 | 0.003467173 |
| gene-Led02461-sp3 | 1.050955636 | 0.003754669 |
| gene-Led02494-sp3 | 4.175194663 | 1.08153E-09 |
| gene-Led02500-sp3 | 4.237267902 | 1.09335E-06 |
| gene-Led02501-sp3 | 1.205946374 | 1.91092E-09 |
| gene-Led02516-sp3 | -1.166565536 | 0.001704395 |

| | | |
|---|---|---|
| gene-Led02558-sp3 | -0.923126637 | 0.002417277 |
| gene-Led02609-sp3 | -0.465729684 | 0.00906399 |
| gene-Led02620-sp3 | 1.591019107 | 0.001776882 |
| gene-Led02639-sp3 | -0.6777906 | 7.74449E-05 |
| gene-Led02666-sp3 | 0.619570322 | 0.001349945 |
| gene-Led02679-sp3 | 1.090634709 | 0.00611846 |
| gene-Led02690-sp3 | -0.710287702 | 0.006413959 |
| gene-Led02699-sp3 | 1.531122571 | 0.005375417 |
| gene-Led02726-sp3 | 1.15907342 | 8.15542E-06 |
| gene-Led02736-sp3 | 3.021300088 | 9.50476E-29 |
| gene-Led02745-sp3 | 1.208090889 | 0.000885465 |
| gene-Led02746-sp3 | 0.866186488 | 3.65449E-05 |
| gene-Led02747-sp3 | 0.731792607 | 0.009176666 |
| gene-Led02755-sp3 | -1.299859247 | 8.52363E-10 |
| gene-Led02757-sp3 | 2.19363868 | 4.12426E-11 |
| gene-Led02758-sp3 | 0.628649593 | 0.001928109 |
| gene-Led02776-sp3 | 0.908142288 | 3.61478E-06 |
| gene-Led02777-sp3 | -0.73376305 | 5.45666E-06 |
| gene-Led02804-sp3 | -0.530194268 | 0.000355711 |
| gene-Led02852-sp3 | -0.563789851 | 0.000567735 |
| gene-Led02859-sp3 | -0.812310695 | 4.78948E-07 |
| gene-Led02886-sp3 | -0.514288457 | 0.009274644 |
| gene-Led02900-sp3 | 0.66293503 | 0.000358052 |
| gene-Led02903-sp3 | 0.582008791 | 0.004908491 |
| gene-Led02910-sp3 | -1.717741391 | 3.45811E-10 |
| gene-Led02964-sp3 | -1.28375191 | 0.002510229 |
| gene-Led03018-sp3 | 1.25687249 | 2.60873E-10 |
| gene-Led03045-sp3 | 1.391380775 | 0.00179193 |
| gene-Led03054-sp3 | -0.792729551 | 5.78348E-07 |
| gene-Led03066-sp3 | 0.706022391 | 3.21781E-05 |
| gene-Led03067-sp3 | -0.646458638 | 0.005079285 |
| gene-Led03075-sp3 | 0.611307125 | 0.004217108 |
| gene-Led03155-sp3 | -0.691920633 | 0.00978313 |
| gene-Led03165-sp3 | 0.592364542 | 0.007454435 |
| gene-Led03168-sp3 | 1.299775966 | 1.40292E-08 |
| gene-Led03171-sp3 | 1.002912232 | 0.000885286 |
| gene-Led03172-sp3 | -2.167534313 | 8.97276E-05 |
| gene-Led03176-sp3 | -0.704137486 | 0.001561511 |
| gene-Led03200-sp3 | 1.795190258 | 4.19832E-07 |
| gene-Led03201-sp3 | 2.821452631 | 1.46998E-08 |
| gene-Led03203-sp3 | -1.141980323 | 1.5725E-07 |
| gene-Led03207-sp3 | -0.978196547 | 1.40324E-06 |
| gene-Led03223-sp3 | -0.628497475 | 0.007192412 |
| gene-Led03224-sp3 | 1.221958954 | 2.84628E-05 |

| | | |
|---|---|---|
| gene-Led03229-sp3 | 1.211328004 | 0.001700279 |
| gene-Led03234-sp3 | 0.544361247 | 0.006653595 |
| gene-Led03235-sp3 | 1.180884443 | 0.002918272 |
| gene-Led03250-sp3 | 2.145315055 | 2.71806E-10 |
| gene-Led03266-sp3 | -0.721675263 | 0.001337908 |
| gene-Led03270-sp3 | 1.104980404 | 0.001463176 |
| gene-Led03273-sp3 | 1.971038422 | 0.000841579 |
| gene-Led03287-sp3 | 2.782986473 | 4.89823E-22 |
| gene-Led03290-sp3 | -0.559442648 | 0.000100407 |
| gene-Led03291-sp3 | 0.586237523 | 0.003565417 |
| gene-Led03298-sp3 | 1.010103727 | 6.27998E-05 |
| gene-Led03302-sp3 | 0.922888577 | 1.05811E-05 |
| gene-Led03303-sp3 | 1.596020043 | 3.07704E-08 |
| gene-Led03323-sp3 | 0.731032294 | 0.000195576 |
| gene-Led03332-sp3 | -0.837001621 | 0.001552338 |
| gene-Led03426-sp3 | 1.291341255 | 2.42958E-07 |
| gene-Led03433-sp3 | 0.753668288 | 2.13815E-05 |
| gene-Led03434-sp3 | 3.868504161 | 2.46465E-27 |
| gene-Led03441-sp3 | 1.196240801 | 1.83627E-05 |
| gene-Led03446-sp3 | -0.670119967 | 4.8124E-05 |
| gene-Led03469-sp3 | 1.26096065 | 0.000877593 |
| gene-Led03474-sp3 | 0.639118613 | 0.006026058 |
| gene-Led03483-sp3 | -0.7003502 | 0.005850075 |
| gene-Led03484-sp3 | 2.123732995 | 4.61766E-08 |
| gene-Led03486-sp3 | 1.424445964 | 2.20964E-06 |
| gene-Led03487-sp3 | 1.96742038 | 0.000172264 |
| gene-Led03490-sp3 | -0.941441362 | 4.18753E-06 |
| gene-Led03534-sp3 | -1.061724815 | 0.004219259 |
| gene-Led03547-sp3 | 1.594562748 | 0.000714473 |
| gene-Led03557-sp3 | -1.41883355 | 1.23073E-05 |
| gene-Led03564-sp3 | 0.858035633 | 0.001346317 |
| gene-Led03570-sp3 | -1.586807249 | 8.56116E-08 |
| gene-Led03576-sp3 | 2.479066814 | 2.52675E-19 |
| gene-Led03580-sp3 | -0.439255786 | 0.009448821 |
| gene-Led03623-sp3 | 1.728762434 | 2.36328E-08 |
| gene-Led03628-sp3 | 1.025859297 | 6.25008E-05 |
| gene-Led03634-sp3 | -0.39378679 | 0.006946101 |
| gene-Led03665-sp3 | 1.392562463 | 0.000239657 |
| gene-Led03668-sp3 | 1.591694469 | 0.000495327 |
| gene-Led03676-sp3 | 2.346930645 | 0.002881162 |
| gene-Led03682-sp3 | -1.479943616 | 8.90829E-08 |
| gene-Led03702-sp3 | 2.01521207 | 3.19616E-28 |
| gene-Led03708-sp3 | 1.561254213 | 0.001627154 |
| gene-Led03712-sp3 | 1.014045204 | 2.29436E-06 |

| | | |
|---|---|---|
| gene-Led03736-sp3 | 2.476119415 | 1.57482E-12 |
| gene-Led03743-sp3 | -1.064585217 | 0.000194462 |
| gene-Led03748-sp3 | 2.773985078 | 1.96697E-05 |
| gene-Led03760-sp3 | -1.794758635 | 0.002458813 |
| gene-Led03786-sp3 | -1.31155262 | 0.000633193 |
| gene-Led03789-sp3 | -1.519045667 | 0.000823152 |
| gene-Led03810-sp3 | -1.085350388 | 0.001483443 |
| gene-Led03838-sp3 | 2.857665281 | 0.001065659 |
| gene-Led03839-sp3 | 2.081781982 | 2.942E-06 |
| gene-Led03840-sp3 | 1.13415171 | 0.001168243 |
| gene-Led03841-sp3 | -0.990961237 | 1.99178E-05 |
| gene-Led03850-sp3 | -0.592835119 | 0.000987123 |
| gene-Led03859-sp3 | 0.575533401 | 0.008731128 |
| gene-Led03886-sp3 | 1.636107868 | 1.71161E-13 |
| gene-Led03887-sp3 | 2.293763623 | 4.4965E-34 |
| gene-Led03888-sp3 | 1.908210761 | 1.75233E-20 |
| gene-Led03896-sp3 | 3.074104187 | 6.90711E-25 |
| gene-Led03898-sp3 | 0.503135354 | 0.002700546 |
| gene-Led03899-sp3 | 0.589030515 | 0.007038794 |
| gene-Led03907-sp3 | 3.143083968 | 6.42467E-17 |
| gene-Led03908-sp3 | 3.593250168 | 8.19892E-06 |
| gene-Led03922-sp3 | 1.738477084 | 2.77953E-05 |
| gene-Led03932-sp3 | -1.937257998 | 3.44707E-10 |
| gene-Led03934-sp3 | 0.772282989 | 3.76646E-05 |
| gene-Led03965-sp3 | 3.294730111 | 1.4781E-06 |
| gene-Led03978-sp3 | -0.620688853 | 0.000470533 |
| gene-Led03985-sp3 | -0.795154106 | 3.58692E-08 |
| gene-Led04043-sp3 | 1.208218915 | 0.00980994 |
| gene-Led04044-sp3 | 1.022377979 | 0.001732453 |
| gene-Led04058-sp3 | 1.192388399 | 1.07498E-05 |
| gene-Led04109-sp3 | -0.615123059 | 0.00261478 |
| gene-Led04110-sp3 | 1.606007403 | 0.004621768 |
| gene-Led04114-sp3 | 1.068247913 | 0.002095556 |
| gene-Led04117-sp3 | 1.780051034 | 0.000956165 |
| gene-Led04121-sp3 | -1.161088103 | 2.44827E-08 |
| gene-Led04128-sp3 | 1.676244747 | 6.20489E-10 |
| gene-Led04131-sp3 | -1.299963768 | 0.001879511 |
| gene-Led04147-sp3 | 1.786975878 | 8.01648E-07 |
| gene-Led04157-sp3 | 2.609414814 | 1.63261E-10 |
| gene-Led04171-sp3 | -0.69412909 | 0.006156171 |
| gene-Led04193-sp3 | 0.795109113 | 0.002063982 |
| gene-Led04203-sp3 | 0.84569092 | 2.02613E-05 |
| gene-Led04228-sp3 | 1.503555689 | 3.19396E-06 |
| gene-Led04261-sp3 | -1.059953932 | 0.000417437 |

| | | |
|---|---|---|
| gene-Led04270-sp3 | 1.58153208 | 0.000688079 |
| gene-Led04270-sp3 | 1.452916343 | 0.002376406 |
| gene-Led04279-sp3 | -1.67197371 | 3.25779E-09 |
| gene-Led04282-sp3 | 2.202068833 | 6.24407E-09 |
| gene-Led04290-sp3 | 1.675330939 | 0.002607013 |
| gene-Led04295-sp3 | 0.580297482 | 0.009542693 |
| gene-Led04303-sp3 | -1.430079032 | 3.55528E-17 |
| gene-Led04313-sp3 | -0.588995897 | 0.001868755 |
| gene-Led04336-sp3 | -2.114661161 | 2.92287E-05 |
| gene-Led04345-sp3 | -2.004682162 | 0.00074514 |
| gene-Led04358-sp3 | -0.911072451 | 0.00204359 |
| gene-Led04372-sp3 | -0.546138369 | 0.000478732 |
| gene-Led04391-sp3 | 1.124616251 | 9.86048E-07 |
| gene-Led04402-sp3 | 0.756513154 | 3.72168E-05 |
| gene-Led04417-sp3 | 0.53390111 | 0.007496995 |
| gene-Led04429-sp3 | -0.747820891 | 8.34433E-05 |
| gene-Led04463-sp3 | 0.682737791 | 0.003264788 |
| gene-Led04471-sp3 | -0.586088672 | 0.000619325 |
| gene-Led04492-sp3 | -0.645658064 | 0.000672636 |
| gene-Led04494-sp3 | 0.678958117 | 0.009006729 |
| gene-Led04499-sp3 | 3.669429485 | 8.71098E-56 |
| gene-Led04512-sp3 | 1.341415046 | 0.000211035 |
| gene-Led04519-sp3 | -1.090643139 | 0.00022038 |
| gene-Led04540-sp3 | -0.521976999 | 0.007008425 |
| gene-Led04554-sp3 | -0.466252087 | 0.0088078 |
| gene-Led04558-sp3 | 1.239788429 | 7.07356E-06 |
| gene-Led04562-sp3 | -1.362418901 | 2.96913E-06 |
| gene-Led04568-sp3 | -1.682761302 | 2.43052E-08 |
| gene-Led04604-sp3 | 2.124390033 | 1.35718E-05 |
| gene-Led04617-sp3 | -1.262065544 | 0.001148926 |
| gene-Led04641-sp3 | 1.379290444 | 0.001514288 |
| gene-Led04642-sp3 | -1.195684845 | 4.75971E-06 |
| gene-Led04658-sp3 | -0.620564581 | 0.009244895 |
| gene-Led04663-sp3 | 1.902707443 | 2.50381E-10 |
| gene-Led04681-sp3 | 1.324313555 | 0.001385679 |
| gene-Led04683-sp3 | -4.325295599 | 9.79153E-11 |
| gene-Led04692-sp3 | 0.72471198 | 0.009065365 |
| gene-Led04763-sp3 | 1.863808007 | 3.72508E-12 |
| gene-Led04771-sp3 | 1.459337646 | 1.9738E-06 |
| gene-Led04796-sp3 | -0.916478465 | 0.007867079 |
| gene-Led04806-sp3 | -1.312706479 | 0.001208405 |
| gene-Led04816-sp3 | -1.27903459 | 2.28526E-05 |
| gene-Led04817-sp3 | -0.753792601 | 1.73536E-05 |
| gene-Led04817-sp3 | -0.824831063 | 9.74753E-05 |

| | | |
|---|---|---|
| gene-Led04838-sp3 | 1.372229883 | 0.00424542 |
| gene-Led04942-sp3 | -1.213417618 | 1.28077E-07 |
| gene-Led04945-sp3 | -1.745918356 | 1.66877E-25 |
| gene-Led04946-sp3 | -1.306851322 | 9.86821E-05 |
| gene-Led04949-sp3 | -0.410683016 | 0.007870615 |
| gene-Led04950-sp3 | -0.626772705 | 0.000316837 |
| gene-Led04955-sp3 | 1.079399458 | 2.74017E-07 |
| gene-Led04958-sp3 | 3.031789407 | 2.32853E-40 |
| gene-Led04959-sp3 | 1.296993886 | 0.001327923 |
| gene-Led04960-sp3 | 1.628050899 | 1.25746E-07 |
| gene-Led04968-sp3 | 2.373360675 | 0.001613967 |
| gene-Led04985-sp3 | 1.148743566 | 0.002090802 |
| gene-Led04987-sp3 | -0.64046532 | 0.004924704 |
| gene-Led05009-sp3 | 0.724194857 | 0.004213292 |
| gene-Led05017-sp3 | 2.950336962 | 6.463E-13 |
| gene-Led05022-sp3 | -2.943187723 | 3.75977E-10 |
| gene-Led05031-sp3 | -2.855136426 | 5.07846E-30 |
| gene-Led05034-sp3 | -1.803055188 | 2.05626E-20 |
| gene-Led05039-sp3 | -1.430200759 | 0.000151647 |
| gene-Led05048-sp3 | 0.75611068 | 0.005727769 |
| gene-Led05049-sp3 | 0.93435746 | 0.000498929 |
| gene-Led05056-sp3 | 0.715191655 | 0.005711481 |
| gene-Led05062-sp3 | -2.885390397 | 2.87437E-15 |
| gene-Led05063-sp3 | -3.525810556 | 1.33611E-21 |
| gene-Led05068-sp3 | 4.499562308 | 1.09418E-25 |
| gene-Led05087-sp3 | 2.142987266 | 2.91176E-05 |
| gene-Led05094-sp3 | 0.932724376 | 0.001218345 |
| gene-Led05095-sp3 | -0.86013312 | 0.00030357 |
| gene-Led05099-sp3 | 0.577712022 | 0.005196166 |
| gene-Led05105-sp3 | -1.378450294 | 4.70067E-05 |
| gene-Led05123-sp3 | 3.250692696 | 6.60102E-13 |
| gene-Led05126-sp3 | -1.546314488 | 0.000395297 |
| gene-Led05130-sp3 | -1.827859596 | 8.6726E-13 |
| gene-Led05132-sp3 | -0.762421694 | 0.004680298 |
| gene-Led05133-sp3 | -5.161890613 | 6.70132E-20 |
| gene-Led05139-sp3 | 1.415275042 | 0.000860249 |
| gene-Led05144-sp3 | 0.749552065 | 4.5904E-06 |
| gene-Led05145-sp3 | 0.862812905 | 0.000244913 |
| gene-Led05164-sp3 | 0.899648528 | 0.007463948 |
| gene-Led05169-sp3 | 1.745175311 | 4.12216E-05 |
| gene-Led05176-sp3 | 1.556162454 | 0.000397417 |
| gene-Led05203-sp3 | -0.92065833 | 2.91251E-07 |
| gene-Led05207-sp3 | -2.516985683 | 2.35294E-08 |
| gene-Led05219-sp3 | 1.04949866 | 5.31731E-05 |

| | | |
|---|---|---|
| gene-Led05230-sp3 | -0.52567749 | 0.001827475 |
| gene-Led05235-sp3 | 0.713607463 | 0.005309818 |
| gene-Led05285-sp3 | 1.557139955 | 0.000147761 |
| gene-Led05296-sp3 | -0.563205093 | 0.002107108 |
| gene-Led05329-sp3 | -0.509691064 | 0.005865355 |
| gene-Led05332-sp3 | 1.742726427 | 9.07258E-11 |
| gene-Led05340-sp3 | 1.079224433 | 2.19643E-08 |
| gene-Led05353-sp3 | -0.578421093 | 0.005292729 |
| gene-Led05408-sp3 | -0.42216822 | 0.009987205 |
| gene-Led05458-sp3 | 0.70313638 | 0.00013554 |
| gene-Led05491-sp3 | -0.482883965 | 0.008710342 |
| gene-Led05492-sp3 | 1.561007258 | 0.000613994 |
| gene-Led05570-sp3 | 4.238525651 | 2.97191E-15 |
| gene-Led05578-sp3 | 0.935725982 | 0.003366719 |
| gene-Led05591-sp3 | 0.623306665 | 0.002205107 |
| gene-Led05597-sp3 | -0.465682301 | 0.007805265 |
| gene-Led05631-sp3 | 1.051439402 | 0.001952282 |
| gene-Led05658-sp3 | -1.384132235 | 9.81366E-09 |
| gene-Led05693-sp3 | -1.49720992 | 3.01713E-09 |
| gene-Led05698-sp3 | -1.553429269 | 0.000565712 |
| gene-Led05717-sp3 | 1.065587929 | 0.006813444 |
| gene-Led05722-sp3 | 0.640705029 | 0.000858815 |
| gene-Led05728-sp3 | 1.624213336 | 6.75134E-12 |
| gene-Led05740-sp3 | -0.98603305 | 7.94012E-05 |
| gene-Led05743-sp3 | 1.347148181 | 3.83841E-05 |
| gene-Led05768-sp3 | -0.709583148 | 4.05281E-05 |
| gene-Led05770-sp3 | -0.865263325 | 0.001923155 |
| gene-Led05777-sp3 | -0.671265301 | 0.005586015 |
| gene-Led05808-sp3 | -1.420053386 | 1.61579E-11 |
| gene-Led05862-sp3 | -0.860284613 | 4.14778E-05 |
| gene-Led05864-sp3 | 0.549836902 | 0.000117229 |
| gene-Led05881-sp3 | 0.688363627 | 0.002581994 |
| gene-Led05883-sp3 | -0.676895387 | 0.001325675 |
| gene-Led05902-sp3 | 1.549923517 | 1.00535E-05 |
| gene-Led05919-sp3 | 0.679337075 | 0.006687292 |
| gene-Led05934-sp3 | 1.60709047 | 1.55752E-07 |
| gene-Led05959-sp3 | 0.884335344 | 0.000314868 |
| gene-Led05983-sp3 | 2.324911101 | 0.003188244 |
| gene-Led05984-sp3 | 2.160019276 | 0.004428618 |
| gene-Led05986-sp3 | 1.829800841 | 4.13535E-10 |
| gene-Led06007-sp3 | 1.08814987 | 0.001086051 |
| gene-Led06015-sp3 | 1.884871003 | 0.002481055 |
| gene-Led06017-sp3 | 1.363726545 | 9.94578E-06 |
| gene-Led06031-sp3 | -0.83837205 | 0.00010268 |

| | | |
|---|---|---|
| gene-Led06051-sp3 | 0.676593315 | 0.003522003 |
| gene-Led06055-sp3 | 1.4954956 | 4.04442E-06 |
| gene-Led06056-sp3 | -0.527422035 | 0.001413773 |
| gene-Led06059-sp3 | -2.09576403 | 6.69241E-19 |
| gene-Led06060-sp3 | -0.873297476 | 1.41765E-06 |
| gene-Led06075-sp3 | 2.130236797 | 3.94833E-06 |
| gene-Led06118-sp3 | 0.958741336 | 0.000586837 |
| gene-Led06124-sp3 | -1.126209467 | 7.1833E-08 |
| gene-Led06138-sp3 | -0.779580482 | 4.52911E-06 |
| gene-Led06162-sp3 | -3.884078346 | 1.68594E-09 |
| gene-Led06164-sp3 | -1.390272543 | 1.43213E-06 |
| gene-Led06165-sp3 | 1.301296983 | 1.23629E-10 |
| gene-Led06217-sp3 | 2.343191615 | 0.000391944 |
| gene-Led06227-sp3 | 1.296577132 | 0.000517786 |
| gene-Led06250-sp3 | 0.730966641 | 0.004563433 |
| gene-Led06253-sp3 | 1.119225455 | 4.36127E-05 |
| gene-Led06255-sp3 | 1.939896605 | 3.1833E-05 |
| gene-Led06265-sp3 | -1.93050662 | 1.86641E-10 |
| gene-Led06278-sp3 | -0.590263378 | 0.004462997 |
| gene-Led06279-sp3 | -1.368164676 | 1.24685E-06 |
| gene-Led06328-sp3 | -0.578333821 | 0.004365174 |
| gene-Led06338-sp3 | 2.458558883 | 3.5085E-33 |
| gene-Led06347-sp3 | 2.50175445 | 5.35954E-25 |
| gene-Led06349-sp3 | 0.860690794 | 0.007684596 |
| gene-Led06367-sp3 | -3.427848514 | 4.24632E-08 |
| gene-Led06391-sp3 | -5.4110145 | 9.50668E-60 |
| gene-Led06391-sp3 | -2.773971812 | 0.003202027 |
| gene-Led06410-sp3 | -0.518074913 | 0.009364206 |
| gene-Led06460-sp3 | -0.699072833 | 0.000619923 |
| gene-Led06464-sp3 | 0.826968641 | 0.001228376 |
| gene-Led06483-sp3 | -1.300604475 | 3.59864E-11 |
| gene-Led06499-sp3 | 1.387230031 | 0.000763977 |
| gene-Led06514-sp3 | -0.859634784 | 0.003347881 |
| gene-Led06524-sp3 | 2.093934954 | 1.92355E-20 |
| gene-Led06530-sp3 | 3.184755362 | 1.28387E-16 |
| gene-Led06540-sp3 | -0.504663912 | 0.007829486 |
| gene-Led06546-sp3 | 5.66082246 | 5.92273E-62 |
| gene-Led06565-sp3 | -0.793543837 | 0.001474509 |
| gene-Led06582-sp3 | -1.208514783 | 4.83107E-08 |
| gene-Led06598-sp3 | 0.934646167 | 1.54019E-05 |
| gene-Led06601-sp3 | -0.973782247 | 1.07229E-07 |
| gene-Led06603-sp3 | -1.408884877 | 7.52282E-07 |
| gene-Led06604-sp3 | -0.710461305 | 0.000175705 |
| gene-Led06642-sp3 | 2.255455441 | 1.46547E-09 |

| | | |
|---|---|---|
| gene-Led06668-sp3 | 1.375959839 | 0.001492787 |
| gene-Led06732-sp3 | 2.453963877 | 4.98775E-05 |
| gene-Led06772-sp3 | 2.106624742 | 9.02134E-09 |
| gene-Led06782-sp3 | 1.646457309 | 1.4345E-06 |
| gene-Led06801-sp3 | 1.539537839 | 0.002217044 |
| gene-Led06808-sp3 | 0.997709332 | 9.43791E-06 |
| gene-Led06827-sp3 | 2.559156392 | 1.25628E-05 |
| gene-Led06842-sp3 | 0.657646009 | 0.001954149 |
| gene-Led06846-sp3 | 0.57523407 | 0.007829274 |
| gene-Led06848-sp3 | 3.418438205 | 4.45433E-10 |
| gene-Led06850-sp3 | 4.571199774 | 7.67491E-14 |
| gene-Led06888-sp3 | 5.55941702 | 1.42335E-56 |
| gene-Led06929-sp3 | -0.583583234 | 0.002106102 |
| gene-Led06931-sp3 | 1.17852883 | 4.75323E-05 |
| gene-Led06933-sp3 | 0.736916703 | 0.005963003 |
| gene-Led06946-sp3 | -0.761855275 | 0.001067992 |
| gene-Led06949-sp3 | 0.592765063 | 0.000389139 |
| gene-Led06957-sp3 | 2.397203379 | 2.06242E-27 |
| gene-Led06993-sp3 | 0.928112371 | 0.007808909 |
| gene-Led06998-sp3 | -1.013877176 | 0.000120086 |
| gene-Led07002-sp3 | -1.009840129 | 1.05788E-05 |
| gene-Led07019-sp3 | 1.295783771 | 1.9422E-11 |
| gene-Led07029-sp3 | 1.326594347 | 0.005021919 |
| gene-Led07041-sp3 | -1.322230313 | 3.24176E-06 |
| gene-Led07043-sp3 | 2.581264153 | 1.36438E-07 |
| gene-Led07089-sp3 | 0.611796009 | 0.003205154 |
| gene-Led07092-sp3 | 2.083222583 | 3.14066E-07 |
| gene-Led07102-sp3 | -1.433297668 | 1.0352E-06 |
| gene-Led07104-sp3 | -0.624837613 | 0.001414951 |
| gene-Led07136-sp3 | -1.244025508 | 0.0003523 |
| gene-Led07148-sp3 | 0.943045706 | 0.001920019 |
| gene-Led07155-sp3 | -0.751270639 | 0.003927893 |
| gene-Led07180-sp3 | 2.447381288 | 6.16018E-11 |
| gene-Led07186-sp3 | -1.568180189 | 6.11193E-16 |
| gene-Led07202-sp3 | -0.728077798 | 0.000538942 |
| gene-Led07204-sp3 | 2.653221631 | 7.56216E-14 |
| gene-Led07205-sp3 | 0.9276758 | 0.002488934 |
| gene-Led07224-sp3 | 0.934445964 | 6.88787E-05 |
| gene-Led07227-sp3 | 1.260528969 | 7.31379E-08 |
| gene-Led07275-sp3 | 1.104004957 | 3.26055E-11 |
| gene-Led07300-sp3 | 1.245174901 | 2.5515E-09 |
| gene-Led07305-sp3 | -0.641378839 | 0.003575682 |
| gene-Led07321-sp3 | -0.444554893 | 0.00363381 |
| gene-Led07345-sp3 | 3.494695777 | 6.91622E-28 |

| | | |
|---|---|---|
| gene-Led07360-sp3 | 2.865840681 | 2.85841E-06 |
| gene-Led07388-sp3 | 0.580971583 | 0.008448087 |
| gene-Led07397-sp3 | 0.898921753 | 0.000227921 |
| gene-Led07404-sp3 | -2.214432501 | 1.80613E-07 |
| gene-Led07486-sp3 | -0.463072484 | 0.003832229 |
| gene-Led07492-sp3 | -0.54062742 | 0.006356027 |
| gene-Led07495-sp3 | -0.46867398 | 0.004371104 |
| gene-Led07503-sp3 | -0.744529889 | 0.000350212 |
| gene-Led07517-sp3 | -1.25158811 | 5.40567E-10 |
| gene-Led07525-sp3 | -1.482492758 | 8.87906E-15 |
| gene-Led07526-sp3 | 1.634423892 | 6.27418E-05 |
| gene-Led07526-sp3 | 1.199734664 | 0.003063471 |
| gene-Led07592-sp3 | 3.492240754 | 6.31336E-37 |
| gene-Led07595-sp3 | -3.976576827 | 2.08726E-61 |
| gene-Led07601-sp3 | -0.684180934 | 0.000869983 |
| gene-Led07604-sp3 | -0.736665346 | 3.12483E-05 |
| gene-Led07615-sp3 | 1.289349096 | 0.002113651 |
| gene-Led07626-sp3 | 1.867452853 | 3.80867E-15 |
| gene-Led07629-sp3 | 0.821580654 | 0.000269422 |
| gene-Led07637-sp3 | 0.562987597 | 0.001963062 |
| gene-Led07646-sp3 | -0.579320972 | 0.001172493 |
| gene-Led07647-sp3 | -0.542997806 | 0.007718723 |
| gene-Led07684-sp3 | -0.603745369 | 0.000221933 |
| gene-Led07685-sp3 | -1.415418143 | 1.87318E-07 |
| gene-Led07697-sp3 | 3.574130319 | 4.65955E-11 |
| gene-Led07738-sp3 | 0.94917231 | 1.13221E-06 |
| gene-Led07742-sp3 | 1.392097242 | 0.000626575 |
| gene-Led07744-sp3 | -1.648513841 | 0.000626582 |
| gene-Led07758-sp3 | 2.408498708 | 2.92744E-05 |
| gene-Led07788-sp3 | 0.940453751 | 4.06604E-06 |
| gene-Led07803-sp3 | 1.571115275 | 1.38415E-06 |
| gene-Led07810-sp3 | -0.696494917 | 0.000350841 |
| gene-Led07827-sp3 | -0.604135779 | 0.000874352 |
| gene-Led07845-sp3 | 1.67118207 | 2.09083E-11 |
| gene-Led07853-sp3 | -0.829611585 | 0.00019853 |
| gene-Led07856-sp3 | 1.063294108 | 0.001108253 |
| gene-Led07898-sp3 | 0.949576726 | 0.001776664 |
| gene-Led07904-sp3 | -0.671183851 | 4.82092E-05 |
| gene-Led07908-sp3 | 1.153615538 | 5.39705E-05 |
| gene-Led07914-sp3 | 0.949082367 | 0.000356815 |
| gene-Led07916-sp3 | 0.647022063 | 0.000363623 |
| gene-Led07917-sp3 | 1.028329944 | 0.000589696 |
| gene-Led07963-sp3 | 0.66983508 | 0.003090019 |
| gene-Led07979-sp3 | 0.740084121 | 6.24269E-05 |

| | | |
|---|---|---|
| gene-Led07993-sp3 | -0.81513435 | 0.001373578 |
| gene-Led08008-sp3 | -0.815860173 | 0.001290939 |
| gene-Led08017-sp3 | -0.560668092 | 0.001386518 |
| gene-Led08052-sp3 | -0.630892241 | 0.001783831 |
| gene-Led08072-sp3 | -1.364711254 | 0.000230251 |
| gene-Led08110-sp3 | 0.571369804 | 0.00373508 |
| gene-Led08161-sp3 | 0.656227408 | 0.009552113 |
| gene-Led08165-sp3 | 2.273819436 | 0.000175793 |
| gene-Led08168-sp3 | 2.387671234 | 5.37336E-08 |
| gene-Led08177-sp3 | 1.107825746 | 0.006997222 |
| gene-Led08181-sp3 | 1.629841801 | 7.1264E-05 |
| gene-Led08184-sp3 | 2.395592702 | 2.94889E-24 |
| gene-Led08187-sp3 | 4.355372641 | 3.27426E-31 |
| gene-Led08195-sp3 | 1.987203996 | 0.00164171 |
| gene-Led08200-sp3 | 1.043990001 | 7.4918E-05 |
| gene-Led08203-sp3 | -0.60015764 | 0.000401364 |
| gene-Led08204-sp3 | -0.659077086 | 0.000241298 |
| gene-Led08217-sp3 | -2.35823162 | 3.03791E-16 |
| gene-Led08218-sp3 | -0.76077714 | 0.003528576 |
| gene-Led08238-sp3 | 1.722859482 | 5.71868E-07 |
| gene-Led08252-sp3 | 1.51804875 | 0.000513803 |
| gene-Led08334-sp3 | -0.663091673 | 0.00111811 |
| gene-Led08399-sp3 | 0.837345168 | 0.00075504 |
| gene-Led08418-sp3 | -0.542786648 | 0.008958717 |
| gene-Led08422-sp3 | -0.614136226 | 0.008388944 |
| gene-Led08432-sp3 | 0.988402125 | 4.12001E-06 |
| gene-Led08448-sp3 | 2.277302402 | 6.3521E-18 |
| gene-Led08460-sp3 | -1.456412932 | 0.001156605 |
| gene-Led08464-sp3 | -1.02250629 | 0.004579178 |
| gene-Led08489-sp3 | 0.748899921 | 0.001224066 |
| gene-Led08516-sp3 | 1.307867889 | 2.17204E-10 |
| gene-Led08562-sp3 | 0.91969013 | 0.003917089 |
| gene-Led08569-sp3 | -0.999901333 | 0.000323684 |
| gene-Led08597-sp3 | -0.652265333 | 0.001842152 |
| gene-Led08608-sp3 | -1.907695911 | 0.002327857 |
| gene-Led08610-sp3 | 1.537783485 | 5.39353E-07 |
| gene-Led08615-sp3 | -0.394485829 | 0.009240239 |
| gene-Led08622-sp3 | -3.517094449 | 3.84141E-33 |
| gene-Led08624-sp3 | -5.441844495 | 1.63254E-15 |
| gene-Led08625-sp3 | -2.243311588 | 7.22257E-11 |
| gene-Led08636-sp3 | -0.77764238 | 0.002929496 |
| gene-Led08645-sp3 | -0.902029523 | 0.003971875 |
| gene-Led08653-sp3 | 0.841758506 | 0.003336651 |
| gene-Led08657-sp3 | 1.93309745 | 0.00197773 |

| | | |
|---|---|---|
| gene-Led08658-sp3 | 2.004482493 | 3.63316E-07 |
| gene-Led08658-sp3 | 1.79901145 | 0.002436622 |
| gene-Led08659-sp3 | 1.842540327 | 6.54508E-09 |
| gene-Led08681-sp3 | 0.847977764 | 0.004690931 |
| gene-Led08682-sp3 | 1.584239288 | 7.09324E-07 |
| gene-Led08706-sp3 | -1.954924933 | 1.57543E-09 |
| gene-Led08715-sp3 | -0.741404442 | 0.00112037 |
| gene-Led08727-sp3 | -0.655377662 | 0.006946148 |
| gene-Led08734-sp3 | -2.222116552 | 2.49932E-07 |
| gene-Led08744-sp3 | 0.980299802 | 0.003688888 |
| gene-Led08745-sp3 | -0.806103216 | 2.12231E-07 |
| gene-Led08750-sp3 | 1.092020908 | 0.000863707 |
| gene-Led08827-sp3 | -1.909657621 | 5.66755E-27 |
| gene-Led08829-sp3 | -1.435193876 | 1.81194E-09 |
| gene-Led08836-sp3 | 0.963867524 | 0.003697555 |
| gene-Led08838-sp3 | -0.897940544 | 1.84466E-06 |
| gene-Led08870-sp3 | 1.298817583 | 0.000437429 |
| gene-Led08877-sp3 | -1.150754606 | 5.27872E-10 |
| gene-Led08906-sp3 | 0.586909868 | 0.005379224 |
| gene-Led08908-sp3 | -0.557736734 | 0.005776293 |
| gene-Led08922-sp3 | -0.484030154 | 0.004397215 |
| gene-Led08924-sp3 | 1.828350727 | 2.14558E-08 |
| gene-Led08999-sp3 | -0.959540297 | 2.26542E-06 |
| gene-Led09023-sp3 | -0.624022807 | 7.78098E-05 |
| gene-Led09059-sp3 | -0.71701139 | 0.000779547 |
| gene-Led09060-sp3 | -0.908334963 | 0.006216908 |
| gene-Led09068-sp3 | 0.780754375 | 0.001643894 |
| gene-Led09072-sp3 | -0.619449052 | 0.000229501 |
| gene-Led09101-sp3 | -0.624145587 | 0.004550684 |
| gene-Led09126-sp3 | -0.872794747 | 4.14386E-06 |
| gene-Led09169-sp3 | -0.609594908 | 0.000123961 |
| gene-Led09171-sp3 | -0.534131779 | 0.004050505 |
| gene-Led09172-sp3 | -0.537249551 | 0.006870105 |
| gene-Led09175-sp3 | 1.325594459 | 2.84685E-08 |
| gene-Led09177-sp3 | 2.759227483 | 4.88207E-06 |
| gene-Led09201-sp3 | 1.13676146 | 4.05981E-06 |
| gene-Led09210-sp3 | 1.689427245 | 0.000340224 |
| gene-Led09233-sp3 | -0.66052839 | 0.001722301 |
| gene-Led09234-sp3 | 0.815674858 | 0.002691242 |
| gene-Led09259-sp3 | 0.788416769 | 0.006950857 |
| gene-Led09278-sp3 | -0.505388658 | 0.008457768 |
| gene-Led09320-sp3 | 1.762172357 | 0.008722943 |
| gene-Led09321-sp3 | 1.602068268 | 5.49021E-10 |
| gene-Led09374-sp3 | 1.912566199 | 0.002233024 |

| | | |
|---|---|---|
| gene-Led09378-sp3 | 2.705715513 | 3.02535E-05 |
| gene-Led09380-sp3 | -0.660830806 | 0.004511189 |
| gene-Led09386-sp3 | -1.435024723 | 0.004713274 |
| gene-Led09418-sp3 | 2.564373366 | 4.73145E-31 |
| gene-Led09424-sp3 | 2.135009213 | 3.00002E-16 |
| gene-Led09439-sp3 | -0.64264001 | 6.21279E-05 |
| gene-Led09442-sp3 | 0.583080291 | 0.003214366 |
| gene-Led09450-sp3 | -1.721171741 | 6.71106E-14 |
| gene-Led09488-sp3 | 0.779322027 | 0.002429388 |
| gene-Led09499-sp3 | 0.715622885 | 0.004777377 |
| gene-Led09503-sp3 | 1.084292279 | 0.001173354 |
| gene-Led09504-sp3 | -0.99561821 | 9.42931E-05 |
| gene-Led09505-sp3 | -0.664039211 | 0.003815058 |
| gene-Led09517-sp3 | -0.982741972 | 3.75971E-09 |
| gene-Led09543-sp3 | 0.820675393 | 0.004709888 |
| gene-Led09557-sp3 | -1.500963314 | 5.16571E-06 |
| gene-Led09573-sp3 | 0.621796917 | 0.001054973 |
| gene-Led09598-sp3 | -0.638477315 | 0.00378525 |
| gene-Led09619-sp3 | 2.055105239 | 9.38115E-05 |
| gene-Led09623-sp3 | 1.222544359 | 0.007307617 |
| gene-Led09663-sp3 | 1.200500582 | 0.000335815 |
| gene-Led09680-sp3 | 0.599892479 | 0.00939505 |
| gene-Led09696-sp3 | -1.235668275 | 5.76318E-13 |
| gene-Led09702-sp3 | 1.238210754 | 1.07887E-06 |
| gene-Led09711-sp3 | 1.14306781 | 0.000483519 |
| gene-Led09712-sp3 | 1.850671448 | 7.28832E-06 |
| gene-Led09714-sp3 | 1.127610451 | 0.000408751 |
| gene-Led09731-sp3 | -0.54442307 | 0.001694028 |
| gene-Led09769-sp3 | 1.075679992 | 0.006560984 |
| gene-Led09806-sp3 | 2.979651199 | 0.000628379 |
| gene-Led09808-sp3 | 1.103997393 | 0.000315938 |
| gene-Led09812-sp3 | -0.90768372 | 0.003677944 |
| gene-Led09836-sp3 | 1.054827268 | 0.000856224 |
| gene-Led09839-sp3 | -0.55568313 | 0.000475408 |
| gene-Led09846-sp3 | -0.818633782 | 1.59123E-06 |
| gene-Led09895-sp3 | -0.740997964 | 0.001823826 |
| gene-Led09984-sp3 | -0.740239427 | 0.000164141 |
| gene-Led09989-sp3 | 0.693245089 | 0.00595943 |
| gene-Led10005-sp3 | 1.473238559 | 0.001860907 |
| gene-Led10006-sp3 | 2.161013982 | 1.82842E-08 |
| gene-Led10034-sp3 | -0.522401045 | 0.00662565 |
| gene-Led10040-sp3 | -0.502365407 | 0.002698797 |
| gene-Led10050-sp3 | 1.209165375 | 5.65767E-07 |
| gene-Led10065-sp3 | 0.678251257 | 0.005121503 |

| | | |
|---|---|---|
| gene-Led10108-sp3 | 3.700598959 | 2.39187E-06 |
| gene-Led10111-sp3 | 0.632235653 | 0.004696889 |
| gene-Led10121-sp3 | 0.881716539 | 0.00195228 |
| gene-Led10163-sp3 | 1.542706748 | 4.6195E-05 |
| gene-Led10176-sp3 | 0.779414847 | 0.00016738 |
| gene-Led10184-sp3 | 2.005597819 | 5.1888E-10 |
| gene-Led10210-sp3 | 1.449863263 | 5.94211E-07 |
| gene-Led10217-sp3 | -0.749697743 | 0.007465637 |
| gene-Led10225-sp3 | 1.107036715 | 1.17967E-06 |
| gene-Led10239-sp3 | -0.944258187 | 2.78613E-06 |
| gene-Led10246-sp3 | 1.384430806 | 2.12736E-15 |
| gene-Led10262-sp3 | -0.382715066 | 0.003562503 |
| gene-Led10312-sp3 | 2.115878201 | 6.06552E-08 |
| gene-Led10316-sp3 | 2.335195994 | 1.12441E-15 |
| gene-Led10321-sp3 | -1.92395671 | 8.99548E-06 |
| gene-Led10322-sp3 | 2.636242024 | 7.40555E-06 |
| gene-Led10323-sp3 | -1.4152756 | 0.000107172 |
| gene-Led10329-sp3 | 0.923116721 | 0.001414392 |
| gene-Led10340-sp3 | 1.313542633 | 0.000136438 |
| gene-Led10354-sp3 | 1.210000665 | 0.000168309 |
| gene-Led10375-sp3 | -0.621572609 | 0.000695739 |
| gene-Led10477-sp3 | 1.881649481 | 5.01388E-06 |
| gene-Led10479-sp3 | 4.021739235 | 2.764E-18 |
| gene-Led10483-sp3 | -2.327037328 | 1.19454E-08 |
| gene-Led10484-sp3 | -1.803784969 | 1.93298E-11 |
| gene-Led10490-sp3 | -2.074991818 | 7.37038E-11 |
| gene-Led10526-sp3 | -2.212835357 | 5.56712E-20 |
| gene-Led10530-sp3 | 1.086927632 | 4.84463E-05 |
| gene-Led10555-sp3 | -1.402395172 | 6.98771E-08 |
| gene-Led10556-sp3 | 1.322768979 | 0.000712498 |
| gene-Led10562-sp3 | -0.680695416 | 3.65609E-05 |
| gene-Led10567-sp3 | 1.039594917 | 8.60507E-06 |
| gene-Led10581-sp3 | 3.607214559 | 4.52782E-05 |
| gene-Led10588-sp3 | 0.470858172 | 0.00527267 |
| gene-Led10615-sp3 | -0.625510722 | 1.82739E-05 |
| gene-Led10621-sp3 | 2.725566013 | 4.21239E-06 |
| gene-Led10627-sp3 | -1.073811041 | 1.90336E-06 |
| gene-Led10630-sp3 | -0.703981672 | 0.005128837 |
| gene-Led10639-sp3 | 0.952031399 | 0.000893887 |
| gene-Led10693-sp3 | 2.359199531 | 1.25675E-08 |
| gene-Led10728-sp3 | -0.567368669 | 0.000680871 |
| gene-Led10733-sp3 | 0.838594834 | 9.61579E-05 |
| gene-Led10735-sp3 | 1.049080609 | 3.53062E-06 |
| gene-Led10757-sp3 | -1.200249428 | 7.1704E-05 |

| | | |
|---|---|---|
| gene-Led10760-sp3 | 0.531207508 | 0.003782011 |
| gene-Led10768-sp3 | 1.53015464 | 1.71575E-17 |
| gene-Led10771-sp3 | -0.610551916 | 0.008298145 |
| gene-Led10808-sp3 | -1.926505962 | 0.000127137 |
| gene-Led10813-sp3 | -0.656686864 | 0.000352281 |
| gene-Led10875-sp3 | 0.687240239 | 0.004686808 |
| gene-Led10877-sp3 | -0.699829031 | 0.000924487 |
| gene-Led10878-sp3 | 0.98106957 | 0.00142818 |
| gene-Led10879-sp3 | 0.793845467 | 2.52157E-05 |
| gene-Led10891-sp3 | -1.031286489 | 4.85211E-09 |
| gene-Led10892-sp3 | -3.095225243 | 8.30333E-08 |
| gene-Led10897-sp3 | 1.084567273 | 6.45506E-06 |
| gene-Led10913-sp3 | 3.423791331 | 7.3177E-05 |
| gene-Led10925-sp3 | 1.492250361 | 3.2736E-10 |
| gene-Led10927-sp3 | 0.770805775 | 0.006427538 |
| gene-Led10930-sp3 | -0.554238265 | 0.002200263 |
| gene-Led10936-sp3 | 0.853487033 | 0.000622618 |
| gene-Led10991-sp3 | -2.084032588 | 1.95085E-20 |
| gene-Led10993-sp3 | -0.727146548 | 0.001158677 |
| gene-Led11014-sp3 | 0.604590476 | 0.000676491 |
| gene-Led11023-sp3 | 2.322663126 | 7.14496E-06 |
| gene-Led11071-sp3 | -1.794758635 | 0.002458813 |
| gene-Led11091-sp3 | 0.856273322 | 0.002538886 |
| gene-Led11122-sp3 | -2.943187723 | 3.75977E-10 |
| gene-Led11194-sp3 | 1.041588956 | 0.00933979 |
| gene-Led11203-sp3 | -1.937257998 | 3.44707E-10 |
| gene-Led11234-sp3 | 2.734298119 | 3.76071E-24 |
| gene-Led11282-sp3 | 2.782986473 | 4.89823E-22 |
| gene-Led11319-sp3 | 0.807690905 | 0.001792685 |
| gene-Led11452-sp3 | 1.04088619 | 0.002618091 |
| gene-Led11455-sp3 | -1.338955707 | 0.000172024 |

**Table S6 Common DEGs between Venn diagram**

| |
|---|
| gene-Led00095-sp3 |
| gene-Led00127-sp3 |
| gene-Led00180-sp3 |
| gene-Led00287-sp3 |
| gene-Led00345-sp3 |
| gene-Led00553-sp3 |
| gene-Led00680-sp3 |
| gene-Led00683-sp3 |
| gene-Led00910-sp3 |
| gene-Led00929-sp3 |
| gene-Led01098-sp3 |
| gene-Led01338-sp3 |
| gene-Led01347-sp3 |
| gene-Led01536-sp3 |
| gene-Led01538-sp3 |
| gene-Led01568-sp3 |
| gene-Led01764-sp3 |
| gene-Led01765-sp3 |
| gene-Led01978-sp3 |
| gene-Led02063-sp3 |
| gene-Led02397-sp3 |
| gene-Led02690-sp3 |
| gene-Led02755-sp3 |
| gene-Led02776-sp3 |
| gene-Led03250-sp3 |
| gene-Led03434-sp3 |
| gene-Led03576-sp3 |
| gene-Led03682-sp3 |
| gene-Led03887-sp3 |
| gene-Led03896-sp3 |
| gene-Led03898-sp3 |
| gene-Led03907-sp3 |
| gene-Led04121-sp3 |
| gene-Led04279-sp3 |
| gene-Led04345-sp3 |
| gene-Led04429-sp3 |
| gene-Led04499-sp3 |
| gene-Led04617-sp3 |
| gene-Led04683-sp3 |
| gene-Led04763-sp3 |
| gene-Led04796-sp3 |
| gene-Led04806-sp3 |
| gene-Led04949-sp3 |

| |
|---|
| gene-Led05017-sp3 |
| gene-Led05022-sp3 |
| gene-Led05031-sp3 |
| gene-Led05063-sp3 |
| gene-Led05068-sp3 |
| gene-Led05095-sp3 |
| gene-Led05130-sp3 |
| gene-Led05133-sp3 |
| gene-Led06015-sp3 |
| gene-Led06338-sp3 |
| gene-Led06391-sp3 |
| gene-Led06524-sp3 |
| gene-Led06546-sp3 |
| gene-Led06582-sp3 |
| gene-Led06888-sp3 |
| gene-Led06957-sp3 |
| gene-Led07102-sp3 |
| gene-Led07148-sp3 |
| gene-Led07155-sp3 |
| gene-Led07224-sp3 |
| gene-Led07517-sp3 |
| gene-Led07525-sp3 |
| gene-Led07526-sp3 |
| gene-Led07626-sp3 |
| gene-Led08187-sp3 |
| gene-Led08218-sp3 |
| gene-Led08460-sp3 |
| gene-Led08636-sp3 |
| gene-Led08734-sp3 |
| gene-Led08827-sp3 |
| gene-Led09023-sp3 |
| gene-Led09321-sp3 |
| gene-Led09418-sp3 |
| gene-Led09424-sp3 |
| gene-Led09450-sp3 |
| gene-Led10484-sp3 |
| gene-Led10555-sp3 |
| gene-Led10768-sp3 |
| gene-Led10927-sp3 |
| gene-Led11122-sp3 |

**Table S7 The top 20 enriched GO termsof DEGs in LeHH24h vs LeHH0h**

| ID | Descrption | Pvalue | Qvalue | Up | Down |
|---|---|---|---|---|---|
| GO:0055114 | oxidation-reduction process | 1.09E-09 | 6.61E-07 | 60 | 34 |
| GO:0005618 | cell wall | 1.15E-06 | 7.07E-05 | 8 | 0 |
| GO:0009277 | fungal-type cell wall | 1.15E-06 | 7.07E-05 | 8 | 0 |
| GO:0030312 | external encapsulating structure | 1.15E-06 | 7.07E-05 | 8 | 0 |
| GO:0000786 | nucleosome | 5.76E-05 | 0.0026505 | 8 | 0 |
| GO:0032993 | protein-DNA complex | 0.000164 | 0.0060359 | 8 | 0 |
| GO:0044815 | DNA packaging complex | 0.0002593 | 0.0079505 | 8 | 0 |
| GO:0005840 | ribosome | 0.0003698 | 0.0097197 | 20 | 1 |
| GO:0000785 | chromatin | 0.0005984 | 0.0137635 | 9 | 0 |
| GO:0005743 | mitochondrial inner membrane | 0.0007475 | 0.0152832 | 10 | 0 |
| GO:0019866 | organelle inner membrane | 0.0009807 | 0.0179978 | 10 | 0 |
| GO:0005740 | mitochondrial envelope | 0.001076 | 0.0179978 | 13 | 0 |
| GO:0098800 | inner mitochondrial membrane protein complex | 0.0011803 | 0.0180983 | 8 | 0 |
| GO:0030529 | intracellular ribonucleoprotein complex | 0.0013996 | 0.0198103 | 23 | 2 |
| GO:1990904 | ribonucleoprotein complex | 0.001564 | 0.0205556 | 23 | 2 |
| GO:0071944 | cell periphery | 0.00168 | 0.0206075 | 10 | 1 |
| GO:0098798 | mitochondrial protein complex | 0.0024742 | 0.0284532 | 9 | 0 |
| GO:0016491 | oxidoreductase activity | 6.33E-11 | 2.08E-08 | 62 | 40 |
| GO:0005199 | structural constituent of cell wall | 7.34E-07 | 0.0001208 | 8 | 0 |
| GO:0005198 | structural molecule activity | 2.13E-05 | 0.0023376 | 28 | 1 |

**Table S8 The top 20 enriched KEGG terms of DEGs in LeHH24h vs LeHH0h**

| KEGG_A class | Pathway | out (217) | All (2086) | Pvalue | Qvalue | Pathway ID |
|---|---|---|---|---|---|---|
| Genetic Information Processing | Ribosome | 24 | 94 | 1.48E-05 | 0.001421 | ko03010 |
| Metabolism | Glycolysis / Gluconeogenesis | 13 | 45 | 0.000412 | 0.01977 | ko00010 |
| Metabolism | Methane metabolism | 8 | 22 | 0.00106 | 0.033909 | ko00680 |
| Metabolism | Carbon metabolism | 20 | 106 | 0.005008 | 0.088727 | ko01200 |
| Metabolism | Tyrosine metabolism | 7 | 22 | 0.005206 | 0.088727 | ko00350 |
| Metabolism | Metabolic pathways | 113 | 912 | 0.005545 | 0.088727 | ko01100 |
| Metabolism | Glyoxylate and dicarboxylate metabolism | 7 | 24 | 0.008815 | 0.114572 | ko00630 |
| Metabolism | Oxidative phosphorylation | 14 | 69 | 0.009548 | 0.114572 | ko00190 |
| Metabolism | Biosynthesis of secondary metabolites | 50 | 358 | 0.011634 | 0.124092 | ko01110 |
| Metabolism | Glycerolipid metabolism | 7 | 29 | 0.025352 | 0.243383 | ko00561 |
| Metabolism | Linoleic acid metabolism | 2 | 3 | 0.030107 | 0.262752 | ko00591 |
| Metabolism | Fatty acid degradation | 7 | 31 | 0.035724 | 0.285791 | ko00071 |
| Metabolism | Ascorbate and aldarate metabolism | 4 | 15 | 0.06202 | 0.457996 | ko00053 |
| Metabolism | Fructose and mannose metabolism | 5 | 24 | 0.095874 | 0.607639 | ko00051 |
| Metabolism | Sulphur metabolism | 5 | 24 | 0.095874 | 0.607639 | ko00920 |
| Metabolism | Sesquiterpenoid and triterpenoid biosynthesis | 1 | 1 | 0.104027 | 0.607639 | ko00909 |
| Metabolism | Biosynthesis of unsaturated fatty acids | 4 | 18 | 0.108918 | 0.607639 | ko01040 |
| Metabolism | Glycosphingolipid biosynthesis - globo and isoglobo series | 2 | 6 | 0.122046 | 0.607639 | ko00603 |
| Metabolism | Pyruvate metabolism | 8 | 49 | 0.129558 | 0.607639 | ko00620 |
| Cellular Processes | Mitophagy - yeast | 6 | 34 | 0.134449 | 0.607639 | ko04139 |
